# NHC-catalyzed cleavage of vicinal diketones and triketones followed by insertion of enones and ynones

**DOI:** 10.3762/bjoc.13.176

**Published:** 2017-08-30

**Authors:** Ken Takaki, Makoto Hino, Akira Ohno, Kimihiro Komeyama, Hiroto Yoshida, Hiroshi Fukuoka

**Affiliations:** 1 Department of Applied Chemistry, Graduate School of Engineering, Hiroshima University, Kagamiyama, Higashi-Hiroshima 739-8527, Japan

**Keywords:** Breslow intermediate, *N*-heterocyclic carbene, ring enlargement, Stetter reaction, vicinal polyketone

## Abstract

Thiazolium carbene-catalyzed reactions of 1,2-diketones and 1,2,3-triketones with enones and ynones have been investigated. The diketones gave α,β-double acylation products via unique Breslow intermediates isolable as acid salts, whereas the triketones formed stable adducts with the NHC instead of the coupling products.

## Introduction

**Scheme 1:**
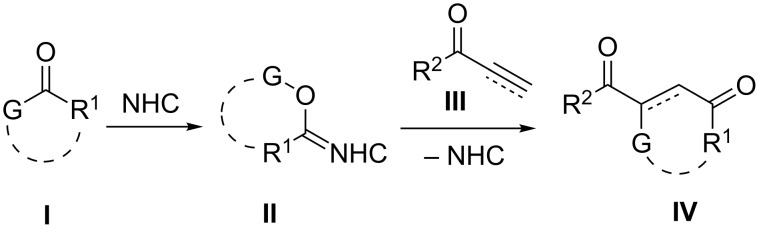
Reaction process.

*N*-Heterocyclic carbenes (NHCs) have been indispensable catalysts for organic synthesis, particularly for umpolung of various functional groups [1–9]. In the Stetter reaction, NHCs convert aldehydes to nucleophilic species, which react with activated alkenes to yield hydroacylation products [10–14]. When the carbonyl compounds **I** other than aldehyde behave similarly, functionalized 1,4-diketones **IV** would be produced (Scheme 1). Previously, we reported that benzils **I** (G = C(O)R^1^) reacted with enones **III** in the presence of thiazolium carbene catalysts to give double acylation products **IV** in good yields [15]. If enones can be replaced by ynones **III** in the reaction with benzils, alkenes **IV** having three acyl moieties would be formed directly. Related products were recently obtained by the dimsyl anion-promoted double acylation of enones with benzils, followed by dehydrogenation of the resulting alkanes in one pot [16]. Moreover, if the reaction of cyclic 1,2-diketone **I** (G = C(O)R^1^) with activated alkenes may take place similarly, this reaction could be utilized as a ring-enlargement procedure to afford cyclic 1,4-diketones **IV**.

With respect to the active species in the Stetter reaction, aminoenols **II** (G = H, Breslow intermediates) had been postulated as true nucleophiles for a long time and those generated from imidazolinium NHC were recently isolated in pure form [17–18]. In the reaction of benzils with thiazolium NHC, aminoenol esters **II** (G = C(O)R^1^) could be formed similarly, but this active species were found to exist as isolable acid salts unexpectedly. Moreover, the reactivity of 1,2,3-triketones was also investigated in comparison with that of 1,2-diketones. We would like to report herein these results.

## Results and Discussion

**Figure 1:**
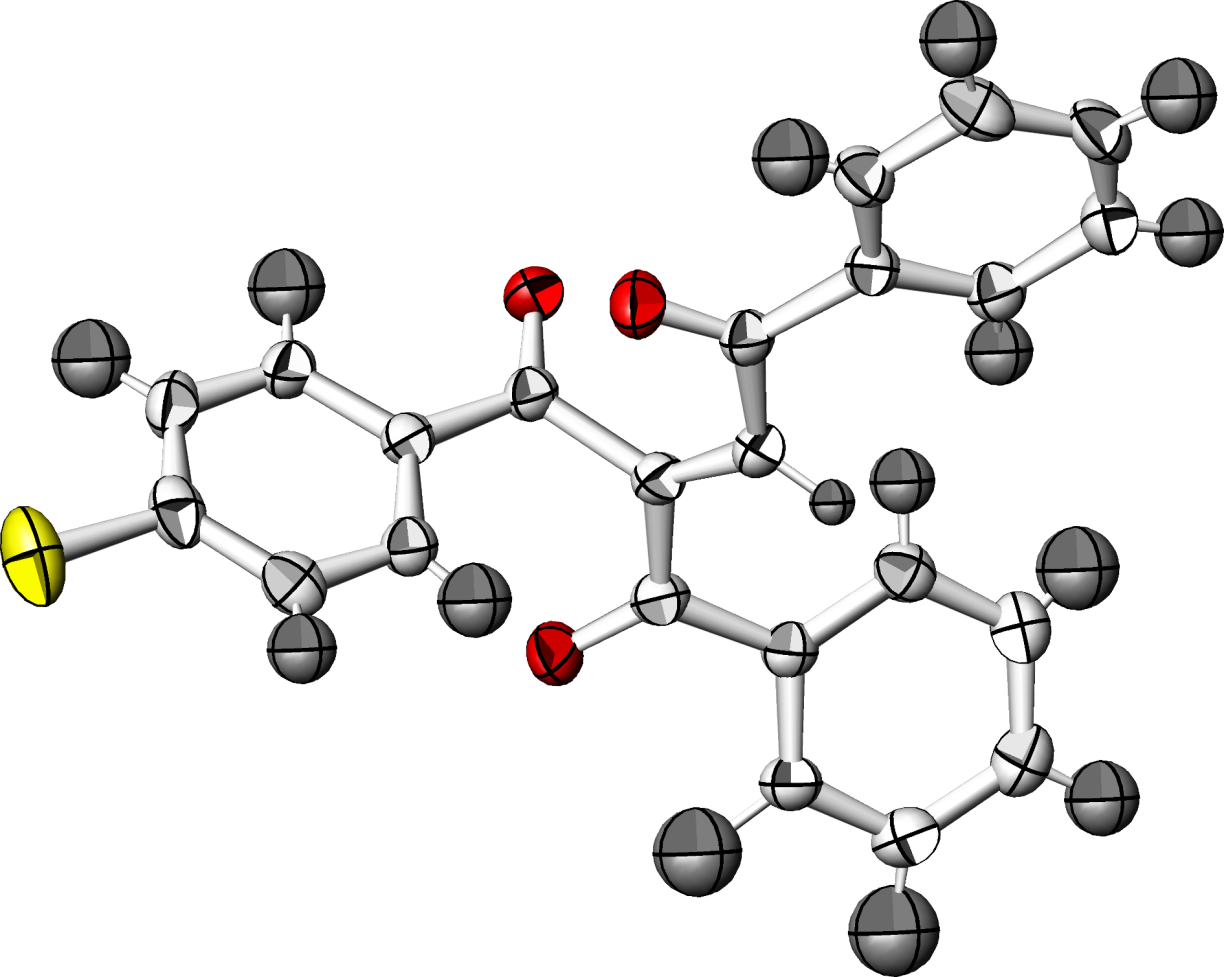
ORTEP drawing of *Z*-**4ai**.

The reaction of benzil (**1a**) with various ynones **2** was carried out by use of thiazolium salt **3** under similar conditions to that with enones (Table 1) [15]. In the reaction of **1a** with 1-phenylprop-2-yn-1-one (**2a**), tribenzoylethylene (**4aa**) was formed in 64% yield (Table 1, entry 1). Fortunately possible propargylic alcohols by cross-benzoin reaction were not detected [19]. The other products of **4** were obtained as inseparable mixtures of *E*- and *Z*-isomer, except for **4ae** and **4ar**, but their stereochemistry could not be determined by NMR. Only the major isomer of **4ai** crystallized from the mixture and thus *Z*-stereochemistry of the two aroyl groups (ClC_6_H_4_CO and PhCO) was confirmed by X-ray analysis (Figure 1) [20]. Electron-donating substituents of ynones **2** gave slightly better yields than electron-withdrawing ones. The yields and isomer ratios were also affected by the position of the substituents on the aroyl groups, i.e., *ortho*-substituents decreased the product yields and increased the isomer ratios (Table 1, entries 2–4 and 7–9). Mesityl and aliphatic ynones **2e** and **2r** gave the single isomers **4ae** and **4ar** in low yields (Table 1, entries 5 and 18).

**Table 1:** Reaction of benzil (**1a**) with ynones **2**^a^.

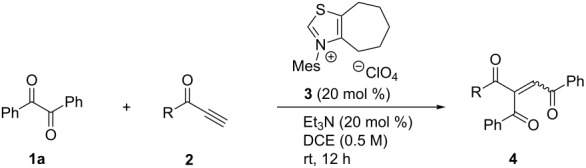					

Entry	Ynone **2**	R	Product **4**	Yield (%)^b^	Isomer ratio^c^

1	**2a**	Ph	**4aa**	64	–
2	**2b**	2-MeC_6_H_4_	**4ab**	54	74:26
3	**2c**	3-MeC_6_H_4_	**4ac**	64	52:48
4	**2d**	4-MeC_6_H_4_	**4ad**	63	52:48
5	**2e**	2,4,6-Me_3_C_6_H_2_	**4ae**	32	–^d^
6	**2f**	4-MeOC_6_H_4_	**4af**	63	53:47
7	**2g**	2-ClC_6_H_4_	**4ag**	31	80:20
8	**2h**	3-ClC_6_H_4_	**4ah**	49	61:39
9	**2i**	4-ClC_6_H_4_	**4ai**	53	60:40^e^
10	**2j**	4-BrC_6_H_4_	**4aj**	57	59:41
11	**2k**	4-MeO_2_CC_6_H_4_	**4ak**	44	59:41
12	**2l**	4-NCC_6_H_4_	**4al**	20	71:29
13	**2m**	2-furyl	**4am**	41	65:35
14	**2n**	2-thienyl	**4an**	54	67:33
15	**2o**	3-thienyl	**4ao**	61	67:33
16	**2p**	1-naphthyl	**4ap**	52	69:31
17	**2q**	2-naphthyl	**4aq**	50	53:47
18	**2r**	Ph(CH_2_)_2_	**4ar**	13	–^d^

^a^Equimolar amounts of **2** were used. ^b^Isolated yields. ^c^Estimated by NMR. ^d^One stereoisomer was formed. ^e^*E*/*Z* = 40:60 determined by X-ray analysis.

Then, the substituent effect of benzils **1** was investigated (Table 2). Electron-donating groups decreased the reaction efficiency slightly. The yields were improved by catalytic amounts of MgCl_2_ as reported previously [15], whereas the isomer ratios of **4** were nearly unchanged irrespective of the Lewis acid. When a mixture of benzils **1b** and **1c** (0.5 equiv each) was treated with **2a** (1.0 equiv) under similar conditions, the products **4ba** and **4ca** were formed in 52% and 20% yields, respectively. However, cross products having three different aroyl groups were not detected at all. Thus, the present reaction was proved to take place by an intramolecular process.

**Table 2:** Reaction of substituted benzils **1** with ynone **2a**^a^.

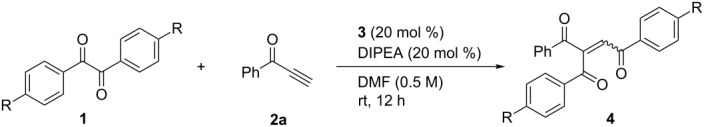					

Entry	Benzil **1**	R	Product **4**	Yield (%)^b,c^	Isomer ratio

1	**1b**	Me	**4ba**	48 (76)	50:50
2	**1c**	OMe	**4ca**	16 (25)	50:50
3	**1d**	Cl	**4da**	56 (74)	53:47
4	**1e**	Br	**4ea**	42 (63)	58:42

^a^Equimolar amounts of **2** were used. ^b^Isolated yields. ^c^Yields in parentheses were observed in the presence of MgCl_2_ (20 mol %).

**Scheme 2:**
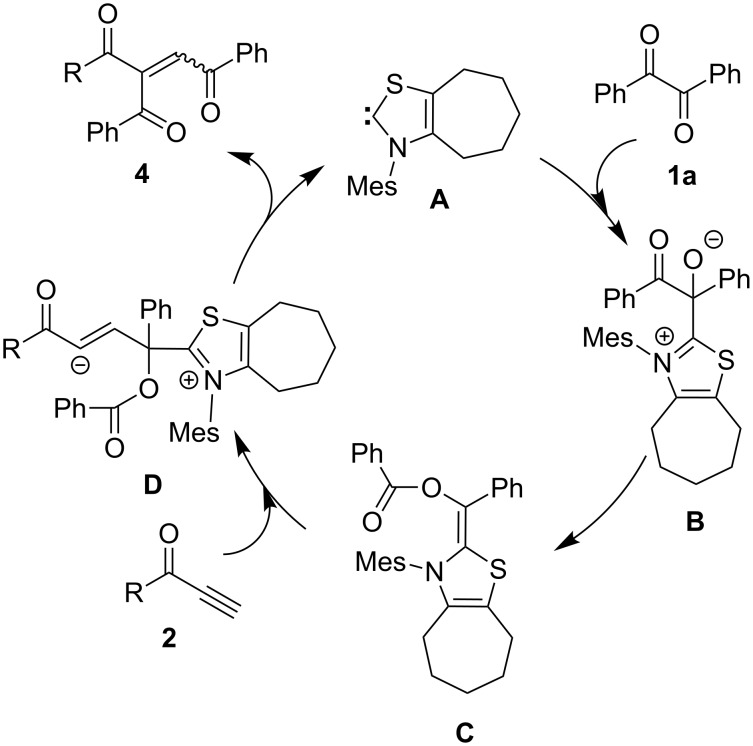
Reaction mechanism.

A reaction mechanism is proposed in Scheme 2, which is similar to that with enones [15]. The monoacylated Breslow intermediate **C** is formed by addition of thiazolium NHC **A** to benzil (**1a**), followed by migration of the benzoyl group. Then, this nucleophilic species **C** reacts with ynones **2** to generate the intermediate **D**. The second migration of the benzoyl group to the α-position of **2** and simultaneous elimination of **A** affords product **4**.

**Scheme 3:**
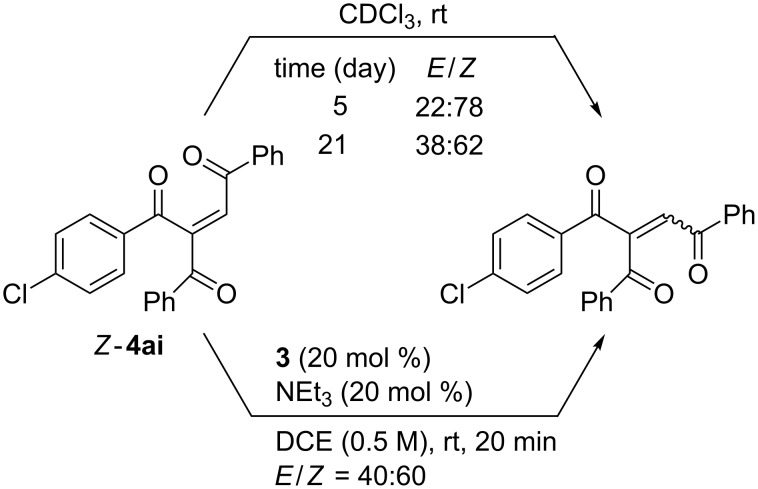
Isomerization of the stereochemistry of **4ai**.

According to the intramolecular mechanism shown in Scheme 2, the two benzoyl groups of **1a** should be delivered to the ynones **2** from the same side to yield *E*-products **4** exclusively. However, **4** were obtained as a mixture of *E-* and *Z-*isomer in the most cases. Since pure *Z*-isomer of the product **4ai** was exceptionally isolated, its isomerization was tested in order to explain the stereochemistry (Scheme 3). When the *Z*-**4ai** was dissolved in CDCl_3_ and monitored by NMR, the ratio of *E/Z* changed slowly to 38:62 after 21 days. In contrast, the original ratio of 40:60 was quickly attained in the presence of the NHC catalyst **A**.

**Scheme 4:**
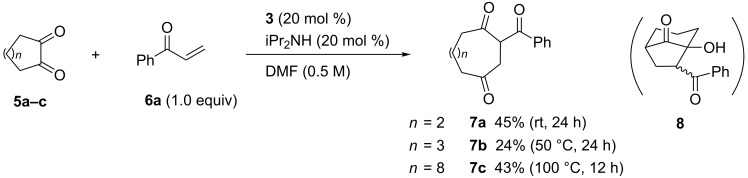
Reaction of cycloalkane-1,2-diones with phenyl vinyl ketone (**6a**).

We demonstrated previously the reaction of cyclohexane-1,2-dione (**5a**) with phenyl vinyl ketone (**6a**) to afford 2-benzoylcyclooctane-1,4-dione (**7a**) in 27% yield [15]. Since this reaction would be a unique ring enlargement by two carbons, an improvement of the reaction conditions has been tested. Although the yield increased to 45% by the use of diisopropylamine instead of DIPEA, the reaction efficiency was much lower than that of benzil (**1a**) (Scheme 4). The only isolated by-product in the reaction of **5a** was bicyclo[3.2.1]octanone **8** (ca. 10%), which was formed by competitive tandem Michael–aldol reaction [21]. Cycloheptane-1,2-dione (**5b**) and cyclododecane-1,2-dione (**5c**) gave the 9- and 14-membered 1,4-diketones **7b** and **7c** at elevated temperature, respectively.

**Scheme 5:**
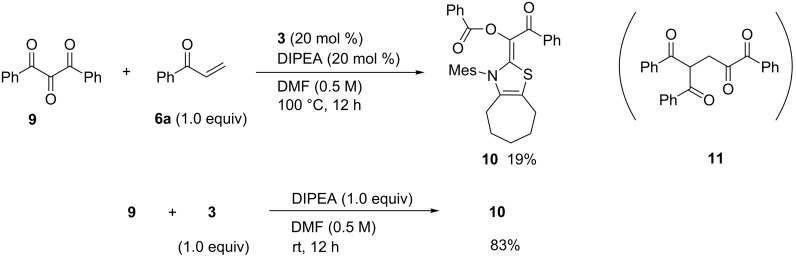
Preparation and reactivity of the bisacylated Breslow intermediate **10**.

**Figure 2:**
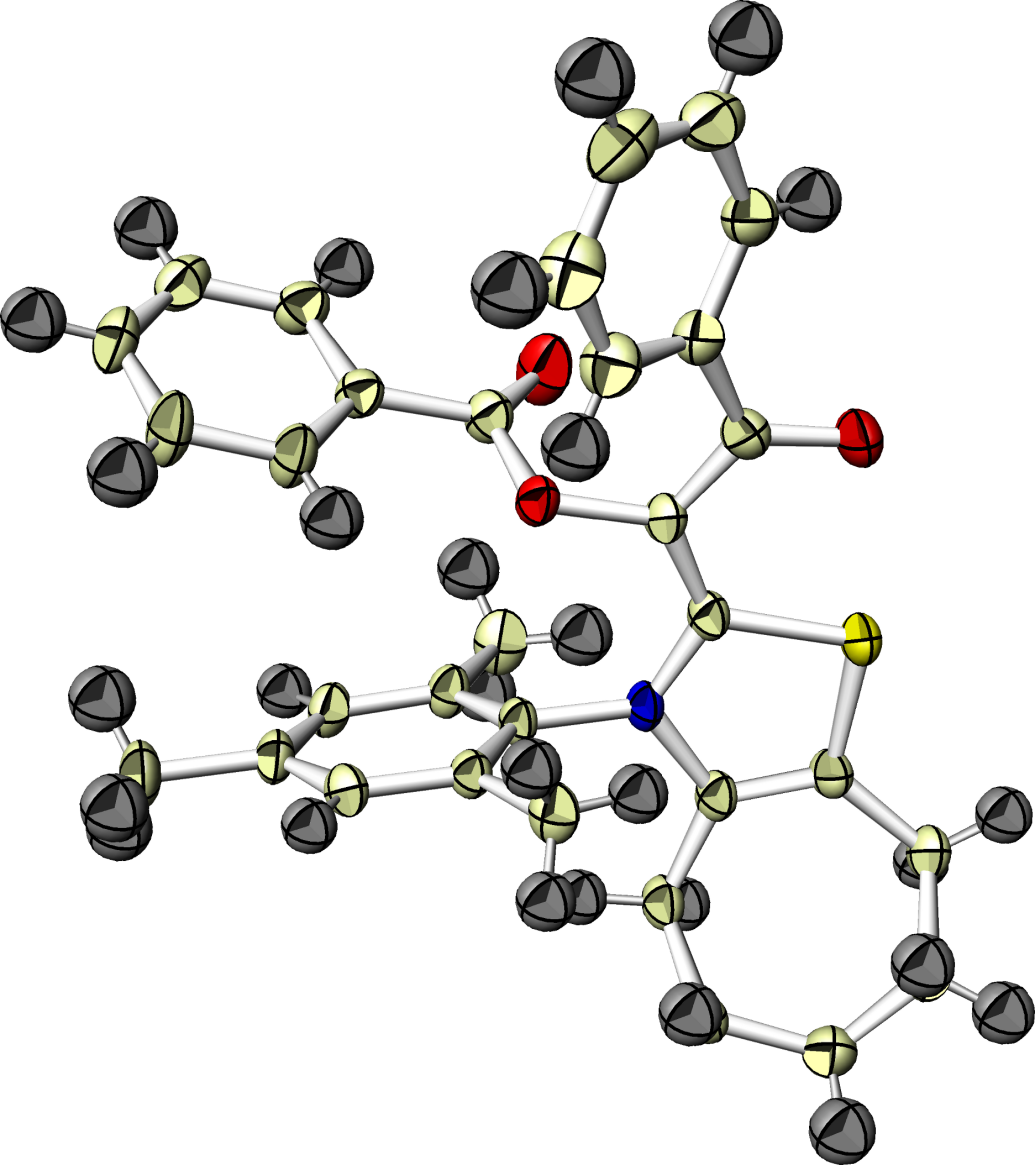
ORTEP drawing of **10**.

Next, we investigated the NHC-catalyzed reaction of 1,2,3-triketone **9** with enone **6a**. Since the central carbonyl group of **9** could be more electrophilic than the others at 1- and 3-positions, nucleophilic addition of NHC followed by migration of one neighboring acyl group to the central carbonyl oxygen would generate bisacylated Breslow intermediate **10** (Scheme 5). If this species behaves in a similar manner as the monoacylated intermediate **C** derived from 1,2-diketone **1**, its reaction with enone **6a** would be expected to yield tetraketone **11**. However, when triketone **9** was treated with equimolar amounts of **6a** in the presence of **3** and DIPEA (20 mol %, each), the predicted intermediate **10** was obtained in 19% yield based on **9**, but any coupling products of **9** with **6a** such as **11** were not formed. The yield of **10** increased to 83% in the equimolar reaction of **9** with **3**. In addition, the product **10** was recovered unchanged after treatment with **6a** irrespective of DIPEA. The nucleophilicity of **10** may be canceled by the two benzoyl groups. The product **10** was fairly stable to air and moisture, and thus *E*-structure was confirmed by X-ray analysis as shown in Figure 2 [20].

**Scheme 6:**
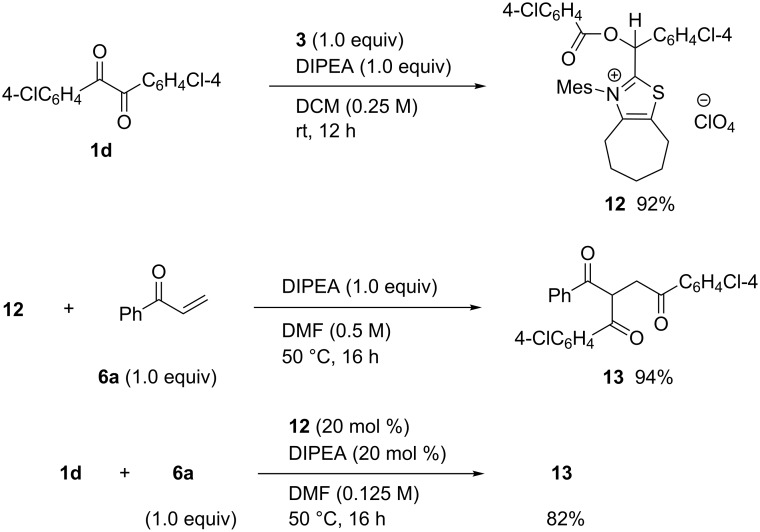
Preparation of the iminium salt **12** and its reactivity.

Isolation of **10** prompted us to get the monoacylated Breslow intermediate **C** also as a stable compound. Treatment of benzil **1d** with equimolar amounts of **3** and DIPEA at room temperature, followed by non-aqueous work-up and column chromatography gave crystalline product **12** in 92% yield (Scheme 6). Although **12** did not provide single crystals suitable for X-ray analysis, its structure was determined by NMR and elemental analysis. ^1^H NMR spectra of **12** showed eleven protons in the aromatic region, i.e., two ClC_6_H_4_, Me_3_C_6_H_2_, and one particular proton at δ 6.68. In ^13^C NMR spectra, one unknown signal appeared in the neighborhood of the ester carbon (δ 163.1 and 167.1). Moreover, an unusual signal appeared at δ 71.5. These data indicated definitely that the isolated product **12** was different from the expected intermediate **C**. Instead, the iminium salt **12**, that is, the HClO_4_ salt of **C**, could account for all unusual signals. Furthermore, elemental analysis of **12** agreed very closely with its theoretical value and deviated much from that of **C**.

After identification of the product **12**, we noticed that Massi and his group obtained the similar adducts during their study on the multicomponent reaction of thiazolium carbenes, benzils and water to yield 1,4-thiazin-3-ones [22]. Although the initial process producing the salt **12** was similar, the total mode of the two reactions was quite different, that is, stoichiometric vs catalytic reaction with respect to the thiazolium carbenes. In the Massi reaction, monoacylated Breslow intermediate **C** was readily hydrolyzed by hydroxide, whereas Stetter reaction took place exclusively in our reaction. The difference would be caused by the reaction conditions, particularly by the solvent system.

**Scheme 7:**
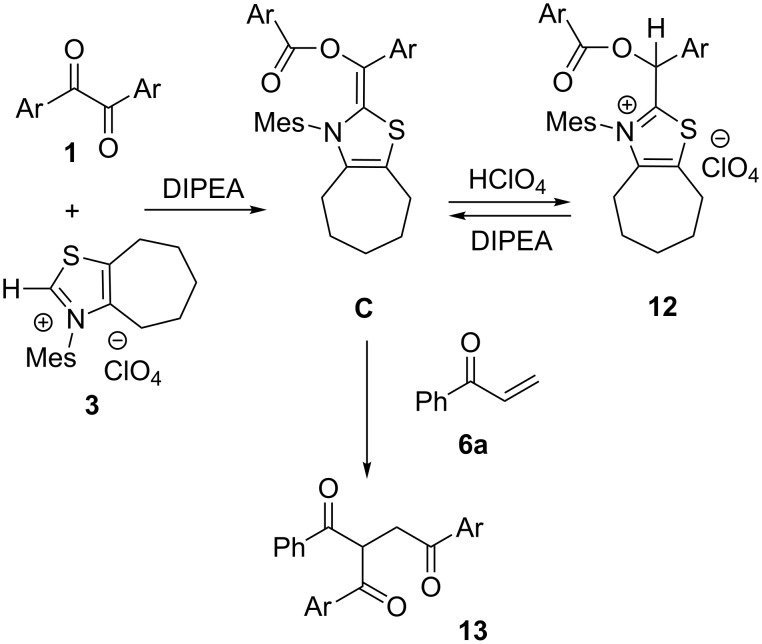
Resting state of the monoacylated Breslow intermediate **C**.

Since the product **12** was obtained unexpectedly, its reactivity and role in the catalytic cycle were investigated. The stoichiometric reaction of **12** with phenyl vinyl ketone (**6a**) in the presence of DIPEA gave the product **13** in 94% yield, while no product was formed without the base. Moreover, the reaction of benzil **1d** with enone **6a** was catalyzed by **12** to give the product **13** in good yield, which are comparable results to the use of **3** [15]. These results suggested that compound **12** played a resting state of the monoacylated Breslow intermediate **C** as shown in Scheme 7. Accordingly, when enone **6a** was present in the mixture, facile regeneration of **C** and its reaction with **6a** took place predominantly. It seems surprising that compound **12** could be quantitatively generated from **C** and HClO_4_ regardless of equimolar amounts of DIPEA, while analogous intermediates derived from imidazole NHC were protonated by the acid alone [23].

## Conclusion

We have demonstrated the thiazolium NHC-catalyzed reaction of benzils with ynones to give triacylated alkenes in fairly good yields. When this reaction was applied to aliphatic cyclic diketones with enones, two carbon ring-enlarged products were formed, though in low yields. Moreover, aromatic 1,2,3-triketones reacted with NHCs to afford bisacylated Breslow intermediates in high yield. However, their nucleophilicity was so weak that they were recovered unchanged in the reaction with enones. It was also found that the monoacylated Breslow intermediates changed reversibly to the resting state of acid salts.

## Supporting Information

File 1 Experimental procedure, characterization data and copies of ^1^H and ^13^C NMR spectra of the products.
