# Supplementary material for: NHC-catalyzed cleavage of vicinal diketones and triketones followed by insertion of enones and ynones
Source: Beilstein J Org Chem. 2017 Aug 30;13:1816–22. doi: 10.3762/bjoc.13.176 (PMC5588593; doi:10.3762/bjoc.13.176)

Supporting Information

for

NHC-catalyzed cleavage of vicinal diketones and triketones followed by insertion of enones and ynones

Ken Takaki*, Makoto Hino, Akira Ohno, Kimihiro Komeyama, Hiroto Yoshida and Hiroshi Fukuoka

Address: Department of Applied Chemistry, Graduate School of Engineering, Hiroshima University, Kagamiyama, Higashi-Hiroshima 739-8527, Japan

Email: Ken Takaki - ktakaki@hiroshima-u.ac.jp

* Corresponding author

Experimental procedure, characterization data and copies of ^{1}H and ^{13}C NMR spectra of the products

Table of contents

I. General information S2

II. General procedure for the reaction of **1** with **2** and characterization data of **4** S2

III. General procedure for the reaction of **5** with **6** and characterization data of **7** S11

IV. Synthesis and characterization data of **10** S13

V. Synthesis and characterization data of **12** S14

VI. Reaction of **1d** with **6a** catalyzed by **12** and DIPEA S14

VII. References S15

VIII. Copies of ^{1}H and ^{13}C NMR spectra of the products S16

I. General information

NMR spectra were taken on a Varian 400-MR (^{1}H, 399.82 MHz; ^{13}C, 100.54 MHz) spectrometer or Varian 500-MR (^{1}H, 499.82 MHz; ^{13}C, 125.68 MHz) spectrometer using residual chloroform (^{1}H, δ = 7.26) or $CDCl_3$ (^{13}C, δ = 77.0) as an internal standard. Mass spectra were obtained at 70 eV on a Simadzu GC-MS QP5050 spectrometer. High-resolution mass spectra were recorded with Thermo Fisher Scientific LTQ Orbitrap XL (ESI or APCI/FTMS mode). Melting points were measured on a Shimadzu melting point apparatus and are uncorrected. TLC monitoring was performed with Merck silica gel (60 F_{254}). All reactions were conducted with a standard Schlenk technique under nitrogen or argon atmosphere. DMF, DCM and DCE solvents were distilled from CaH. Et_3N, DIPEA and iPr_2NH bases were distilled from molecular sieves 4 Å. $MgCl_2$ was dried by heat gun under vacuum.

II. General procedure for the reaction of **1** with **2** and characterization data of **4**

A Schlenk tube was charged with thiazolium salt **3** (37 mg, 0.10 mmol), benzil (**1a**, 105 mg, 0.50 mmol), 1-(4-chlorophenyl)prop-2-yn-1-one (**2i**, 82 mg, 0.50 mmol), DCE (1.0 mL), and Et_3N (14 μL, 0.10 mmol) under N_2. The mixture was stirred at room temperature for 12 h. After quenching with water, accurately weighted diphenylmethane (35.0 mg, 0.208 mmol) was added to the mixture as an internal standard. The organic layer was extracted with ether, washed with brine, dried over $MgSO_4$, and concentrated under vacuum. The residue was measured by ^{1}H NMR to determine the yield of **4ai** (65% yield). Then, the crude product was purified by column chromatography on silica gel with hexane-EtOAc eluent to give a mixture of *E*- and *Z*-isomer (99 mg, 53% yield). Although the *E*/*Z* ratio (40:60 or the reverse) could be estimated by ^{13}C NMR spectra of the mixture [1], it was difficult to separate each other and determine which is which. Major isomer of **4ai** crystalized from the mixture exceptionally and its *Z*-structure was confirmed by X-ray analysis.

2-Benzoyl-1,4-diphenylbut-2-ene-1,4-dione (4aa) [2]
Isolated in 64% yield, yellow oil: R_f = 0.33 (hexane / EtOAc = 5 / 1); ^{1}H NMR ($CDCl_3$, 399.82 MHz) δ 7.42 (4H, t, *J*= 7.6 Hz), 7.48-7.57 (5H, m), 7.62 (1H, t, *J*=

7.4 Hz), 7.87 (2H, d, *J*= 7.6 Hz), 7.99 (2H, d, *J*= 7.2 Hz), 8.04 (2H, d, *J*= 8.0 Hz); ^{13}C NMR ($CDCl_3$, 100.54 MHz) δ 128.59, 128.63, 128.7, 128.8, 128.9, 130.1, 130.8, 133.6, 134.0, 135.4, 135.7, 136.0, 152.5, 188.5, 192.7, 193.8 (one carbon was obscured); MS *m*/*z* 340 (35), 247 (37), 105 (100); HRMS m/z ($[M+Na]^+$) calcd for $C_{23}H_{16}O_3Na$, 363.0992, found 363.0994.

2-Benzoyl-4-phenyl-1-(*o*-tolyl)but-2-ene-1,4-dione (4ab)

Isolated in 54% yield as a mixture of two stereoisomers (74 / 26), yellow oil: R_f = 0.47 and 0.41 (hexane / EtOAc = 5 / 1); ^{1}H NMR ($CDCl_3$, 499.82 MHz) δ 2.48 (3H, s, major C*H*$_3$), 2.64 (3H, s, minor C*H*$_3$), 7.17 (1H, t, *J*= 7.5 Hz), 7.19 (1H, t, *J*= 8.0 Hz), 7.29 (2H, t, *J*= 7.5 Hz), 7.32 (1H, t, *J*= 7.5 Hz), 7.38-7.56 (14H, m), 7.60 (1H, t, *J*= 7.5 Hz,), 7.70 (1H, d, *J*= 7.5 Hz), 7.80-7.84 (5H, m), 7.94 (2H, d, *J*= 7.5 Hz), 8.01 (2H, d, *J*= 7.5 Hz); ^{13}C NMR ($CDCl_3$, 125.68 MHz) δ 20.3 (major), 21.3 (minor), 125.5, 128.54, 128.58, 128.63, 128.66, 128.71, 128.8, 129.7, 130.0, 131.3, 131.5, 131.6, 131.81, 131.83, 132.3, 133.1, 133.5, 133.86, 133.90, 134.1, 134.9, 135.7, 135.8, 135.98, 136.03, 136.1, 138.3, 140.3, 152.1, 152.6, 188.9 (major), 189.3 (minor), 193.2 (minor), 194.5 (major), 195.1 (minor), 195.5 (major) (two carbons were obscured); MS *m*/*z* 354 (7), 338 (7), 247 (26), 119 (82), 105 (100); HRMS m/z ($[M+Na]^+$) calcd for $C_{24}H_{18}O_3Na$, 377.1148, found 377.1152.

2-Benzoyl-4-phenyl-1-(*m*-tolyl)but-2-ene-1,4-dione (4ac)

Isolated in 64% yield as a mixture of two stereoisomers (52 / 48), yellow oil: R_f = 0.41 (hexane / EtOAc = 5 / 1); ^{1}H NMR ($CDCl_3$, 499.82 MHz) δ 2.33 (3H, s, minor CH_3), 2.39 (3H, s, major CH_3), 7.28-7.33 (2H, m), 7.36-7.43 (8H, m), 7.47-7.56 (7H, m), 7.60 (1H, t, *J*= 7.5 Hz), 7.77 (1H, d, *J*= 7.5 Hz), 7.82-7.87 (7H, m), 7.99 (2H, d, *J*= 7.5 Hz), 8.04 (2H, d, *J*= 7.5 Hz); ^{13}C NMR ($CDCl_3$, 125.68 MHz) δ 21.2, 126.4, 127.4, 128.4, 128.5, 128.58, 128.66, 128.73, 128.9, 129.2, 130.0, 130.4, 130.8, 130.9, 133.5, 133.92, 133.95, 134.4, 134.8, 135.44, 135.45, 135.65, 135.68, 136.0, 136.1, 138.4, 138.7, 152.47, 152.49, 152.51, 152.53, 188.5, 192.7 (major), 192.9 (major), 193.8 (minor), 193.9 (minor) (four carbons were obscured); MS *m*/*z* 354 (41), 261 (25), 247 (43), 119 (100), 105 (100); HRMS m/z ($[M+Na]^+$) calcd for $C_{24}H_{18}O_3Na$, 377.1148, found 377.1151.

2-Benzoyl-4-phenyl-1-(p-tolyl)but-2-ene-1,4-dione (4ad)

Isolated in 63% yield as a mixture of two stereoisomers (52 / 48), yellow oil: R_f = 0.38 and 0.36 (hexane / EtOAc = 5 / 1); ^{1}H NMR ($CDCl_3$, 499.82 MHz) δ 2.34 (3H, s, minor C*H₃*), 2.40 (3H, s, major C*H₃*), 7.20 (2H, d, *J*= 8.0 Hz), 7.29 (2H, d, *J*= 8.0 Hz), 7.41 (6H, br t, *J*= 8.0 Hz), 7.48-7.51 (5H, m), 7.54 (2H, t, *J*= 7.5 Hz), 7.60 (1H, t, *J*= 7.5 Hz), 7.86-7.90 (6H, m), 7.96 (2H, d, *J*= 8.0 Hz), 8.00 (2H, d, *J*= 8.0 Hz), 8.04 (2H, d, *J*= 7.0 Hz); ^{13}C NMR ($CDCl_3$, 125.68 MHz) δ 21.60 (minor), 21.64 (major), 128.5, 128.60, 128.67, 128.7, 129.0, 129.1, 129.3, 129.4, 130.1, 130.26, 130.29, 130.6, 132.8, 133.3, 133.5, 133.9, 135.5, 135.7, 136.10, 136.11, 144.5, 145.2, 152.6, 152.9, 188.5 (major), 188.5 (minor), 192.2 (major), 192.7 (major), 193.3 (minor), 193.8 (minor) (four carbons were obscured); MS *m*/*z* 354 (18), 338 (8), 261 (11), 247 (33), 119 (100), 105 (91); HRMS m/z ($[M+Na]^+$) calcd for $C_{24}H_{18}O_3Na$, 377.1148, found 377.1152.

2-Benzoyl-1-mesityl-4-phenylbut-2-ene-1,4-dione (4ae)

Isolated in 32% yield as a single stereoisomer, yellow oil: R_f = 0.50 (hexane / EtOAc = 5 / 1); ^{1}H NMR ($CDCl_3$, 499.82 MHz) δ 2.32 (6H, s), 2.33 (3H, s), 6.93 (2H, s), 7.41 (2H, t, *J*= 7.5 Hz), 7.44-7.48 (3H, m), 7.56 (2H, t, *J*= 7.5 Hz), 7.73 (2H, d, *J*= 7.5 Hz), 7.91 (2H, d, *J*= 7.5 Hz); ^{13}C NMR ($CDCl_3$, 125.68 MHz) δ 19.4, 21.1, 128.50, 128.58, 128.64, 128.65, 128.9, 133.5, 133.9, 134.2, 134.4, 135.0, 136.1, 136.2, 139.6, 151.1, 189.2, 195.2, 200.0; MS *m*/*z* 382 (25), 277 (59), 247 (65), 147 (100), 105 (94); HRMS m/z ($[M+Na]^+$) calcd for $C_{26}H_{22}O_3Na$, 405.1461, found 405.1467.

2-Benzoyl-1-(4-methoxyphenyl)-4-phenylbut-2-ene-1,4-dione (4af)

Isolated in 63% yield as a mixture of two stereoisomers (53/47); yellow oil: R_f = 0.23 and 0.20 (hexane / EtOAc = 5 : 1); ^{1}H NMR ($CDCl_3$, 499.82 MHz) δ 3.77 (3H, s, minor CH_3), 3.82 (3H, s, major CH_3), 6.88 (2H, d, *J*= 8.0 Hz), 6.96 (2H, d, *J*= 8.0 Hz), 7.42 (6H, br t, *J*= 7.5 Hz), 7.47-7.51 (5H, m) , 7.54 (2H, t, *J*= 7.5 Hz), 7.60 (1H, t, *J*= 7.5 Hz), 7.87 (4H, dm, *J*= 8.0 Hz), 7.96 (2H, d, *J*= 9.0 Hz), 8.02 (2H, d, *J*= 7.0 Hz), 8.04 (2H, d, *J*= 7.0 Hz), 8.06 (2H, d, *J*= 8.5 Hz); ^{13}C NMR

($CDCl_3$, 125.68 MHz) δ 55.3 (minor), 55.4 (major), 113.8, 114.0, 128.0, 128.48, 128.55, 128.63, 128.70, 128.8, 129.0, 129.2, 130.0, 130.4, 131.4, 132.7, 133.5, 133.86, 133.91, 135.4, 135.6, 136.10, 136.12, 152.6, 153.6, 163.9, 164.4, 188.3 (major), 188.6 (minor), 190.8 (major), 192.0 (minor), 192.8 (minor), 193.9 (major) (three carbons were obscured); MS *m*/*z* 370 (17), 265 (7), 247 (50), 135 (100), 105 (53); HRMS m/z ($[M+Na]^+$) calcd for $C_{24}H_{18}O_4Na$, 393.1097, found 393.1099.

2-Benzoyl-1-(2-chlorophenyl)-4-phenylbut-2-ene-1,4-dione (4ag)

Isolated in 31% yield as a mixture of two stereoisomers (80/20), yellow oil: R_f = 0.30 and 0.22 (hexane / EtOAc = 5 / 1); ^{1}H NMR ($CDCl_3$, 499.82 MHz) δ 7.32-7.61 (20H, m), 7.64 (1H, t, *J*= 7.0 Hz), 7. 81 (2H, d, *J*= 8.0 Hz), 7.89 (3H, d, *J*= 7.5 Hz), 7.95 (2H, d, *J*= 8.0 Hz), 7.99 (2H, d, *J*= 8.0 Hz); ^{13}C NMR ($CDCl_3$, 125.68 MHz) δ 126.9, 127.1, 128.72, 128.76, 128.79, 128.88, 128.92, 129.5, 130.0, 130.4, 130.9, 131.2, 132.0, 132.2, 133.2, 133.7, 134.2, 134.3, 135.4, 135.8, 135.9, 136.0, 136.1, 136.7, 149.9, 189.0 (major), 190.1 (minor), 192.2 (minor), 192.6 (minor), 193.3 (major), 194.1 (major) (seven carbons were obscured); MS *m*/*z* 374 (5), 358 (3), 339 (68), 281 (5), 247 (40), 139 (63), 105 (100); HRMS m/z ($[M+Na]^+$) calcd for $C_{23}H_{15}O_3ClNa$, 396.0602, found 396.0606.

2-Benzoyl-1-(3-chlorophenyl)-4-phenylbut-2-ene-1,4-dione (4ah)

Isolated in 49% yield as a mixture of two stereoisomers (61 / 39), yellow oil: R_f = 0.35 (hexane / EtOAc = 5 / 1); ^{1}H NMR ($CDCl_3$, 499.82 MHz) δ 7.40 (2H, t, *J*= 7.5 Hz), 7.43-7.62 (15H, m), 7.67 (1H, t, *J*= 7.0 Hz), 7.86-7.90 (6H, m), 7.94 (1H, d, *J*= 7.5 Hz), 7.97 (1H, d, *J*= 8.0 Hz), 7.98 (1H, s), 8.01 (1H, s), 8.05 (2H, d, *J*= 8.0 Hz); ^{13}C NMR ($CDCl_3$, 125.68 MHz) δ 127.2, 128.3, 128.7, 128.82, 128.85, 128.89, 128.95, 129.7, 130.0, 130.07, 130.11, 130.8, 131.2, 133.4, 133.7, 133.9, 134.17, 134.19, 134.24, 134.9, 135.1, 135.3, 135.6, 135.9, 136.0, 137.0, 137.3, 151.89, 151.93, 188.28 (major), 188.33 (minor), 191.6 (minor), 192.6 (major), 192.9 (major), 193.5 (minor) (three carbons were obscured); MS *m*/*z* 374 (14), 358 (8), 281 (18), 247 (8), 139 (24), 105 (100); HRMS: m/z ($[M+Na]^+$) calcd for $C_{23}H_{15}ClO_3Na$, 397.0602, found 397.0606.

2-Benzoyl-1-(4-chlorophenyl)-4-phenylbut-2-ene-1,4-dione (4ai)

Isolated in 53% yield as a mixture of *E*- and *Z*-isomers (40 / 60), yellow oil.
Z-isomer: crystallized partially from the mixture on standing: mp. 138-140 °C (from AcOEt / hexane); R_f = 0.38 (hexane / AcOEt = 5 / 1); ^{1}H NMR ($CDCl_3$, 499.82 MHz) δ 7.40 (2H, d, *J*= 8.5 Hz), 7.43 (2H, t, *J*= 8.0 Hz), 7.50-7.53 (3H, m), 7.57 (1H, t, *J*= 7.5 Hz), 7.64 (1H, t, *J*= 8.0 Hz), 7.87 (2H, d, *J*= 8.0 Hz), 7.94 (2H, d, *J*= 8.0 Hz), 8.04 (2H, d, *J*= 8.5 Hz); ^{13}C NMR ($CDCl_3$, 125.68 MHz) δ 128.7, 128.8, 128.9, 129.0, 130.1, 130.3, 130.5, 134.15, 134.18, 135.3, 135.9, 140.0, 152.2, 188.30, 192.6, 192.8 (one carbons was obscured); MS *m*/*z* 374 (13), 358 (11), 281 (10), 247 (20), 139 (33), 105 (100) (measured as a *E* / *Z* mixture); HRMS m/z ($[M+Na]^+$) calcd for $C_{23}H_{15}ClO_3Na$, 397.0602, found 397.0606 (measured as a *E* / *Z* mixture).
E-isomer: R_f = 0.42 (hexane / AcOEt = 5 / 1); ^{1}H NMR ($CDCl_3$, 499.82 MHz) δ 7.43-7.51 (7H, m), 7.56 (1H, t, *J*= 7.5 Hz), 7.60 (1H, t, *J*= 7.5 Hz), 7.89 (2H, d, *J*= 8.0 Hz), 7.98 (2H, d, *J*= 8.0 Hz), 8.01 (2H, d, *J*= 8.5 Hz); ^{13}C NMR ($CDCl_3$, 125.68 MHz) δ 128.7, 128.9, 129.1, 129.2, 130.6, 131.6, 133.78, 133.81, 134.2, 135.6, 136.1, 140.8, 152.8, 188.28, 191.6, 193.6 (one carbon was obscured).

2-Benzoyl-1-(4-bromophenyl)-4-phenylbut-2-ene-1,4-dione (4aj)

Isolated in 57% yield as a mixture of two stereoisomers (59 / 41), yellow oil: R_f = 0.43 and 0.35 (hexane / EtOAc = 5 / 1); ^{1}H NMR ($CDCl_3$, 499.82 MHz) δ 7.42-7.47 (6H, m), 7.51-7.54 (5H, m), 7.57-7.60 (4H, m), 7.63-7.66 (3H, m), 7.86 (2H, d, *J*= 8.5 Hz), 7.88 (4H, d, *J*= 8.0 Hz), 7.91 (2H, d, *J*= 8.5 Hz), 7.98 (2H, d, *J*= 7.5 Hz), 8.04 (2H, d, *J*= 8.0 Hz); ^{13}C NMR ($CDCl_3$, 125.68 MHz) δ 128.7, 128.81, 128.84, 128.9, 129.0, 129.6, 130.1, 130.41, 130.48, 130.51, 130.67, 130.69, 131.6, 131.9, 132.1, 133.7, 134.13, 134.14, 134.2, 134.5, 135.3, 135.5, 135.9, 136.0, 152.2, 152.4, 188.26 (minor), 188.28 (major), 191.7 (minor), 192.6 (major), 193.0 (major), 193.6 (minor) (two carbons were obscured); MS *m*/*z* 418 (11), 325 (8), 247 (34), 183 (29), 105 (100); HRMS m/z ($[M+Na]^+$) calcd for $C_{23}H_{15}BrO_3Na$, 441.0097, found 441.0097.

Methyl 4-(2-benzoyl-4-oxo-4-phenylbut-2-enoyl)benzoate (4ak)

Isolated in 44% yield as a mixture of two stereoisomers (59 / 41), yellow oil: R_f = 0.56 (hexane / EtOAc = 5 / 1); ^{1}H NMR ($CDCl_3$, 399.82 MHz) δ 3.92 (3H, s, major C*H₃*), 3.96 (3H, s, minor C*H₃*), 7.43-7.47 (6H, m), 7.52-7.62 (7H, m), 7.66 (1H, t, *J*= 7.4 Hz), 7.88 (4H, d, *J*= 8.0 Hz), 7.97 (2H, d, *J*= 8.0 Hz), 8.03-8.12 (8H, m), 8.17 (2H, d, *J*= 8.4 Hz); ^{13}C NMR ($CDCl_3$, 125.68 MHz) δ 52.3 (major), 52.5 (minor), 128.66, 128.75, 128.80, 128.83, 128.86, 128.89, 129.78, 129.80, 129.84, 130.1, 130.9, 131.7, 133.7, 134.0, 134.16, 134.22, 134.4, 135.3, 135.6, 135.87, 135.94, 138.86, 138.88, 151.7, 151.9, 165.8, 166.0, 188.3 (major), 188.4 (minor), 192.4 (minor), 192.7 (major), 193.57 (major), 193.64 (minor) (three carbons were obscured); MS *m*/*z* 398 (15), 382 (24), 305 (13), 247 (18), 163 (42), 105 (100); HRMS m/z ($[M+Na]^+$) calcd for $C_{25}H_{18}O_5Na$, 421.1046, found 421.1043.

2-Benzoyl-1-(4-cyanophenyl)-4-phenylbut-2-ene-1,4-dione (4al)

Isolated in 20% yield as a mixture of two stereoisomers (71 / 29), yellow oil: R_f = 0.18 (hexane / EtOAc = 5 / 1); ^{1}H NMR ($CDCl_3$, 499.82 MHz) δ 7.42-7.49 (6H, m), 7.54-7.71 (8H, m), 7.75 (2H, d, *J*= 8.0 Hz), 7.81 (2H, d, *J*= 8.0 Hz), 7.88 (4H, d, *J*= 8.5 Hz), 7.95 (2H, d, *J*= 7.5 Hz), 8.04 (2H, d, *J*= 8.0 Hz), 8.10 (2H, d, *J*= 8.0 Hz), 8.12 (2H, d, *J*= 9.0 Hz); ^{13}C NMR ($CDCl_3$, 100.54 MHz) δ 116.5 (major), 117.1 (minor), 117.6 (minor), 117.9 (major), 128.75, 128.78, 128.82, 128.96, 129.03, 129.3, 130.2, 130.4, 130.5, 131.7, 132.52, 132.53, 134.0, 134.36, 134.45, 134.51, 135.2, 135.5, 135.8, 135.9, 138.7, 138.8, 151.7, 151.8, 188.2, 191.6 (minor), 192.7 (major), 193.0 (major), 193.4 (minor) (three carbons were obscured); MS *m*/*z* 365 (16), 349 (4), 272 (10), 247 (5), 130 (14), 105 (100); HRMS m/z ($[M+Na]^+$) calcd for $C_{24}H_{15}NO_3Na$, 388.0944, found 388.0948.

2-Benzoyl-1-(furan-2-yl)-4-phenylbut-2-ene-1,4-dione (4am)

Isolated in 41% yield as a mixture of two stereoisomers (65 / 35), yellow oil: R_f = 0.22 and 0.15 (hexane / EtOAc = 5 / 1); ^{1}H NMR ($CDCl_3$, 499.82 MHz) δ 6.50 (1H, dd, *J*= 3.5, 1.5 Hz,minor), 6.56 (1H, dd, *J*= 3.5, 1.5 Hz, major), 7.24 (1H, d, *J*= 4.0 Hz), 7.40-7.64 (16H, m), 7.89-7.96 (7H, m), 8.01 (2H, d, *J*= 8.0 Hz); ^{13}C

NMR ($CDCl_3$, 125.68 MHz) δ 112.7, 112.9, 119.2, 122.1, 128.69, 128.74, 128.75, 128.77, 128.80, 128.82, 129.9, 132.6, 133.6, 133.9, 134.06, 134.08, 134.13, 135.7, 136.0, 136.2, 147.1, 148.2, 149.1, 150.3, 151.1, 151.9, 177.4, 180.5, 188.9, 189.5, 192.4, 193.9 (two carbons were obscured); MS *m*/*z* 330 (11), 314 (11), 247 (41), 225 (7), 105 (100); HRMS m/z ($[M+Na]^+$) calcd for $C_{21}H_{14}O_4Na$, 353.0784, found 353.0787.

2-Benzoyl-4-phenyl-1-(thiophen-2-yl)but-2-ene-1,4-dione (4an)

Isolated in 54% yield as a mixture of two stereoisomers (67 / 33), yellow oil: R_f = 0.24 (hexane / EtOAc = 5 / 1); ^1H NMR ($CDCl_3$, 499.82 MHz) δ 7.09 (1H, dd, *J*= 5.0, 4.0 Hz, minor), 7.19 (1H, dd, *J*= 5.0, 4.0 Hz, major), 7.43-7.71 (16H, m), 7.80 (1H, dd, *J*= 4.5, 1.0 Hz), 7.91 (2H, d, *J*= 8.5 Hz), 7.93 (2H, d, *J*= 8.0 Hz), 7.97 (1H, dd, *J*= 4.0, 1.0 Hz), 8.01 (2H, d, *J*= 7.5 Hz), 8.06 (2H, d, *J*= 7.5 Hz); ^{13}C NMR ($CDCl_3$, 125.68 MHz) δ 128.3, 128.66, 128.70, 128.75, 128.79, 128.81, 129.0, 130.1, 130.4, 131.7, 133.7, 134.07, 134.09, 134.4, 135.0, 135.3, 135.6, 136.0, 136.3, 136.5, 142.0, 142.9, 151.1, 152.5, 183.4 (major), 185.2 (minor), 188.4 (major), 188.7 (minor), 192.3 (minor), 193.5 (major) (four carbons were obscured); MS *m*/*z* 346 (11), 330 (3), 253 (7), 247 (40), 111 (50), 105 (100); HRMS m/z ($[M+Na]^+$) calcd for $C_{21}H_{14}O_3SNa$, 369.0556, found 369.0557.

2-Benzoyl-4-phenyl-1-(thiophen-3-yl)but-2-ene-1,4-dione (4ao)

Isolated in 61% yield as a mixture of two stereoisomers (67 / 33), yellow oil: R_f = 0.28 (hexane / EtOAc = 5 / 1); ^1H NMR ($CDCl_3$, 399.82 MHz) δ 7.30 (1H, dd, *J*= 5.2, 2.8 Hz, minor), 7.38 (1H, dd, *J*= 5.2, 2.8 Hz, major), 7.43-7.65 (16H, m), 7.90 (2H, d, *J*= 6.8 Hz), 7.92 (2H, d, *J*= 8.0 Hz), 8.00 (2H, d, *J*= 8.0 Hz), 8.07 (2H, d, *J*= 8.2 Hz), 8.10 (1H, dd, *J*= 2.8, 1.2 Hz, minor), 8.34 (1H, dd, *J*= 3.0, 1.0 Hz, major); ^{13}C NMR ($CDCl_3$, 125.68 MHz) δ 126.66, 126.70, 127.0, 127.5, 128.59, 128.61, 128.71, 128.76, 128.9, 130.08, 130.16, 130.7, 133.6, 133.99, 134.00, 134.09, 134.5, 135.3, 135.6, 136.0, 136.4, 139.9, 141.0, 152.5, 153.5, 185.2 (major), 186.8 (minor), 188.4 (major), 188.7 (minor), 192.6 (minor), 193.9 (major) (three carbons were obscured); MS *m*/*z* 346 (15), 330 (8), 253 (11), 247 (15), 111 (54), 105 (100); HRMS m/z ($[M+Na]^+$) calcd for $C_{21}H_{14}O_3SNa$, 369.0556, found 369.0557.

2-Benzoyl-1-(1-naphthyl)-4-phenylbut-2-ene-1,4-dione (4ap)

Isolated in 52% yield as a mixture of two stereoisomers (69 / 31), yellow oil: R_f = 0.55 (hexane / EtOAc = 5 / 1); ^{1}H NMR ($CDCl_3$, 499.82 MHz) δ 7.38-7.65 (21H, m), 7.79-7.84 (4H, m), 7.93 (1H, d, *J*= 8.0 Hz), 7.97 (1H, d, *J*= 8.5 Hz), 8.01 (2H, d, *J*= 7.0 Hz), 8.05-8.13 (5H, m), 8.52 (1H, d, *J*= 9.0 Hz), 9.09 (1H, d, *J*= 8.5 Hz); ^{13}C NMR ($CDCl_3$, 125.68 MHz) δ 124.15, 124.22, 125.2, 126.1, 126.5, 126.8, 128.21, 128.23, 128.56, 128.59, 128.63, 128.65, 128.70, 128.72, 128.76, 128.82, 130.0, 130.14, 130.36, 130.62, 130.71, 131.6, 132.1, 132.6, 133.03, 133.35, 133.45, 133.57, 133.69, 133.74, 133.91, 134.0, 134.1, 134.3, 135.7, 136.03, 136.04, 136.1, 152.8, 152.9, 188.9 (major), 189.3 (minor), 193.2 (minor), 194.7 (major), 194.9 (minor), 195.1 (major); MS *m*/*z* 390 (12), 374 (10), 285 (14), 247 (96), 155 (74), 127 (100), 105 (96); HRMS m/z ($[M+Na]^+$) calcd for $C_{27}H_{18}O_3Na$, 413.1148, found 413.1153.

2-Benzoyl-1-(2-naphthyl)-4-phenylbut-2-ene-1,4-dione (4aq)

Isolated in 50% yield as a mixture of two stereoisomers (53 / 47), yellow oil: R_f = 0.36 and 0.30 (hexane / EtOAc = 5 : 1); ^{1}H NMR ($CDCl_3$, 499.82 MHz) δ 7.37-7.61 (18H, m), 7.80 (1H, d, *J*= 8.0 Hz), 7.84-7.91 (8H, m), 7.97 (1H, d, *J*= 8.0 Hz), 8.03-8.09 (6H, m), 8.50 (1H, s, major), 8.65 (1H, s, minor); ^{13}C NMR ($CDCl_3$, 125.68 MHz) δ 123.9, 124.5, 126.6, 127.1, 127.69, 127.75, 128.61, 128.62, 128.66, 128.67, 128.75, 128.78, 128.79, 129.1, 129.2, 129.7, 129.8, 130.2, 130.5, 130.8, 131.6, 132.2, 132.4, 132.8, 133.3, 133.4, 133.6, 134.01, 134.05, 135.5, 135.7, 135.82, 135.84, 136.11, 136.12, 152.8, 153.1, 188.5, 192.6, (minor) 192.8 (major), 193.8 (major), 193.9 (minor) (four carbons were obscured); MS *m*/*z* 390 (14), 374 (26), 297 (5), 247 (41), 155 (86), 105 (100); HRMS m/z ($[M+Na]^+$) calcd for $C_{27}H_{18}O_3Na$, 413.1148, found 413.1143.

3-Benzoyl-1,6-diphenylhex-2-ene-1,4-dione (4ar)

Isolated in 13% yield as a single stereoisomer, yellow oil: R_f = 0.35 (hexane / EtOAc = 5 / 1); ^{1}H NMR ($CDCl_3$, 499.82 MHz) δ 2.94-3.03 (4H, m) , 7.15 (2H, d, *J*= 6.5 Hz), 7.20 (1H, t, *J*= 7.2 Hz), 7.26-7.28 (2H, m), 7.44 (2H, t, *J*= 7.2 Hz), 7.48 (2H, t, *J*= 7.5 Hz), 7.58 (1H, t, *J*= 7.5 Hz), 7.61 (1H, t, *J*= 7.5 Hz), 7.86 (2H,

d, *J*= 6.0 Hz), 7.92 (2H, d, *J*= 8.5 Hz), 7.92 (1H, s); ^{13}C NMR ($CDCl_3$, 125.68 MHz) δ 29.5, 42.3, 126.3, 128.4, 128.50, 128.52, 128.80, 128.89, 128.92, 130.4, 133.9, 134.2, 135.7, 136.3, 140.2, 150.7, 188.8, 195.7, 196.5; MS *m*/*z* 368 (1), 352 (9), 263 (2), 133 (20), 105 (100); HRMS m/z ([M+Na]$^+$) calcd for $C_{25}H_{20}O_3Na$, 391.1305, found 391.1302.

2-Benzoyl-1,4-di-*p*-tolylbut-2-ene-1,4-dione (4ba)

Isolated in 48% yield (76% yield by use of $MgCl_2$) as a mixture of two stereoisomers (50 / 50), yellow oil: R_f = 0.35 (hexane / EtOAc = 5 / 1); ^{1}H NMR ($CDCl_3$, 499.82 MHz) δ 2.38 (3H, s, CH_3), 2.40 (6H, s, CH_3), 2.44 (3H, s, CH_3), 7.24 (6H, d, *J*= 8.5 Hz), 7.32 (2H, d, *J*= 7.5 Hz), 7.44 (2H, t, *J*= 7.5 Hz), 7.48 (2H, s, CH), 7.52 (2H, t, *J*= 7.5 Hz), 7.54 (1H, t, *J*= 7.5 Hz), 7.63 (1H, t, *J*= 7.5 Hz), 7.79 (4H, d, *J*= 8.0 Hz), 7.89 (2H, d, *J*= 7.5 Hz), 7.97 (2H, d, *J*= 8.0 Hz), 8.00 (2H, d, *J*= 8.0 Hz), 8.05 (2H, d, *J*= 7.5 Hz); ^{13}C NMR ($CDCl_3$, 125.68 MHz) δ 21.60, 21.63, 128.5, 128.6, 128.8, 128.9, 129.1, 129.28, 129.39, 129.436, 129.443, 130.1, 130.3, 130.4, 130.8, 132.9, 133.3, 133.4, 133.68, 133.70, 133.9, 135.5, 135.7, 144.4, 145.09, 145.11, 145.2, 152.2, 152.5, 187.9, 188.0, 192.3, 192.9, 193.4, 194.0 (three carbons were obscured); MS *m*/*z* 368 (16), 352 (22), 261 (21), 119 (100), 105 (33); HRMS m/z ([M+Na]$^+$) calcd for $C_{25}H_{20}O_3Na$, 391.1305; found 391.1308.

2-Benzoyl-1,4-bis(4-methoxyphenyl)but-2-ene-1,4-dione (4ca)

Isolated in 16% yield (25% yield by use of $MgCl_2$) as a mixture of two stereoisomers (50 / 50), yellow oil; R_f = 0.25 (hexane / AcOEt = 3 / 1); ^{1}H NMR ($CDCl_3$, 499.82 MHz) δ 3.82 (3H, s, OCH_3), 3.84 (6H, s, OCH_3), 3.87 (3H, s, OCH_3), 6.90 (6H, d, *J* = 8.5 Hz), 6.98 (2H, d, *J* = 9.0 Hz), 7.41-7.53 (7H, m), 7.62 (1H, t, *J* = 7.5 Hz), 7.87-7.89 (4H, m), 7.96 (2H, d, *J* = 9.0 Hz), 8.02 (2H, d, *J* = 7.5 Hz), 8.06 (4H, m); ^{13}C NMR ($CDCl_3$, 125.68 MHz) δ 55.4, 55.5, 55.6, 113.9, 114.0, 128.3, 128.6, 128.7, 129.1, 129.36, 129.39, 129.6, 130.2, 130.8, 131.2, 131,4, 132.8, 133.5, 133.9, 135.6, 135.8, 152.1, 153.2, 163.9, 164.2, 164.4, 186.6, 186.9, 191.1, 192.4, 193.1, 194.1 (six carbons were obscured); MS m/z 400 (13), 384 (51), 207 (69), 135 (100), 77 (43); HRMS m/z ([M + Na]$^+$) calcd for $C_{25}H_{20}O_5Na$ 423.1203, found 423.1205.

2-Benzoyl-1,4-bis(4-chlorophenyl)but-2-ene-1,4-dione (4da)

Isolated in 56% yield (74% yield by use of $MgCl_2$) as a mixture of two stereoisomers (53 / 47), yellow oil: R_f = 0.50 and 0.43 (hexane / AcOEt = 5 / 1); ^{1}H NMR ($CDCl_3$, 399.82 MHz) δ 7.38-7.55 (15H, m), 7.63 (1H, t, J = 7.2 Hz), 7.81 (4H, d, J = 8.4 Hz), 7.91 (2H, d, J = 8.8 Hz), 7.95-7.99 (4H, m), 8.02 (2H, d, J = 8.0 Hz); ^{13}C NMR ($CDCl_3$, 100.54 MHz) δ 128.7, 128.8, 128.93, 128.96, 129.08, 129.13, 129.16, 129.9, 130.0, 130.1, 130.3, 131.5, 133.5, 133.8, 134.0, 134.2, 135.1, 135.4, 140.1, 140.6, 140.7, 140.8, 152.7, 153.0, 187.1, 191.3 (minor), 192.4 (major), 192.5 (major), 193.4 (minor) (five carbons were obscured); MS m/z 408 (15), 315 (10), 281 (32), 139 (100), 105 (96); HRMS m/z ([M + Na]$^+$) calcd for $C_{23}H_{14}Cl_2O_3Na$ 431.0212, found 431.0214.

2-Benzoyl-1,4-bis(4-bromophenyl)but-2-ene-1,4-dione (4ea)

Isolated in 42% yield (63% yield by use of $MgCl_2$) as a mixture of two stereoisomers (58 / 42), yellow oil: R_f (hexane : AcOEt = 5 / 1) = 0.45 and 0.40; ^{1}H NMR ($CDCl_3$, 399.82 MHz) δ 7.41-7.58 (13H, m), 7.62-7.66 (3H, m), 7.73 (4H, d, J = 8.4 Hz), 7.84 (2H, d, J = 8.8 Hz), 7.90 (2H, d, J = 8.4 Hz), 7.95 (2H, d, J = 7.8 Hz), 8.02 (2H, d, J = 8.0 Hz); ^{13}C NMR ($CDCl_3$, 125.68 MHz) δ 128.7, 128.8, 128.97, 129.03, 129.58, 129.67, 129.70, 129.8, 130.1, 130.2, 130.4, 131.6, 132.0, 132.13, 132.18, 132.21, 133.9, 134.0, 134.3, 134.4, 134.66, 134.70, 135.2, 135.4, 152.8, 153.1, 187.4, 191.5 (minor), 192.4 (major), 192.8 (major), 193.3 (minor) (three carbons were obscured); MS m/z 495 (21), 405 (15), 325 (33), 183 (75), 105 (100); HRMS m/z ([M + Na]$^+$) calcd for $C_{23}H_{14}Br_2O_3Na$ 518.9202, found 518.9205.

III. General procedure for the reaction of cyclic 1,2-diketones 5 with enone 6.

A Schlenk tube was charged with thiazolium salt **3** (37 mg, 0.10 mmol), cyclohexane-1,2-dione (**5a**) (56 mg, 0.50 mmol), phenyl vinyl ketone (**6a**) (66 mg, 0.50 mmol), DMF (1.0 mL), and then iPr_2NH (14 μL, 0.10 mmol) under N_2. The mixture was stirred at room temperature for 24 h. After quenching with water, accurately weighted diphenylmethane (35.0 mg, 208 mmol) was added to the

mixture as an internal standard. The organic layer was extracted with ether, washed with brine, dried over $MgSO_4$, and concentrated under vacuum. The residue was measured by ^{1}H NMR to determine the yield of **7a** (49% yield) and bicyclo[3.2.1]octanone **8** (*ca.*10% yield). Then, the crude products were purified by column chromatography on silica gel with hexane-EtOAc eluent to give 55 mg (45% yield) of **7a** and ca. 12 mg (10% yield) of **8**.

2-Benzoylcyclooctane-1,4-dione (7a)

Isolated in 45% yield as a yellow oil; R_f = 0.38 (hexane : EtOAc = 3 / 1); ^{1}H NMR ($CDCl_3$, 499.82 MHz) δ 1.63-1.87 (2H, m), 1.98-2.06 (2H, m), 2.37-2.41 (1H, m), 2.56-2.65 (3H, m), 2.68 (1H, dd, *J* = 12.0, 4.5 Hz), 3.45 (1H, t, *J* = 12.0 Hz), 4.86 (1H, dd, *J* = 12.0, 4.5 Hz), 7.46 (2H, t, *J* =7.5 Hz), 7.56 (1H, t, *J* = 7.5 Hz), 7.85 (2H, d, *J* = 7.5 Hz); ^{13}C NMR ($CDCl_3$, 100.53 MHz) δ 24.5, 25.4, 40.9, 42.3, 42.5, 59.9, 128.3, 128.9, 133.7, 135.7, 193.4, 209.4, 213.5; MS m/z 244 (27), 133 (26), 105 (100); HRMS m/z ($[M + Na]^+$) calcd for $C_{15}H_{16}O_3Na$, 267.0992, found 267.0994.

2-Benzoylcyclononane-1,4-dione (7b)

Isolated in 24% yield as a white solid; mp 107-108 °C; R_f = 0.20 (hexane / EtOAc = 5 / 1); ^{1}H NMR ($CDCl_3$, 399.82 MHz) δ 1.55-1.58 (1H, m), 1.67-1.69 (1H, m), 1.77-1.86 (4H, m), 2.31-2.37 (1H, m), 2.49-2.55 (1H, m), 2.63-2.71 (2H, m), 2.74 (1H, dd, *J*= 10.8, 2.4 Hz, CHC*H*H), 3.32 (1H, dd, *J*= 10.8, 9.2 Hz, CHCH*H*), 5.00 (1H, dd, *J*= 9.2, 2.4 Hz, C*H*CHH), 7.46 (2H, t, *J*= 6.0 Hz), 7.57 (1H, t, 6.0 Hz), 7.86-7.88 (2H, m,); ^{13}C NMR ($CDCl_3$, 100.54 MHz) δ 21.3, 22.3, 28.2, 40.7, 43.2, 46.4, 58.4, 128.3, 128.8, 133.6, 135.9, 193.7, 209.7, 214.6; MS m/z 258 (17), 133 (14), 105 (100); HRMS m/z ($[M+Na]^+$) calcd for $C_{16}H_{18}O_3Na$, 281.1154; found 281.1148.

2-Benzoylcyclotetradecane-1,4-dione (7c)

Isolated in 43% yield as a white solid; mp 90-91 °C; R_f = 0.15 (hexane / EtOAc = 5 / 1); ^{1}H NMR ($CDCl_3$, 399.82 MHz) δ 1.14-1.40 (13H, m), 1.48-1.56 (1H, m), 1.70-1.79 (1H, m), 1.84-1.94 (1H, m), 2.37-2.43 (1H, m), 2.53 (2H, t, *J*= 6.0 Hz),

2.65-2.73 (1H, m), 2.83 (1H, dd, *J*= 18.0, 3.6 Hz, CHC*H*H), 3.47 (1H, dd, 18.0, 9.6 Hz, CHCH*H*), 5.27 (1H, dd, *J*= 9.6, 3.6 Hz, C*H*CHH), 7.49 (2H, t, *J*= 7.6 Hz), 7.59 (1H, t, 7.2 Hz), 8.00-8.02 (2H, m); ^{13}C NMR ($CDCl_3$, 125.68 MHz) δ 21.3, 22.5, 25.3, 25.6, 25.7, 26.2, 26.8, 40.8, 41.5, 41.8, 56.4, 128.8, 128.9, 133.7, 135.7, 195.4, 205.6, 209.0 (one carbone was obscured); MS m/z 328 (41), 133 (44), 105 (100); HRMS m/z ($[M+Na]^+$) calcd for $C_{21}H_{18}O_3Na$, 351.1936; found 351.1928.

7-Benzoyl-1-hydroxybicyclo[3.2.1]octan-8-one (8)

Two stereoisomers were obtained in ca. 10% (6/4) combined yield. Major isomer: yellow solid, mp 100-102 °C; R_f = 0.23 (hexane / AcOEt = 3 / 1); ^{1}H NMR ($CDCl_3$, 399.82 MHz) δ 1.80 (1H, m), 1.87-2.04 (5H, m), 2.31-2.37 (2H, m), 2.62 (1H, m), 3.26 (1H, br s), 4.07 (1H, dd, *J* = 10.4, 4.4 Hz), 7.48 (2H, t, *J* =7.6 Hz), 7.58 (1H, t, *J* = 7.2 Hz), 7.94 (2H, d, *J* = 7.8 Hz); ^{13}C NMR ($CDCl_3$, 100.53 MHz) δ 18.2, 25.9, 35.4, 41.9, 43.1, 45.1, 79.7, 128.4, 128.6, 133.4, 136.8, 202.1, 216.7; HRMS: m/z ($[M+Na]^+$) calcd for $C_{15}H_{16}O_3Na$, 267.0992, found 267.0991. Minor isomer: brown solid; R_f = 0.33 (Hexane / AcOEt = 3 / 1); ^{1}H NMR ($CDCl_3$, 399.82 MHz) δ 1.47 (1H, dt, *J* = 14.4, 6.4 Hz), 1.62 (1H, td, *J* = 13.2, 6.4 Hz), 1.93 (1H, td, *J* = 14.0, 5.1 Hz), 2.03-2.14 (3H, m), 2.25 (1H, m), 2.62-2.69 (2H, m), 3.23 (1H, br s), 3.84 (1H, dd, *J* = 11.6, 7.2 Hz), 7.47 (2H, t, *J* =7.6 Hz), 7.58 (1H, t, *J* = 7.4 Hz), 8.11 (2H, d, *J* = 8.4 Hz); ^{13}C NMR ($CDCl_3$, 100.53 MHz) δ 16.9, 22.3, 35.7, 40.6, 41.8, 47.2, 79.6, 128.3, 129.3, 133.4, 137.3, 198.7, 218.1.

IV. Synthesis of (*E*)-1-(3-Mesityl-3,4,5,6,7,8-hexahydro-2*H*-cyclohepta [*d*]thiazol-2-ylidene)-2-oxo-2-phenylethyl benzoate (10)

Thiazolium salt **3** (744 mg, 2.0 mmol) and 1,3-diphenylpropane-1,2,3-trione (**9**) (476 mg, 2.0 mmol) were dissolved in DMF (4.0 mL) under argon. After addition of DIPEA (341 μL, 2.0 mmol), the mixture was stirred at room temperature for 12 h. The reaction was quenched with water and then the organic layer was extracted with dichloromethane, washed with brine, dried over $MgSO_4$, and concentrated under vacuum. The crude product was purified by column chromatography on silica gel to give the bisacylated Breslow intermediates **10** (845 mg, 83% yield).

10: yellow solid; mp 196-197 °C (from EtOAc / hexane): R_f = 0.20 (EtOAc / hexane = 1 / 1); ^{1}H NMR ($CDCl_3$, 499.82 MHz) δ 1.48-1.52 (2H, m), 1.71 (3H, s, CH_3), 1.75-1.79 (4H, m), 1.86 (3H, s, CH_3), 2.01-2.04 (2H, m), 2.14 (3H, s, CH_3), 2.67-2.70 (2H, m), 6.36 (1H, s), 6.63 (1H, s), 7.12-7.14 (3H, m), 7.22 (2H, t, *J*= 7.5 Hz), 7.41 (1H, t, *J*= 7.5 Hz), 7.48 (2H, d, *J*= 7.5 Hz), 7.60-7.62 (2H, m); ^{13}C NMR ($CDCl_3$, 100.54 MHz) δ 17.3, 17.9, 20.5, 26.3, 26.4, 26.6, 27.4, 31.1, 118.0, 120.2, 127.4, 127.5, 127.6, 128.7, 128.8, 128.99, 129.02, 129.5, 132.5, 134.3, 135.5, 135.8, 137.6, 138.7, 139.2, 152.0, 164.9, 180.2; HRMS m/z ($[M+Na]^+$) calcd for $C_{32}H_{31}NO_3SNa$, 532.1922; found 532.1907.

V. Synthesis of 2-(((4-chlorobenzoyl)oxy)(4-chlorophenyl)methyl)-3-mesityl-5,6,7,8-tetrahydro-4*H*-cyclohepta[*d*]thiazol-3-ium perchlorate (12)

Thiazolium salt **3** (744 mg, 2.0 mmol) and 1,2-bis(4-chlorophenyl)ethane-1,2-dione (**1d**, 557 mg, 2.0 mmol) were dissolved in DCM (8.0 mL) under argon. After addition of DIPEA (341 μL, 2.0 mmol), the mixture was stirred at room temperature for 12 h. Then, all volatile compounds were evaporated under vacuum without aqueous treatment. The residue was directly chromatographed on silica gel with EtOAc eluent to afford **12** (1.20 g, 92% yield).

12: white solid; mp 115-120 °C: R_f = 0.10 (EtOAc); ^{1}H NMR ($CDCl_3$, 499.82 MHz) δ 1.30 (3H, s, CH_3), 1.54-1.70 (2H, m), 1.79-1.99 (4H, m), 2.10 (3H, s, CH_3), 2.40 (3H, s, CH_3), 2.42-2.54 (2H, m), 3.14-3.29 (2H, m), 6.68 (1H, s, OCH), 6.92 (1H, s), 7.11 (1H, s), 7.16-7.18 (2H, m), 7.33-7.36 (2H, m), 7.47-7.49 (2H, m), 8.00-8.03 (2H, m); ^{13}C NMR ($CDCl_3$, 100.54 MHz) δ 16.5, 17.3, 21.1, 25.3, 26.2, 26.9, 28.0, 30.4, 71.5, 125.7, 129.1, 129.5, 130.0, 130.1, 130.28, 130.32, 130.7, 131.5, 133.5, 134.8, 136.8, 140.2, 140.9, 142.8, 149.3, 163.1, 167.0; MS m/z 239 (100), 140 (32), 113 (9), 110 (28); Elemental Analysis calcd for $C_{31}H_{30}Cl_3NO_6S$: C, 57.20; H, 4.65; N, 2.15; found C, 56.85; H, 4.59; N, 2.10.

VI. Reaction of 1,2-bis(4-chlorophenyl)ethane-1,2-dione (1d) with phenyl vinyl ketone (6a) catalyzed by 12 and DIPEA

Benzil **1d** (140 mg, 0.50 mmol), enone **6a** (66 mg, 0.50 mmol), and the salt **12** (65 mg, 0.10 mmol) were dissolved in DMF (4.0 mL) under argon. The reaction

was started by addition of DIPEA (17 μL, 0.1 mmol) and the stirring was continued for 16 h at 50 °C. After quenching with water, accurately weighted diphenylmethane (35.0 mg, 208 mmol) was added to the mixture as an internal standard. The organic layer was extracted with ether, washed with brine, dried over $MgSO_4$, and concentrated under vacuum. The residue was measured by ^{1}H NMR to determine the yield of **13** (85% yield). The crude product **13** was purified by column chromatography on silica gel.

2-Benzoyl-1,4-bis(4-chlorophenyl)butane-1,4-dione (**13**) [3] :Isolated as a yellow solid (169 mg, 82%): mp 151-153ºC; R_f = 0.25 (hexane / AcOEt = 10 / 1) ; ^{1}H NMR ($CDCl_3$, 399.82 MHz) δ 3.66 (1H, dd, *J* = 18.0, 5.6 Hz, C*H*H), 3.80 (1H, dd, *J* = 18.0, 6.8 Hz, CH*H*), 6.04 (1H, br t, *J* = 6.4 Hz, CH), 7.42 (2H, d, *J* = 8.4 Hz), 7.44, (2H, d, *J* = 8.4 Hz), 7.45 (2H, t, *J* = 7.2 Hz), 7.60 (1H, t, *J* = 7.2 Hz), 7.93-7.95 (4H, m), 8.00 (2H, d, *J* = 7.2 Hz); ^{13}C NMR ($CDCl_3$, 100.53 MHz) δ 37.9, 51.4, 128.6, 129.01,129.04, 129.2, 129.7, 130.0, 133.9, 134.0, 134.3, 135.2, 140.1, 140.2, 194.3, 195.4, 195.5; MS *m/z* 392 (100), 207 (43), 179 (25), 139 (38), 105 (93); HRMS *m/z* ($[M + Na]^+$) calcd for $C_{23}H_{16}Cl_2O_3Na$ 433.0369, found 433.0370.

VII. References

1. The *E*/*Z* ratio of **4** could be estimated by the signal intensity (height) of well recognizable three carbonyl carbons in ^{13}C NMR spectra. For example, the mixture of **4ai** showed three pairs of signals [188.28 (lower) and 188.30 (higher), 191.6 (lower) and 192.6 (higher), 192.8 (higher) and 193.6 (lower)]. Average of the three ratios was 40/60. The ratio was also determinable by ^{1}H NMR integration of methyl signals in **4ab**-**4ad**, **4af**, and **4ak**.
2. Gao, M.; Yang, Y. ; Wu, Y.-D.; Deng, C.; Cao, L.-P.; Meng, X.-G.; Wu, A.-X. *Org. Lett.*, **2010**, *12*, 1856-1859. doi:10.1021/ol100473f
3. Takaki, K.; Ohno, A.; Hino, M.; Shitaoka, T.; Komeyama, K.; Yoshida, H. *Chem. Commun.* **2014**, *50*, 12285-12288. doi:10.1039/c4cc05436a

VIII. Copies of ^1H and ^{13}C NMR spectra of the products

2-Benzoyl-1,4-diphenylbut-2-ene-1,4-dione (4aa)

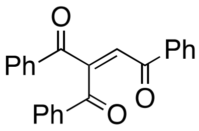

4aa

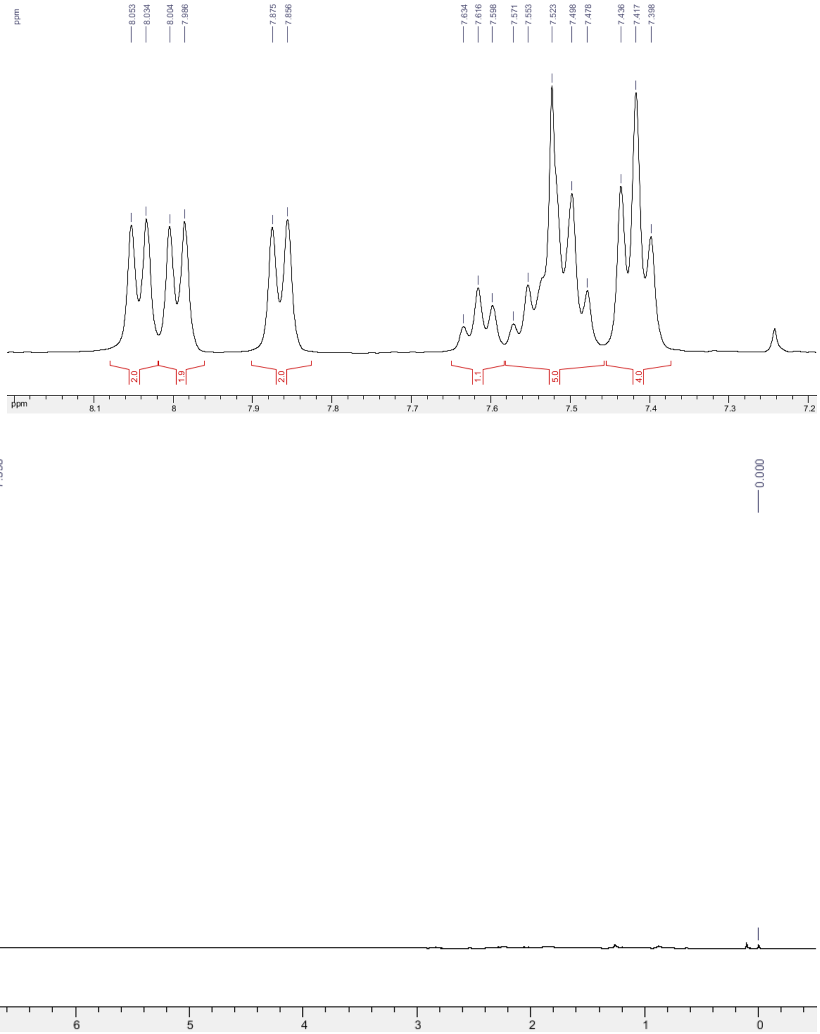

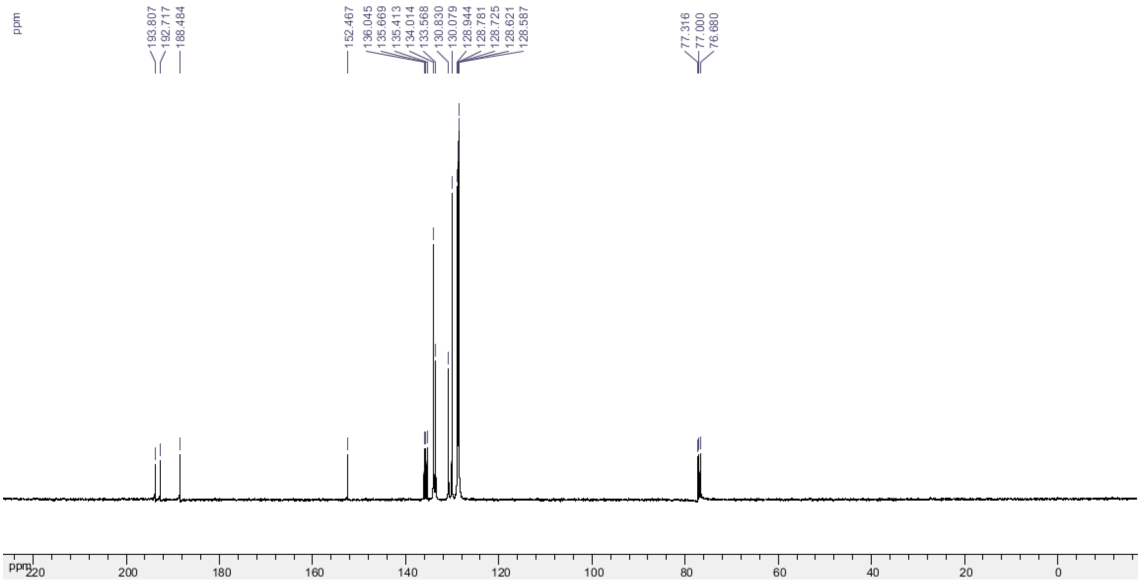

2-Benzoyl-4-phenyl-1-(*o*-tolyl)but-2-ene-1,4-dione (4ab)

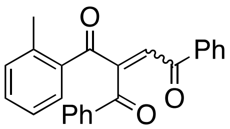

4ab

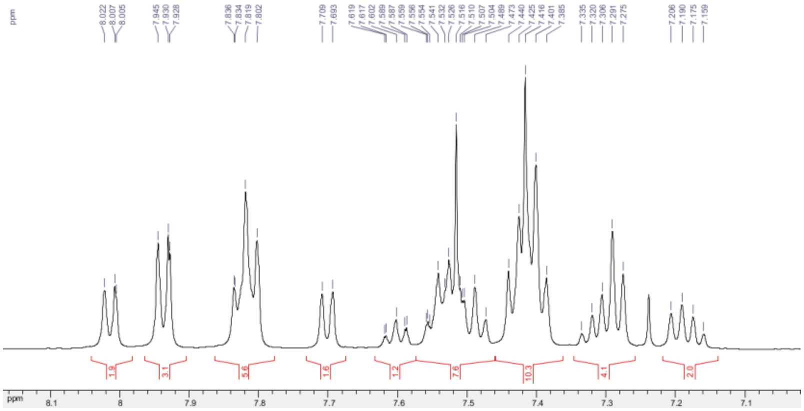

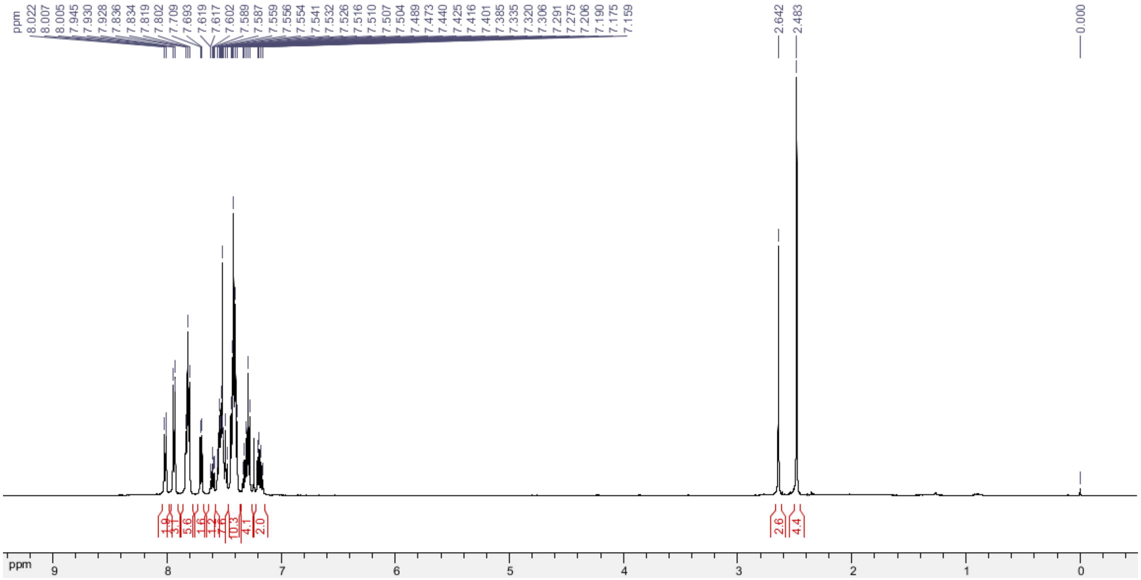

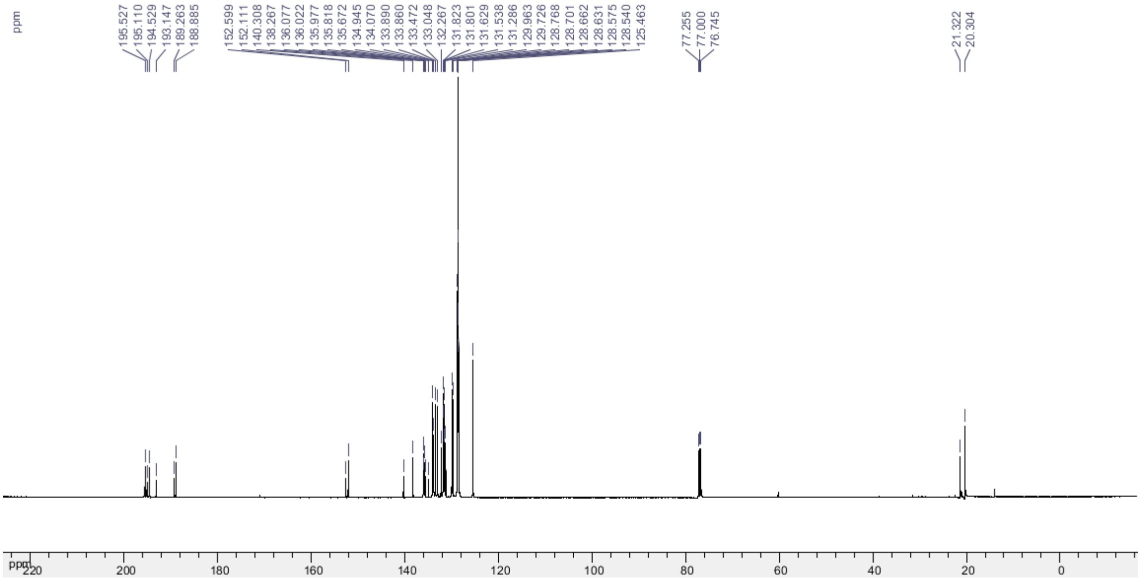

2-Benzoyl-4-phenyl-1-(*m*-tolyl)but-2-ene-1,4-dione (4ac)

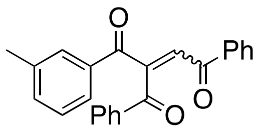

4ac

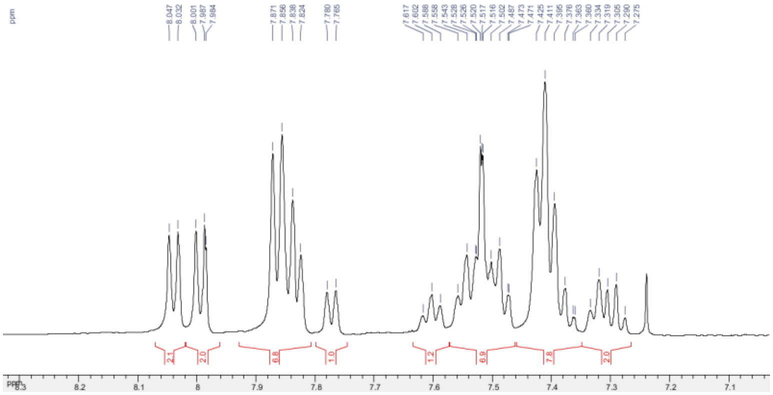

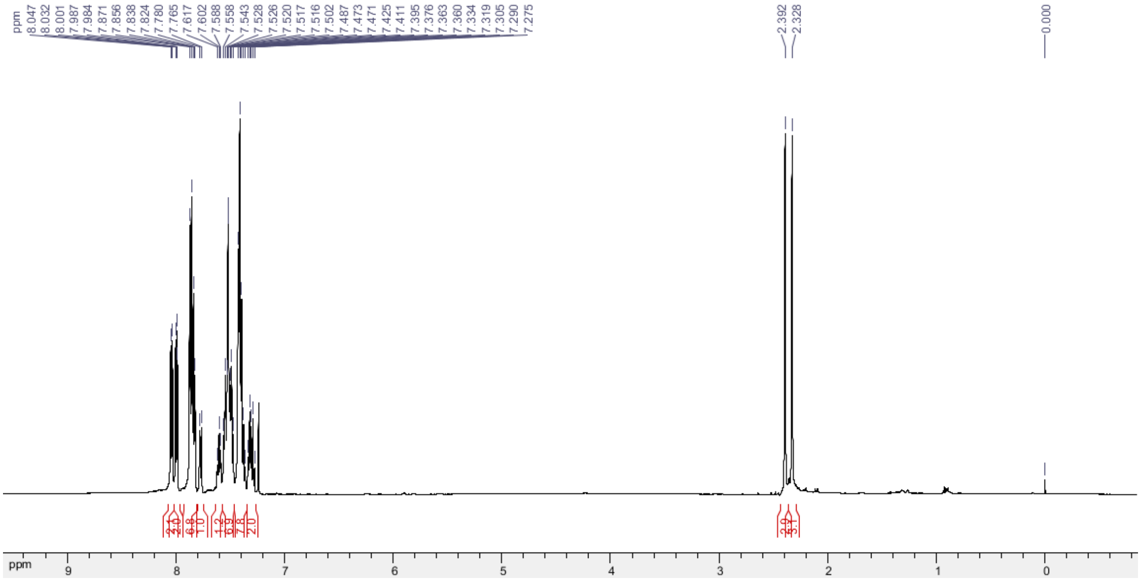

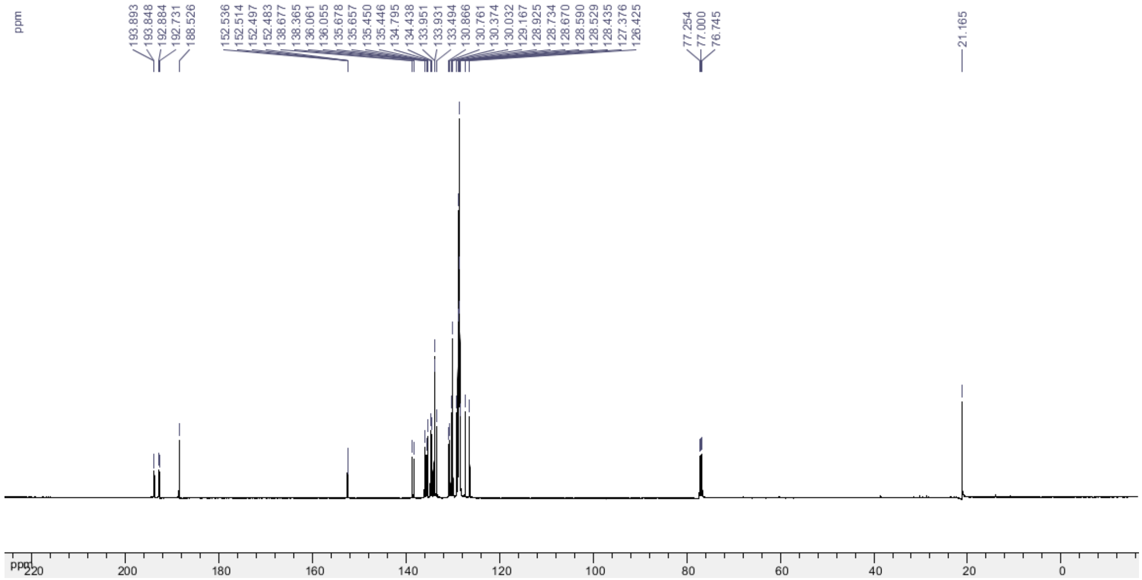

2-Benzoyl-4-phenyl-1-(p-tolyl)but-2-ene-1,4-dione (4ad)

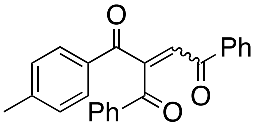

4ad

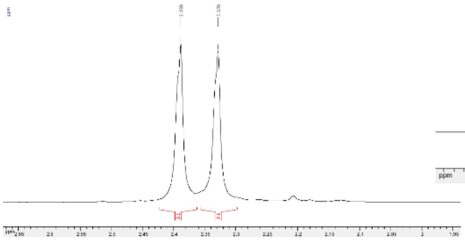

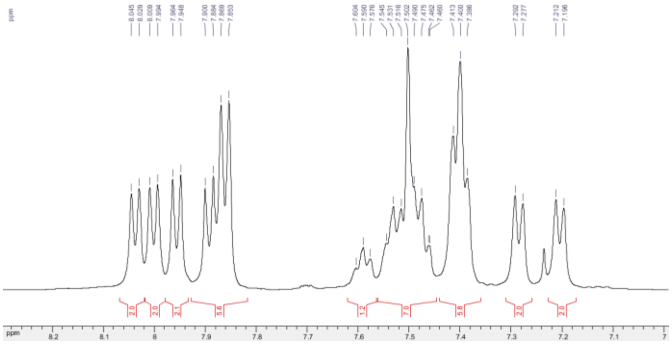

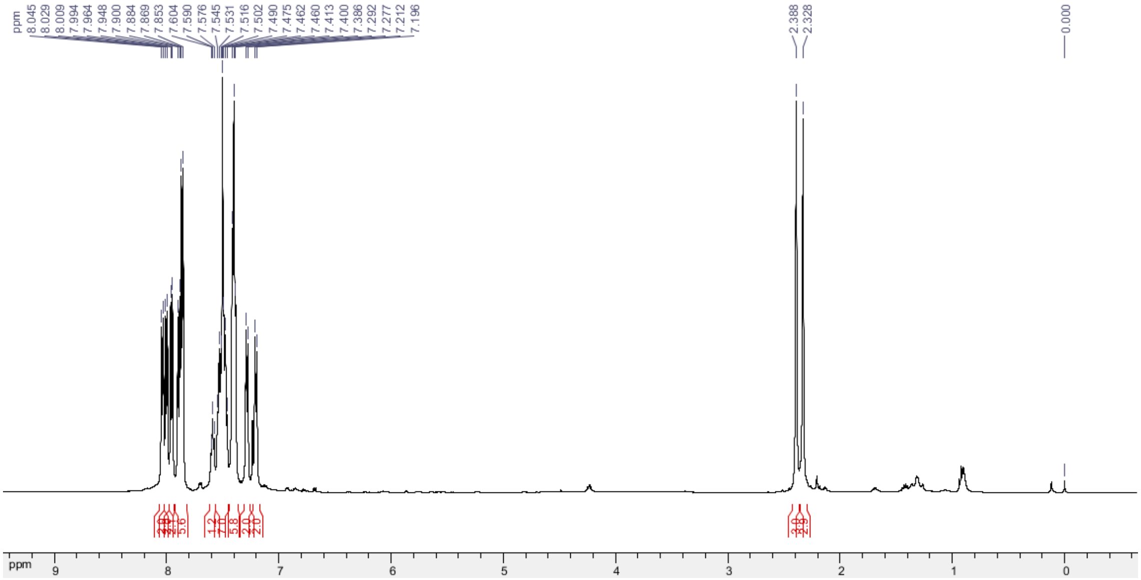

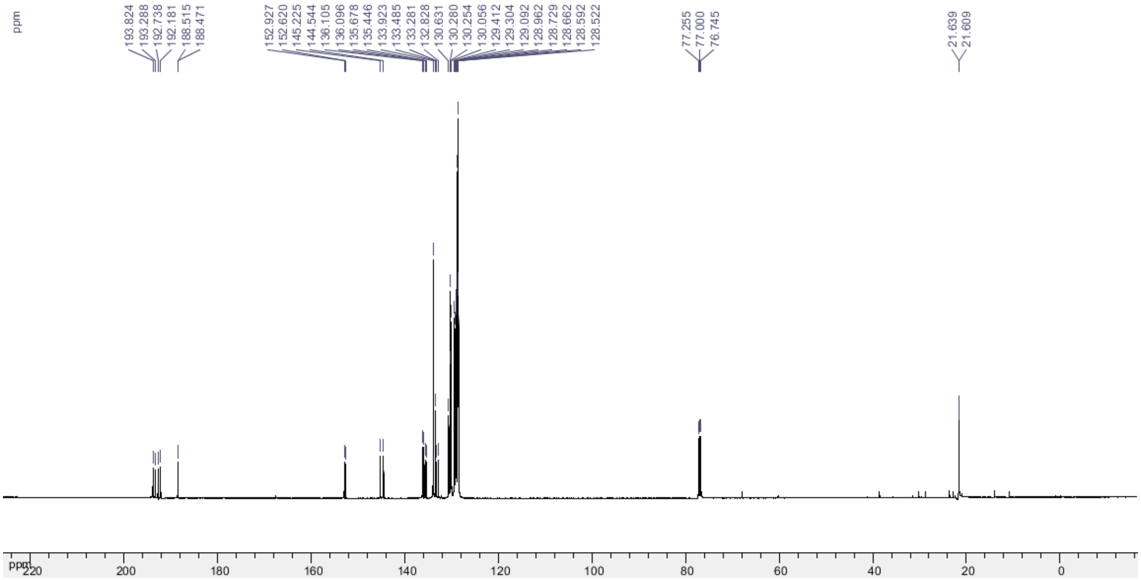

2-Benzoyl-1-mesityl-4-phenylbut-2-ene-1,4-dione (4ae)

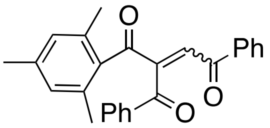

4ae

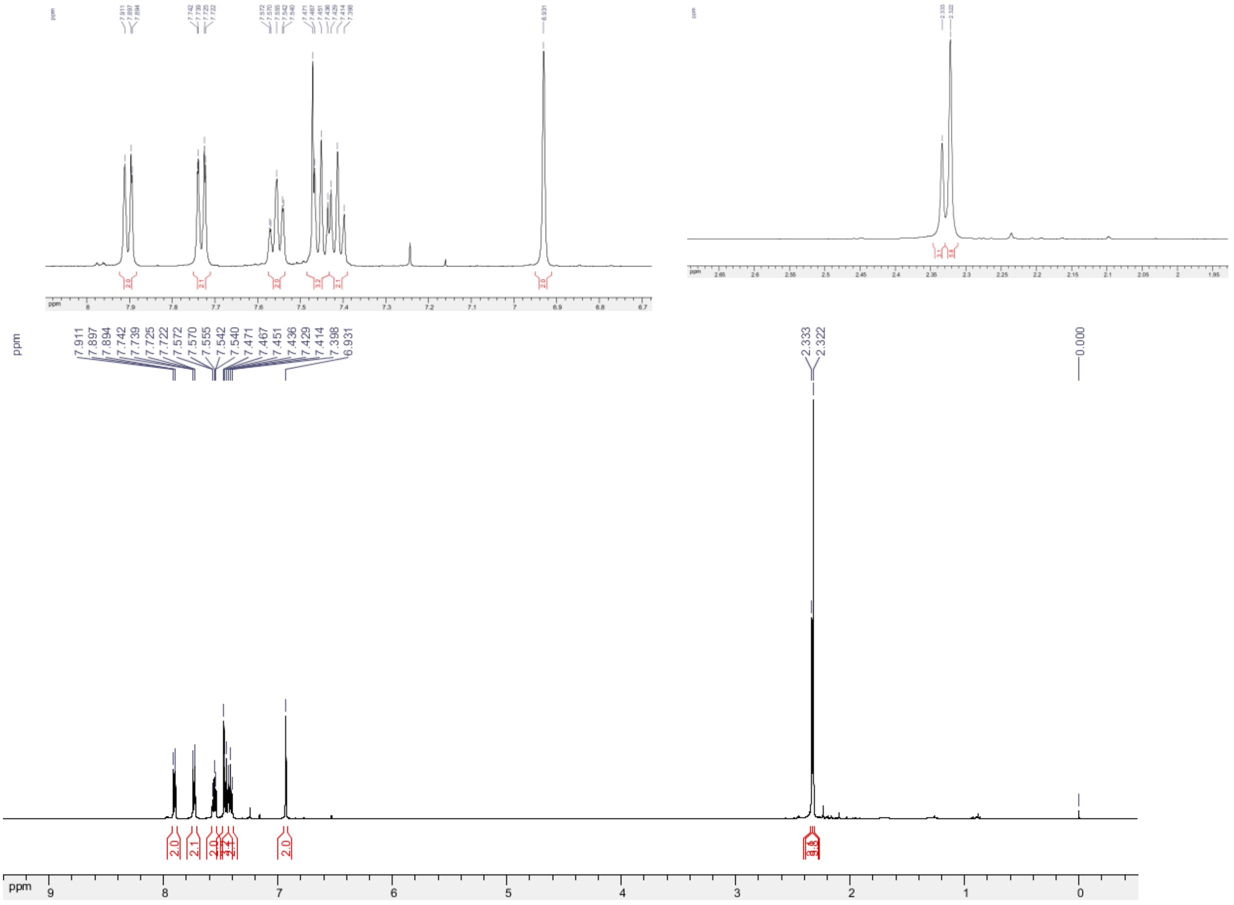

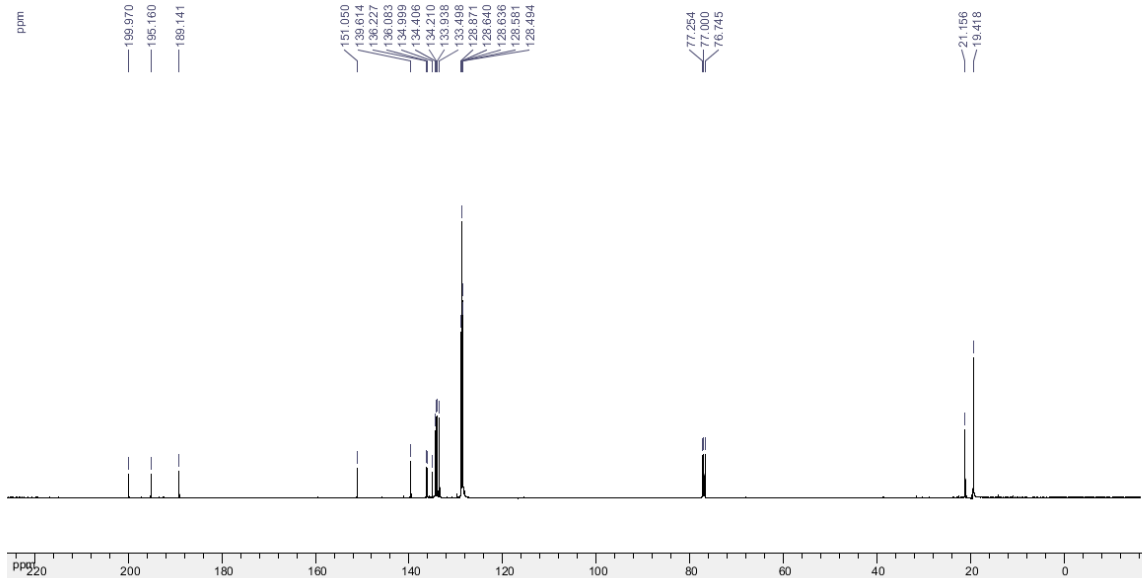

2-Benzoyl-1-(4-methoxyphenyl)-4-phenylbut-2-ene-1,4-dione (4af)

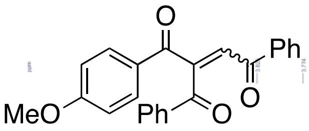

4af

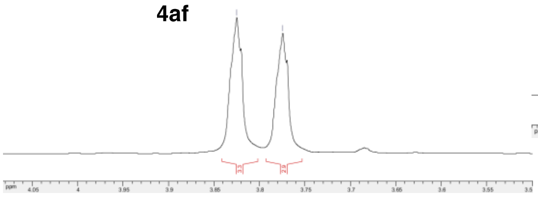

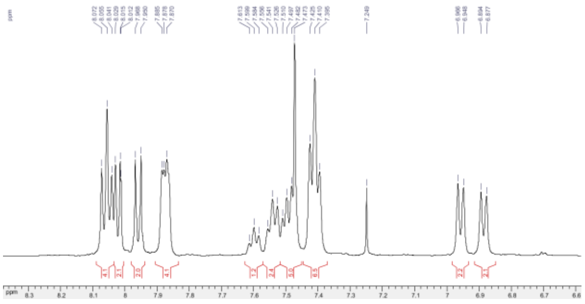

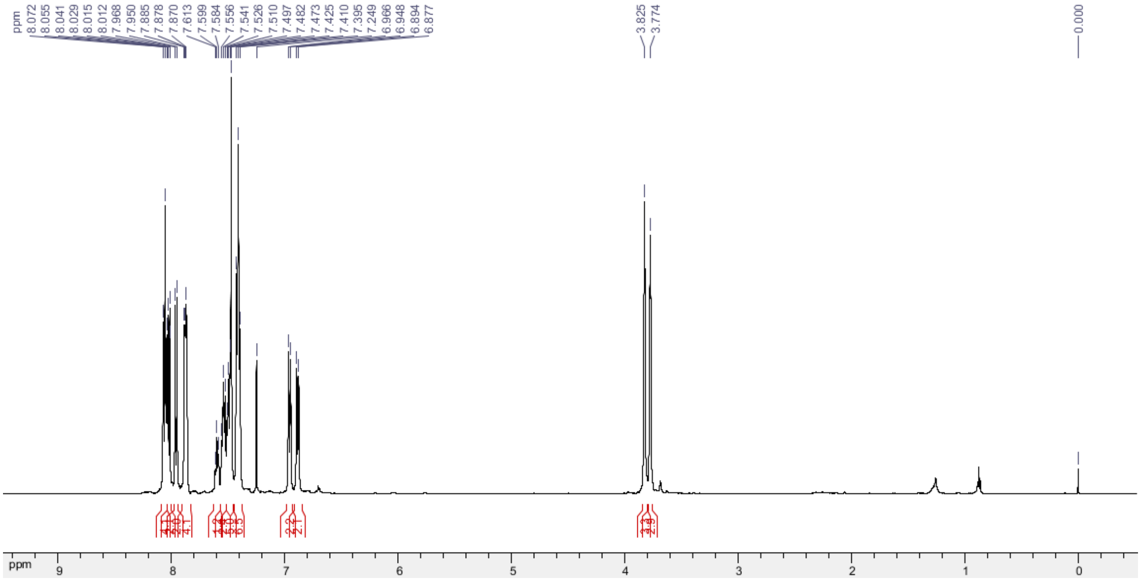

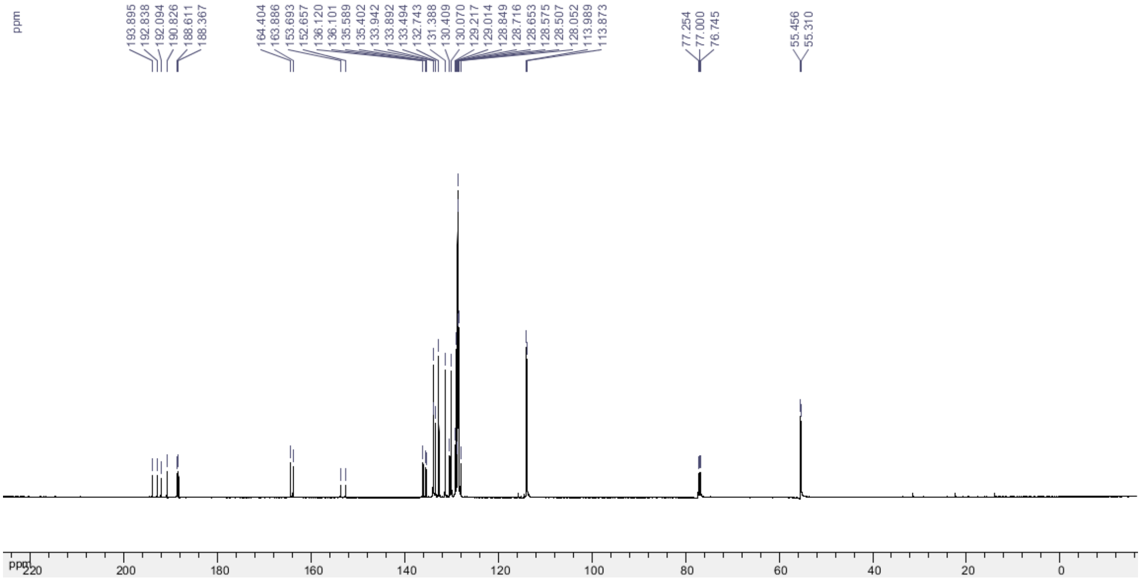

2-Benzoyl-1-(2-chlorophenyl)-4-phenylbut-2-ene-1,4-dione (4ag)

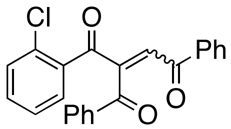

4ag

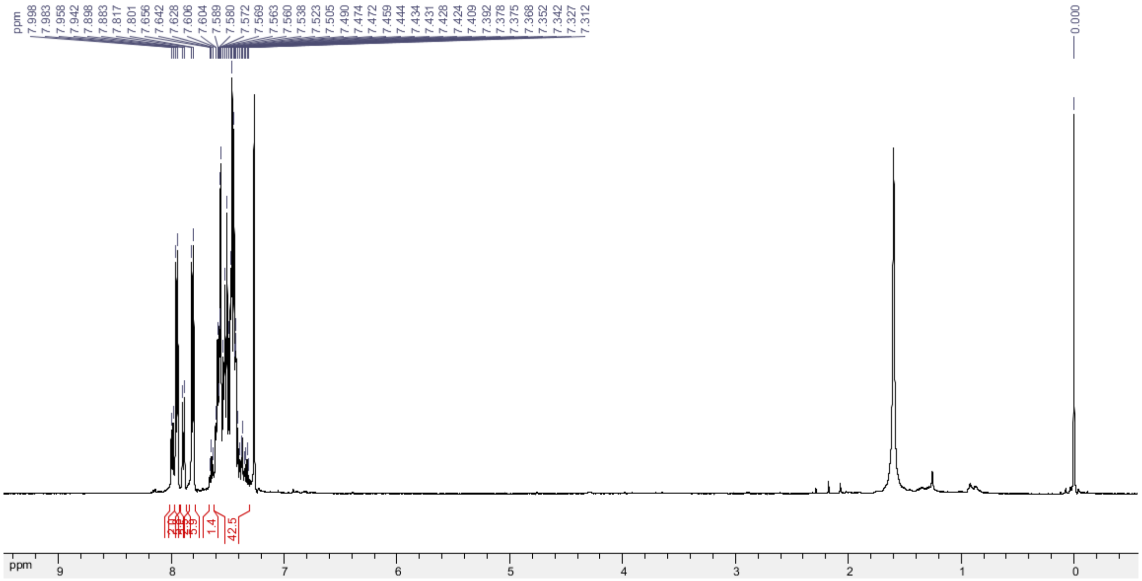

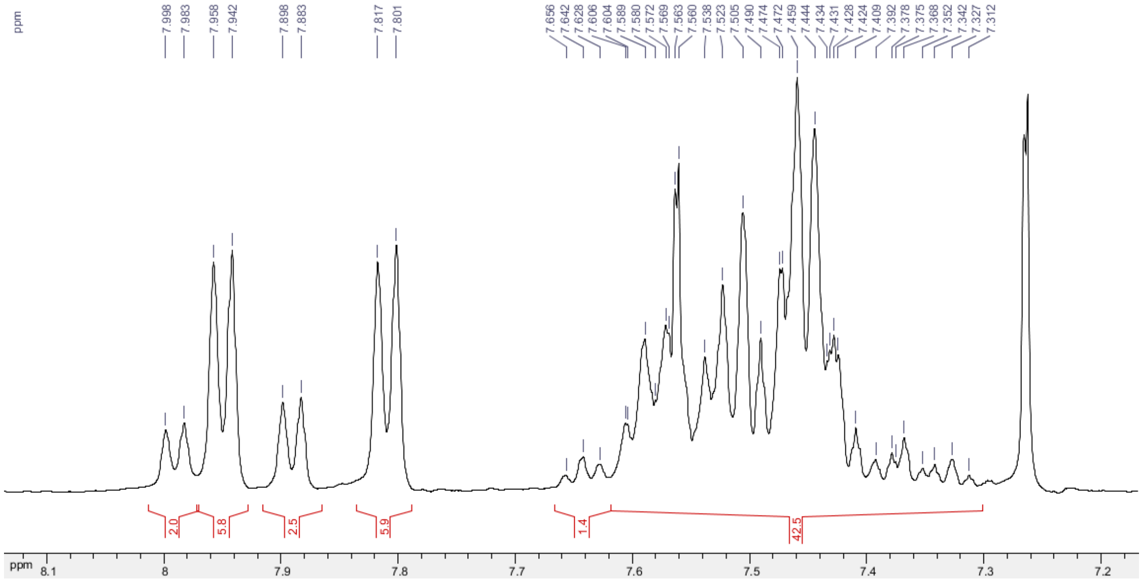

2-Benzoyl-1-(2-chlorophenyl)-4-phenylbut-2-ene-1,4-dione (4ag)

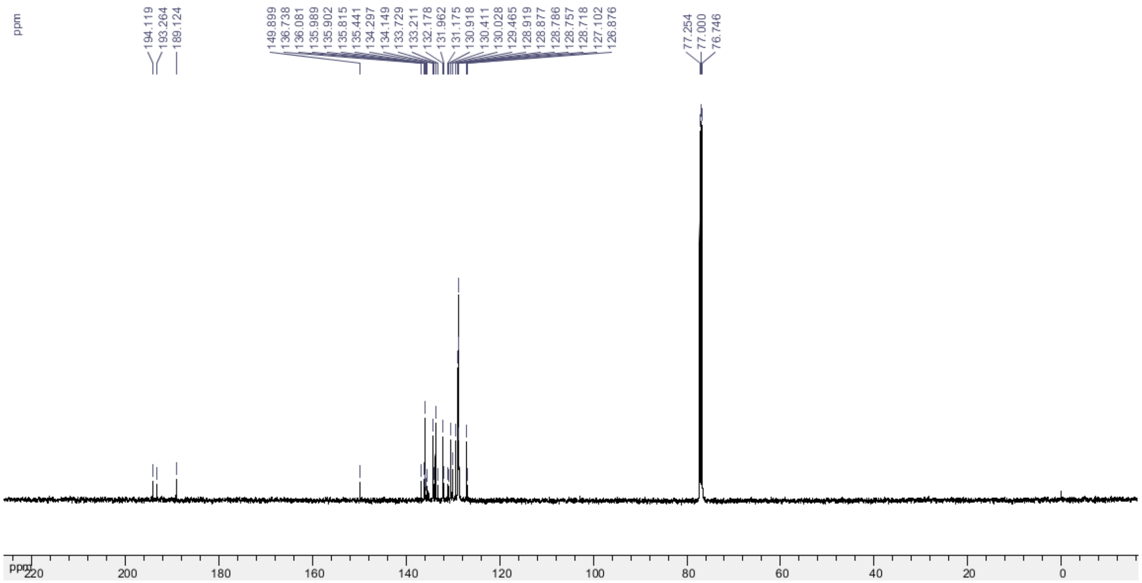

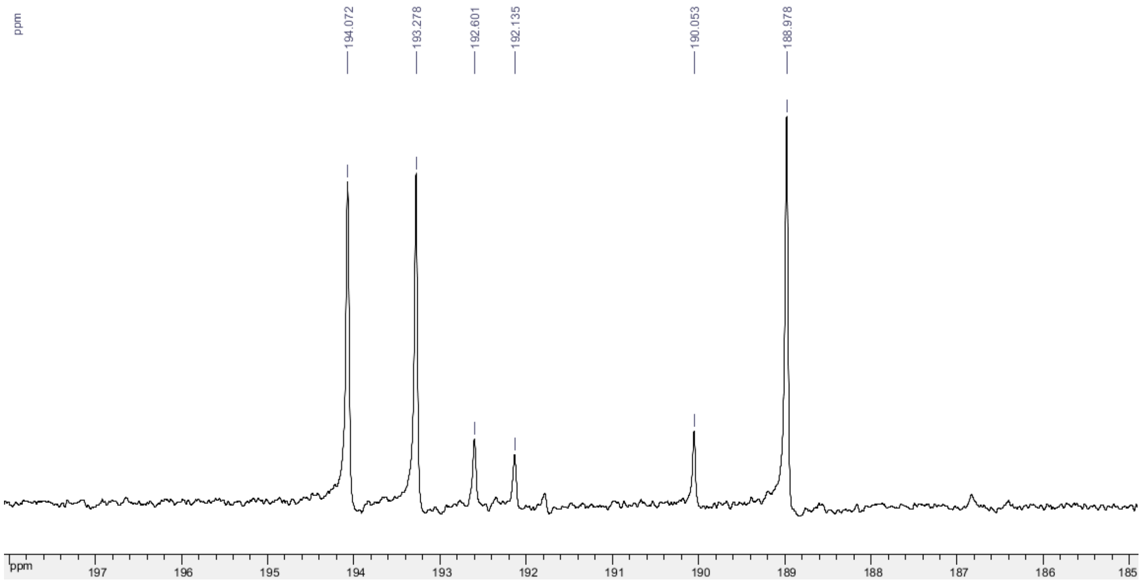

2-Benzoyl-1-(3-chlorophenyl)-4-phenylbut-2-ene-1,4-dione (4ah)

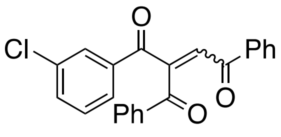

4ah

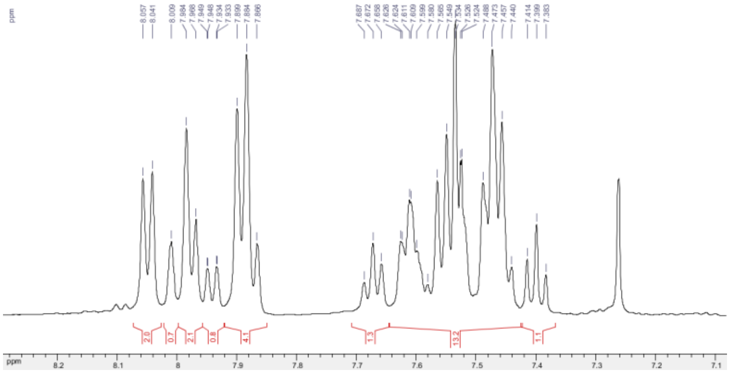

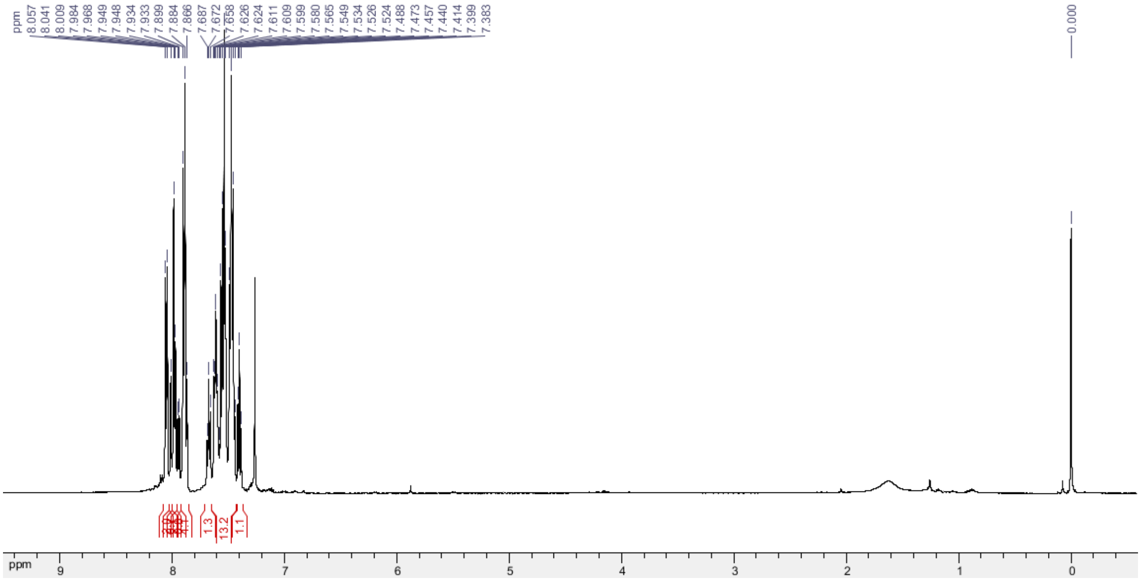

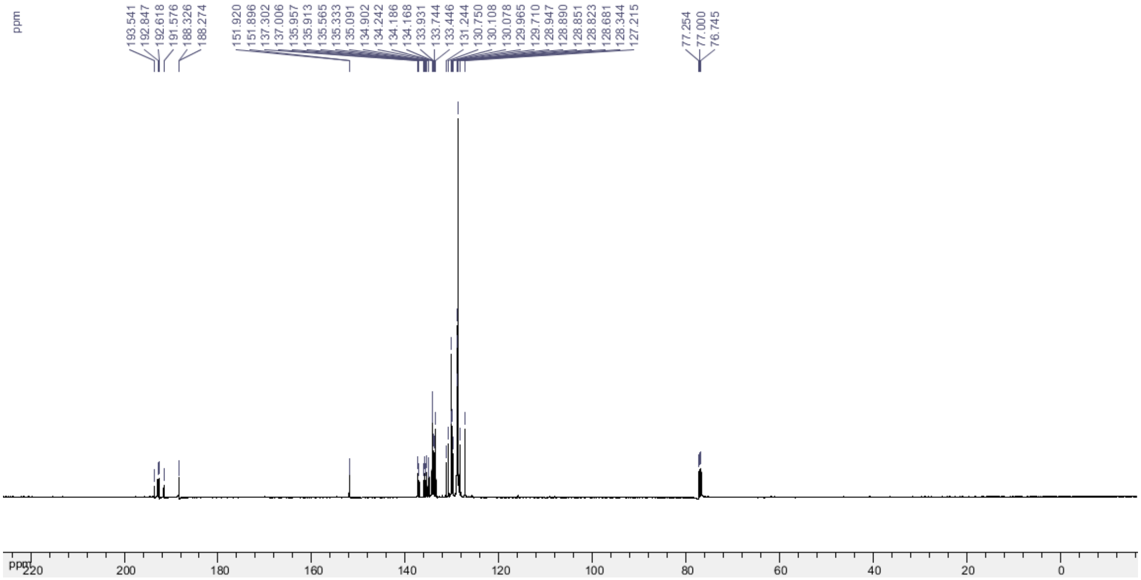

2-Benzoyl-1-(4-chlorophenyl)-4-phenylbut-2-ene-1,4-dione (4ai)

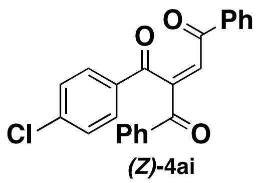

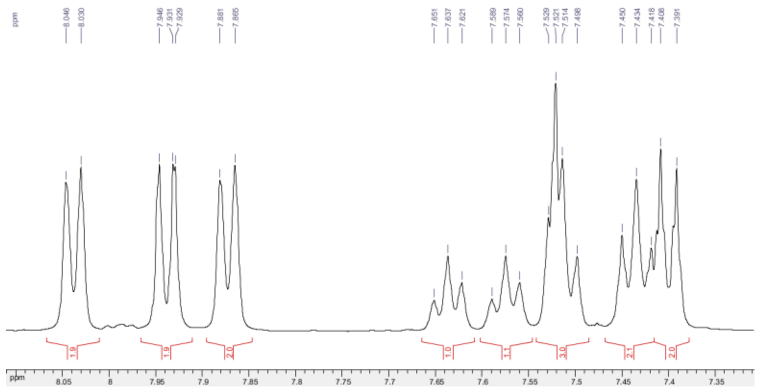

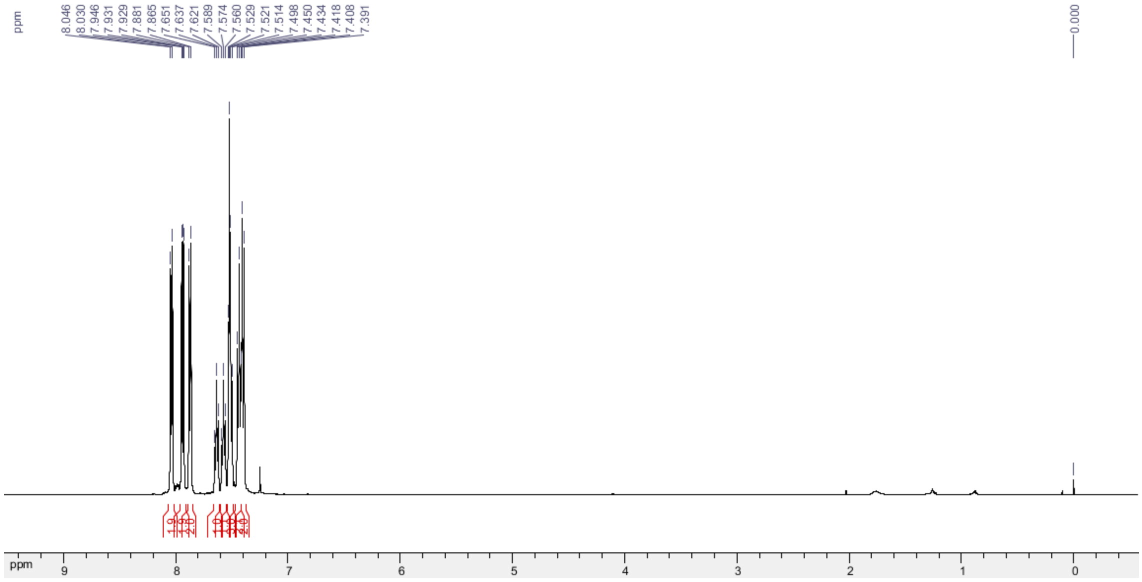

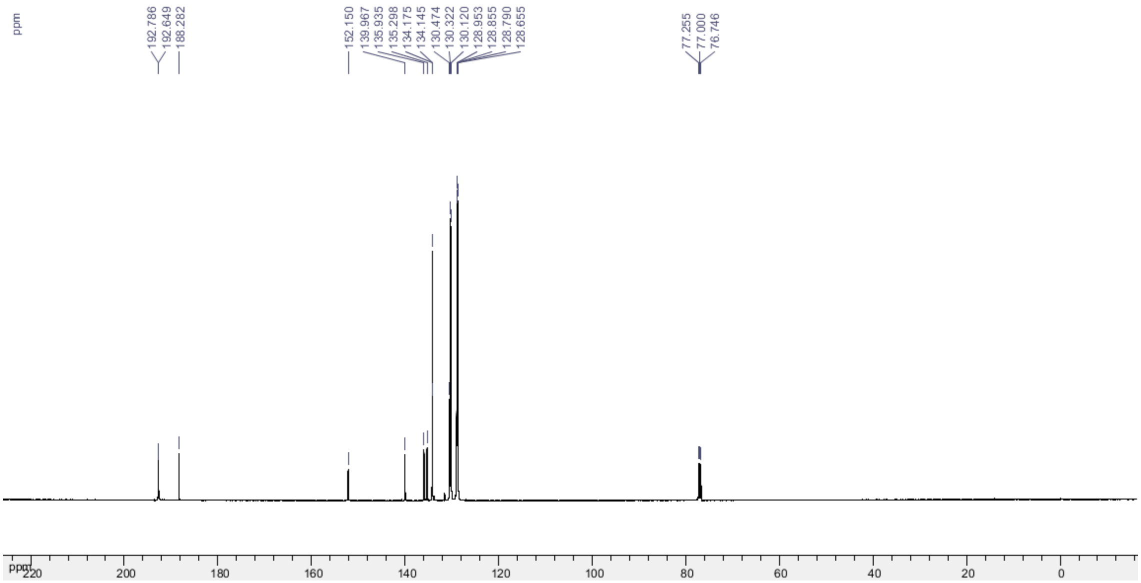

2-Benzoyl-1-(4-chlorophenyl)-4-phenylbut-2-ene-1,4-dione (4ai)

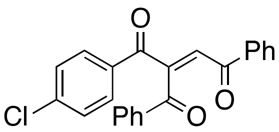

(E)-**4ai**

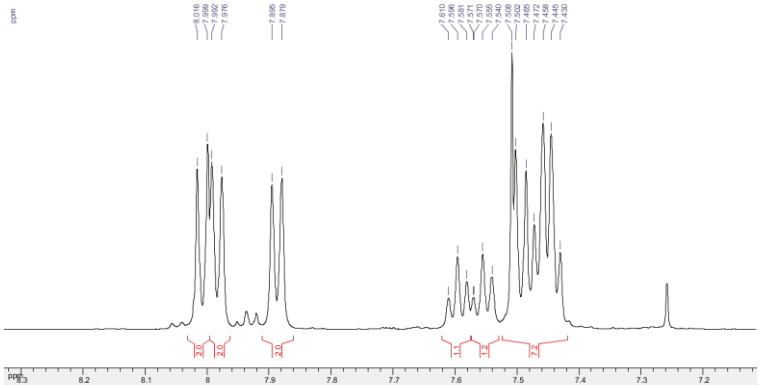

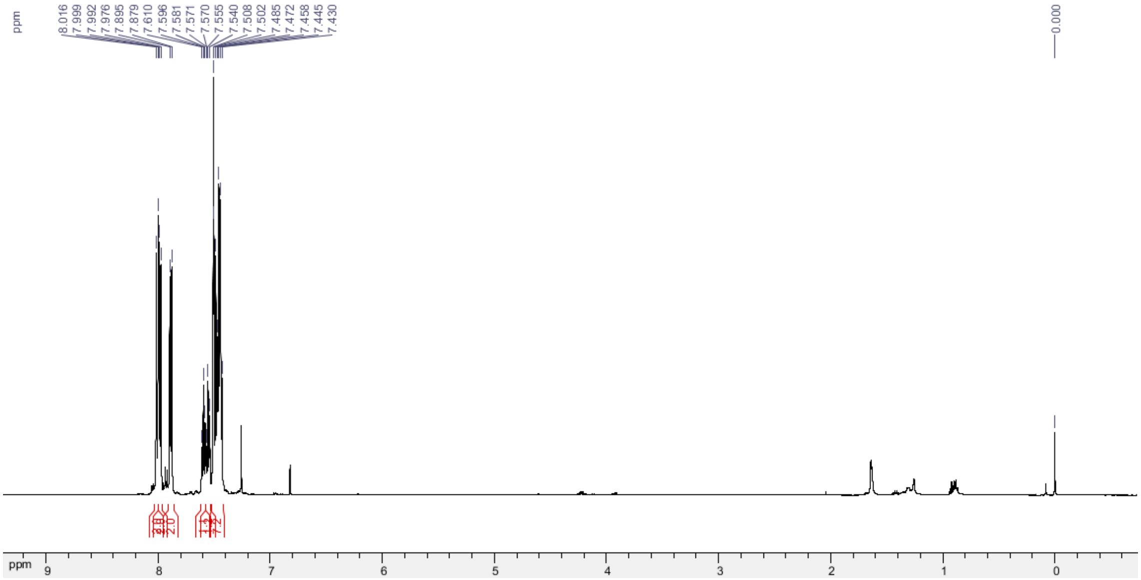

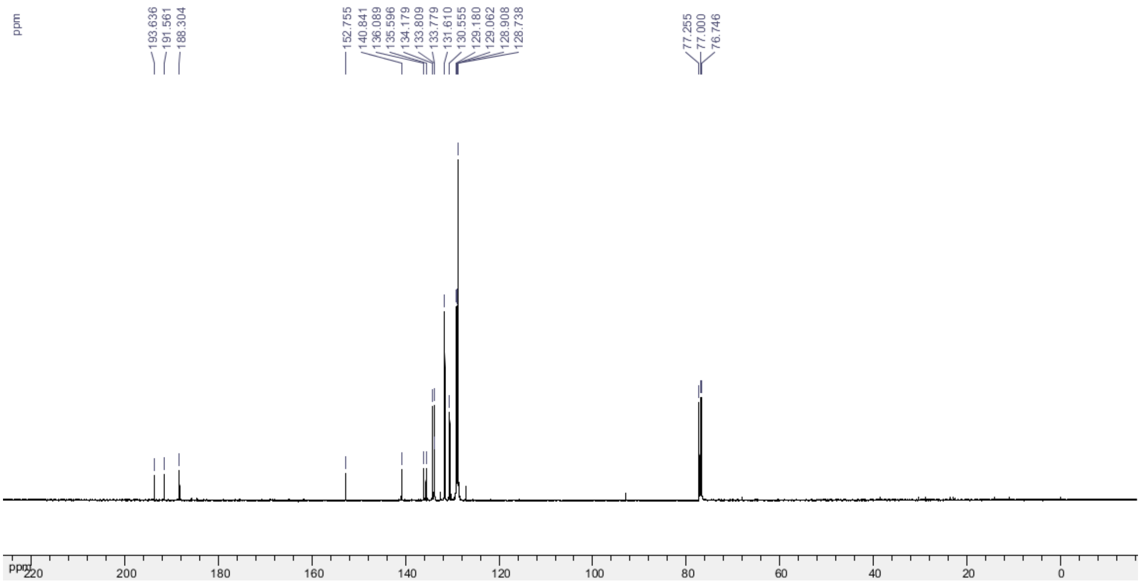

2-Benzoyl-1-(4-bromophenyl)-4-phenylbut-2-ene-1,4-dione (4aj)

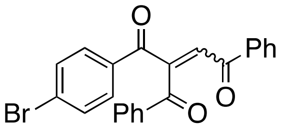

4aj

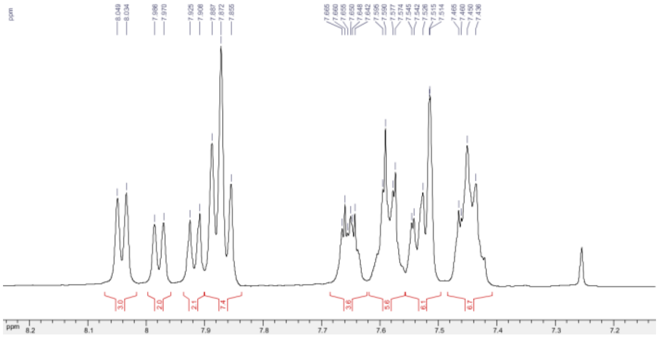

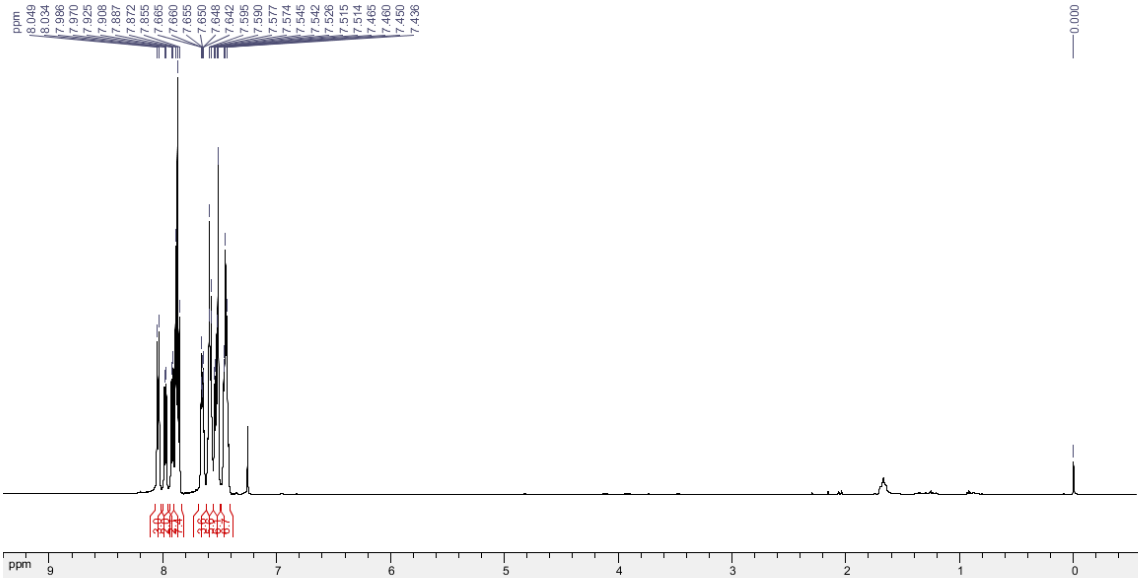

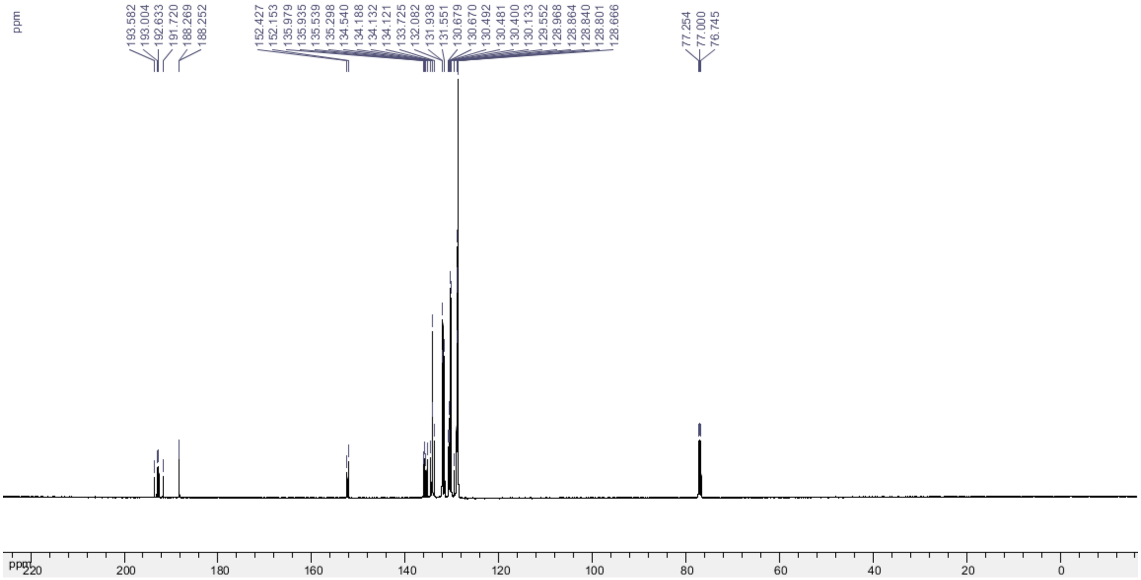

Methyl 4-(2-benzoyl-4-oxo-4-phenylbut-2-enoyl)benzoate (4ak)

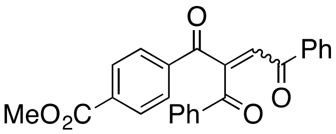

4ak

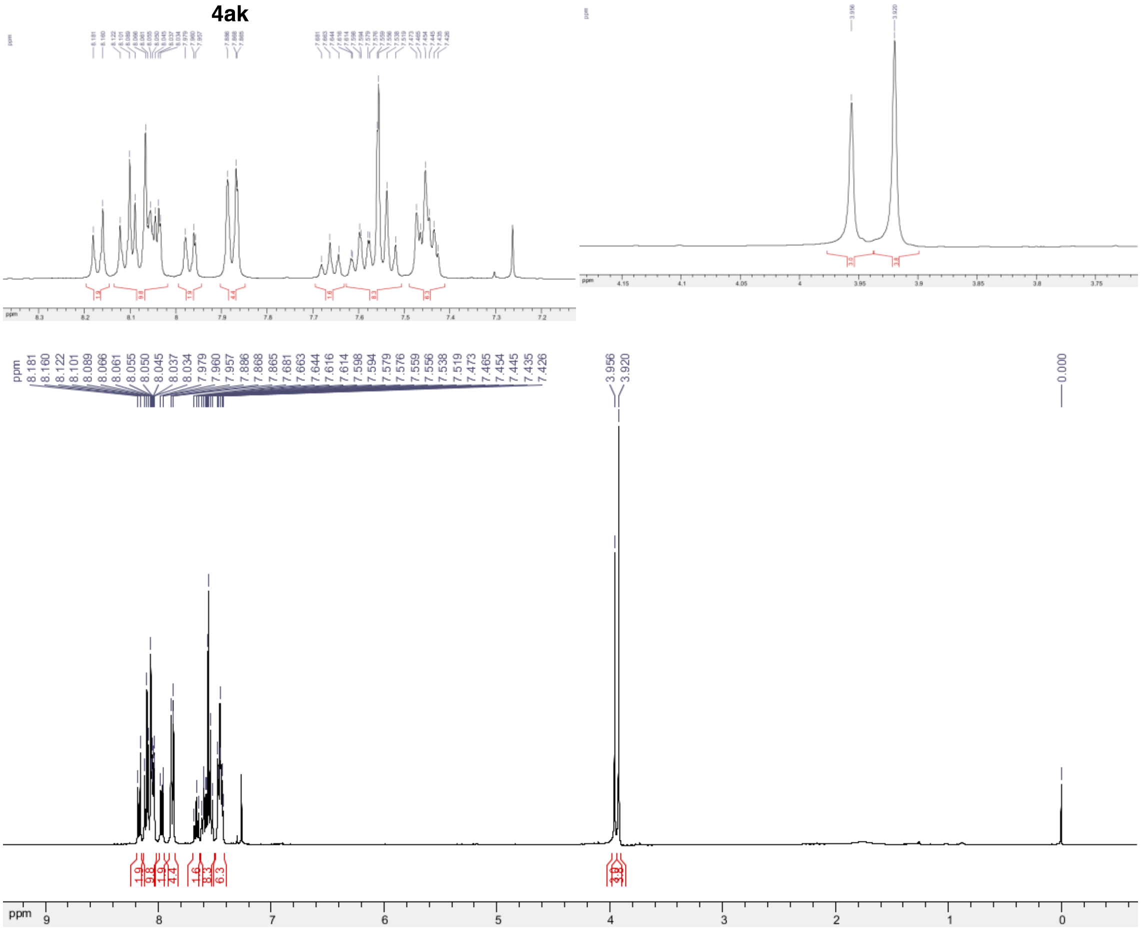

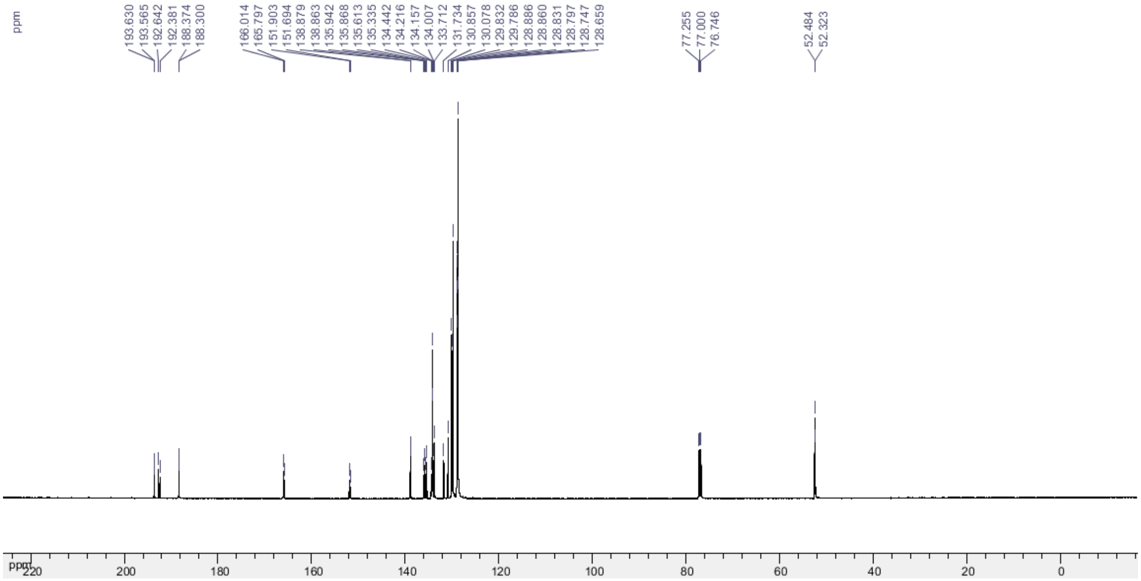

2-Benzoyl-1-(4-cyanophenyl)-4-phenylbut-2-ene-1,4-dione (4al)

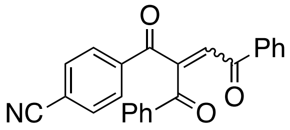

4al

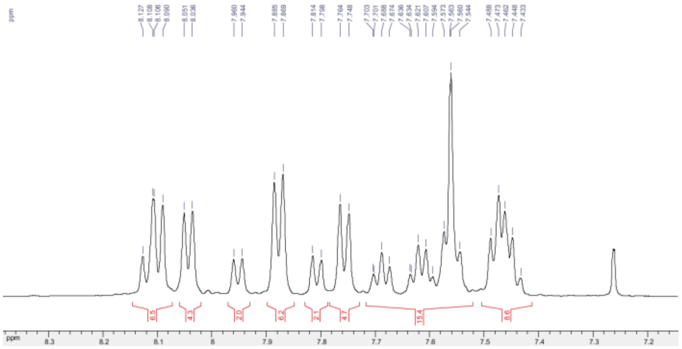

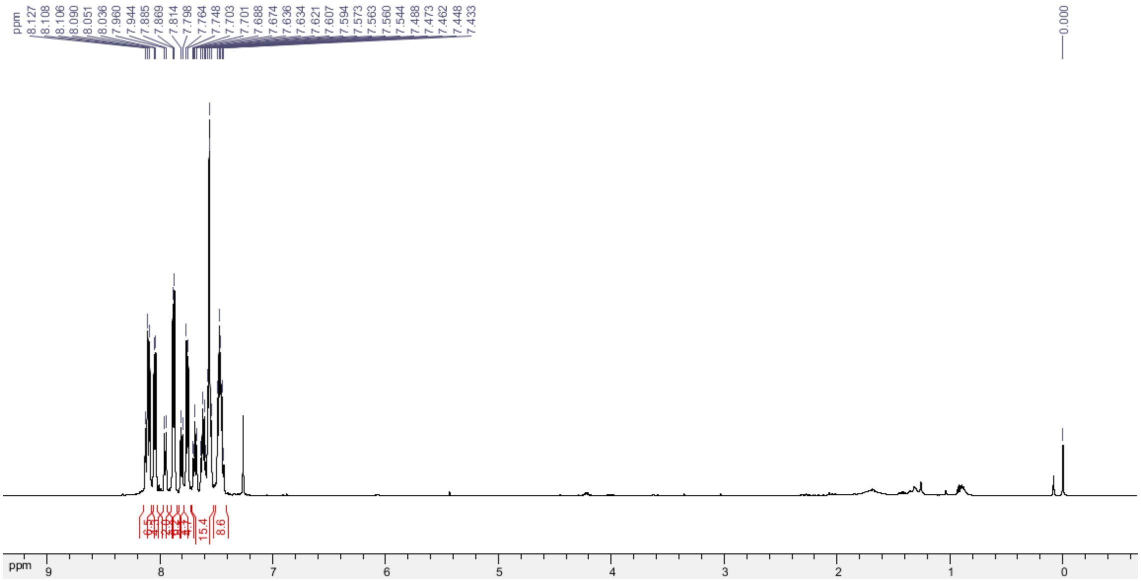

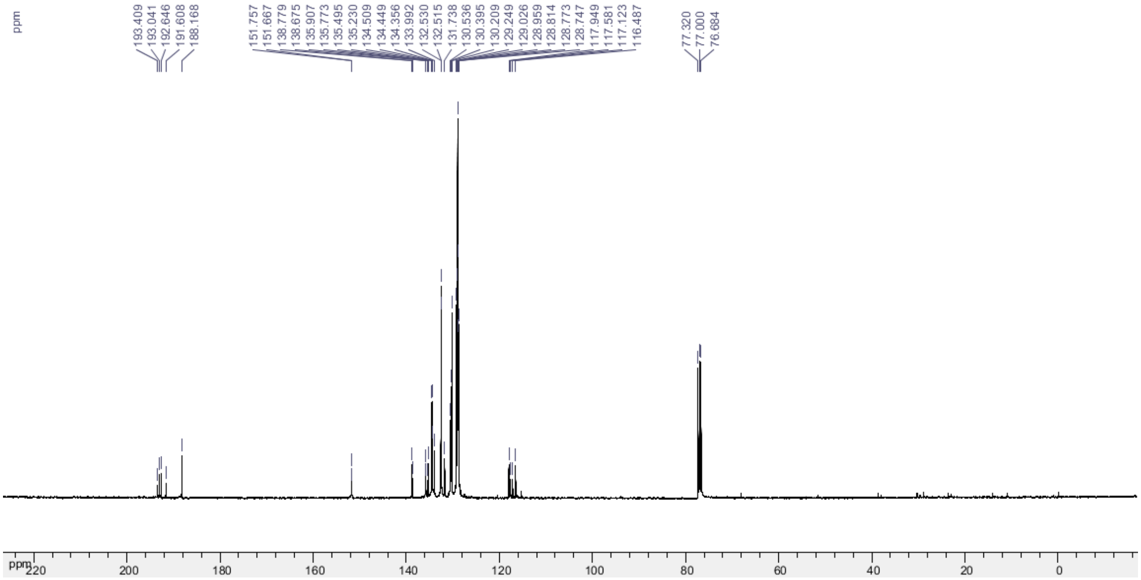

2-Benzoyl-1-(furan-2-yl)-4-phenylbut-2-ene-1,4-dione (4am)

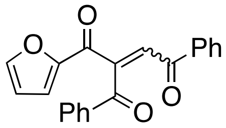

4am

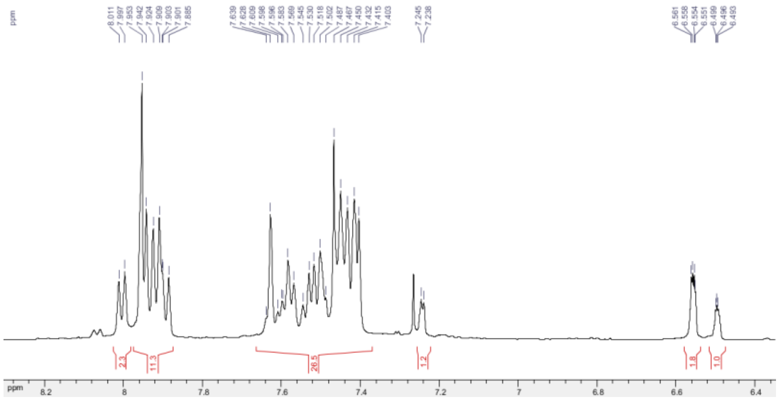

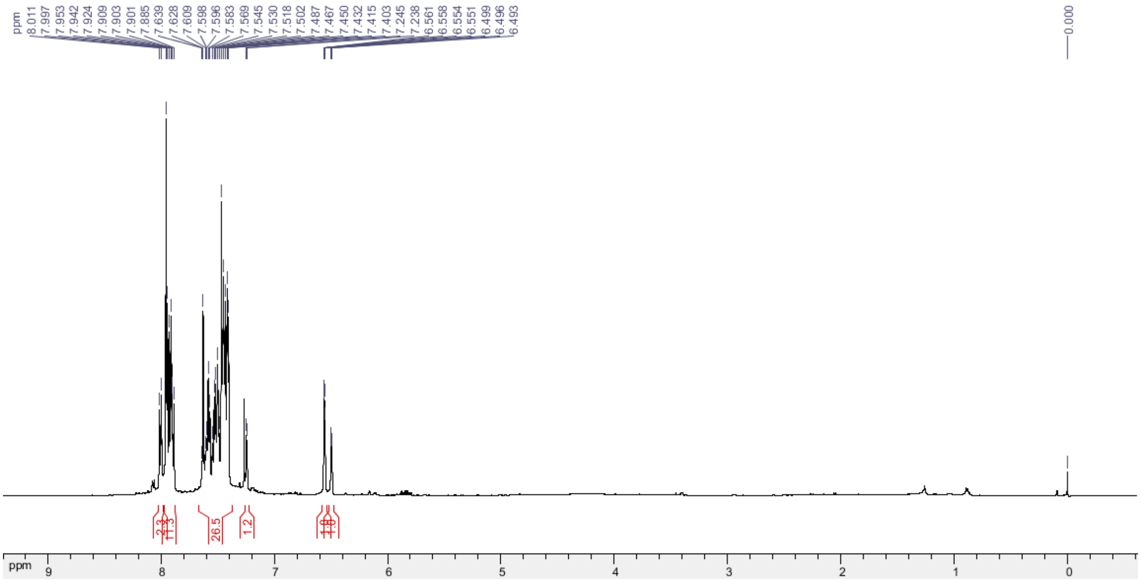

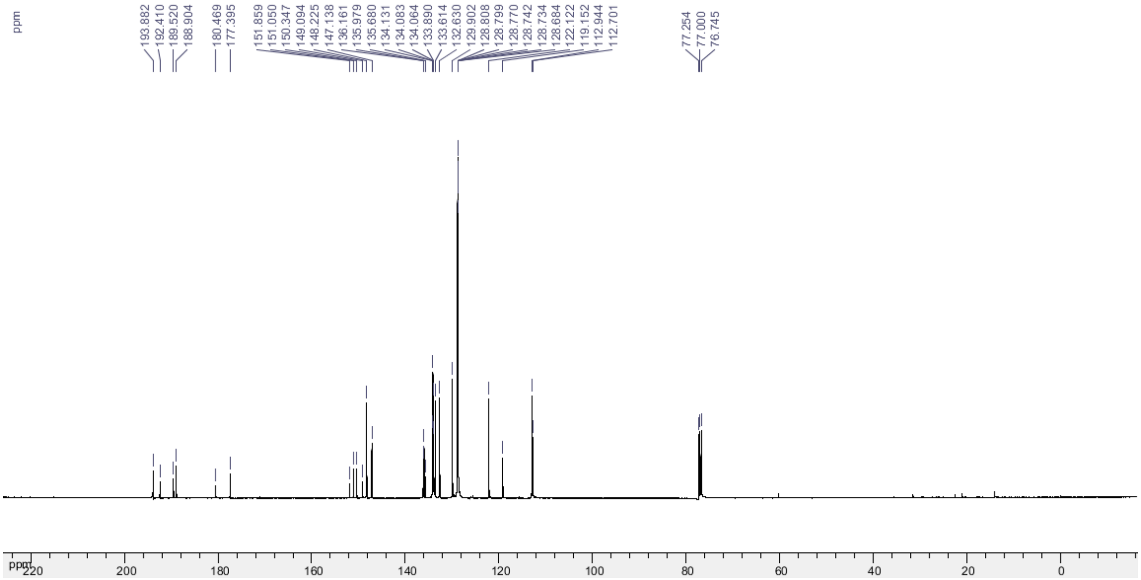

2-Benzoyl-4-phenyl-1-(thiophen-2-yl)but-2-ene-1,4-dione (4an)

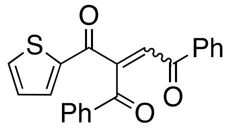

4an

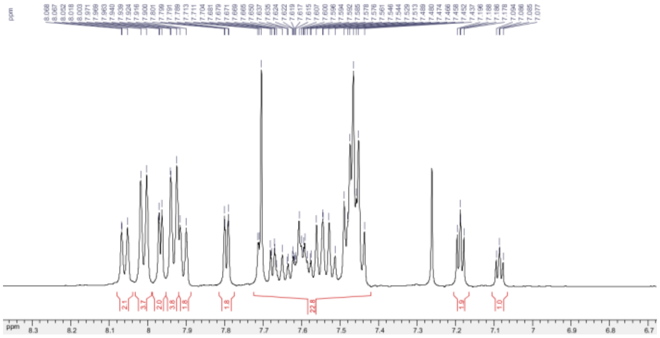

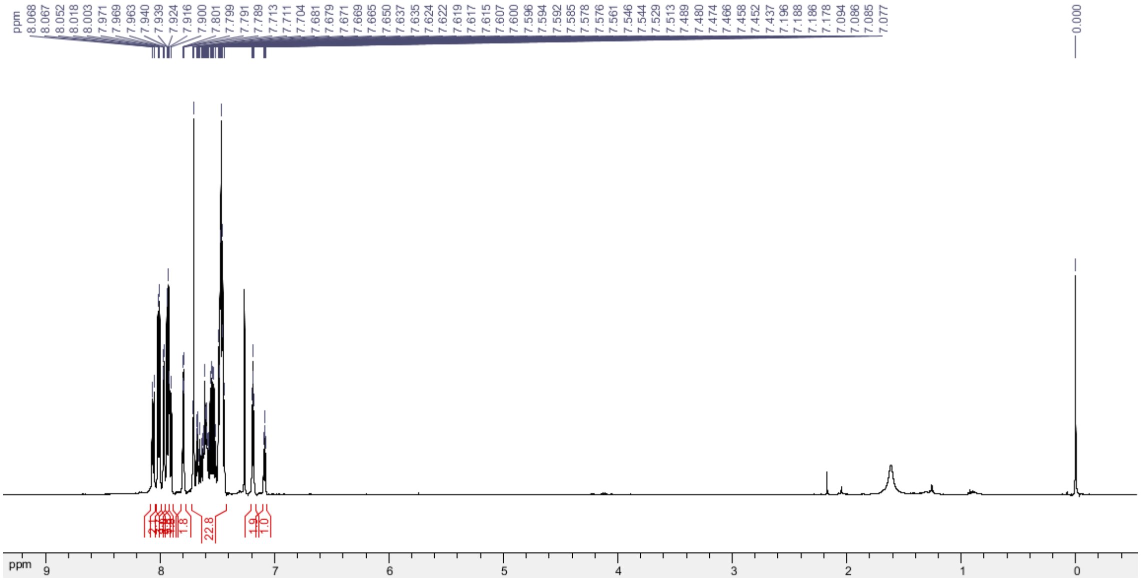

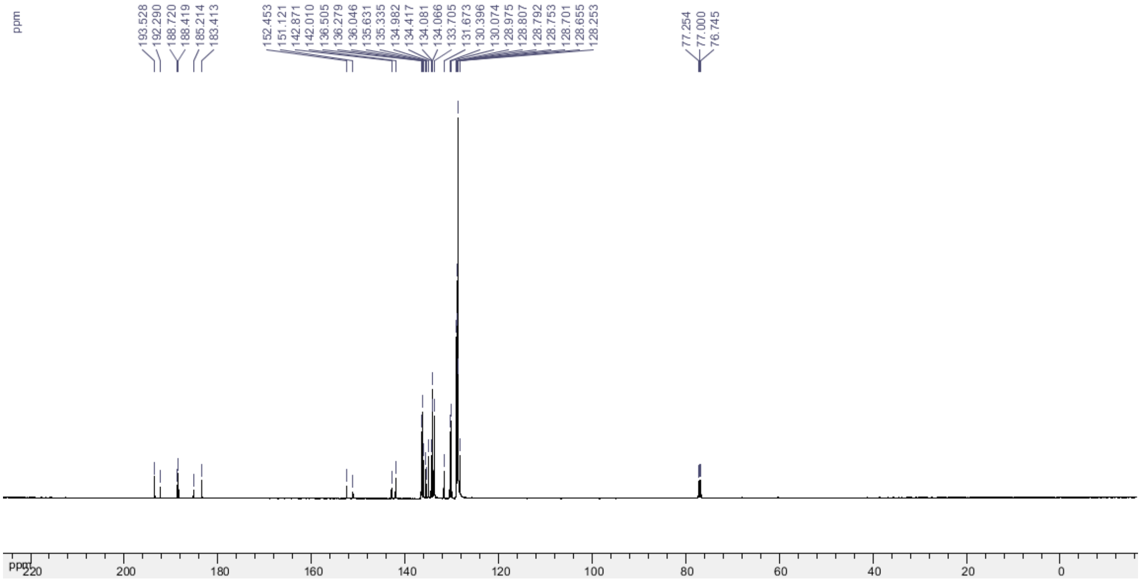

2-Benzoyl-4-phenyl-1-(thiophen-3-yl)but-2-ene-1,4-dione (4ao)

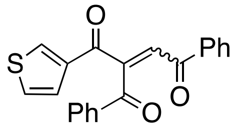

4ao

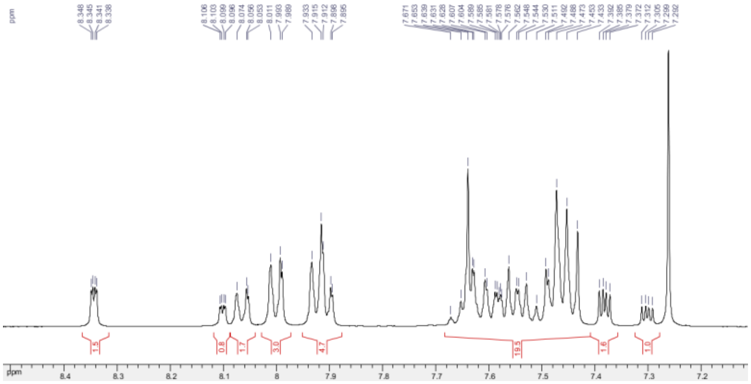

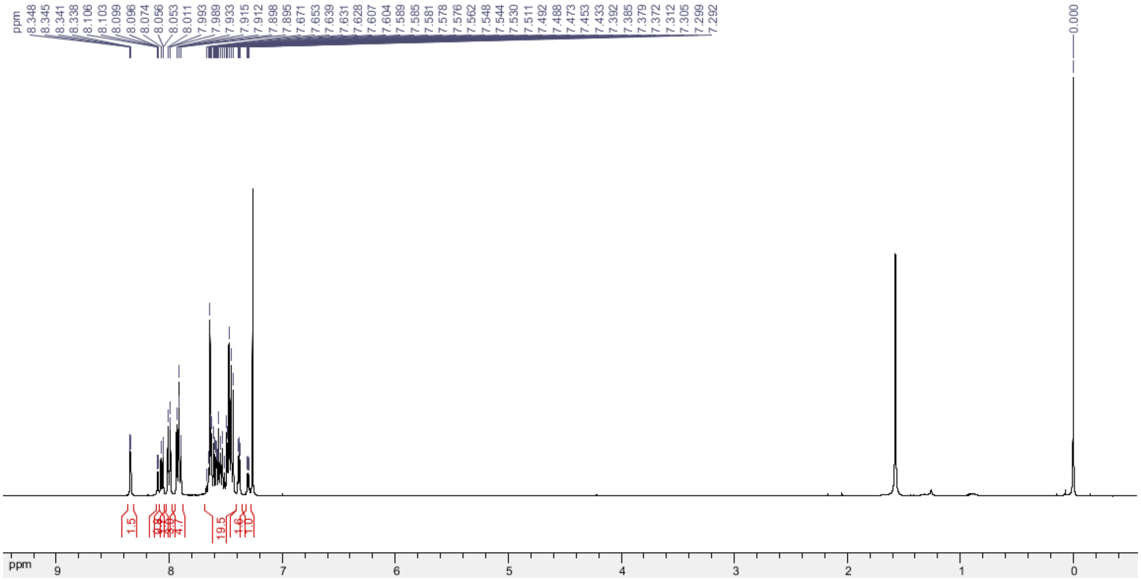

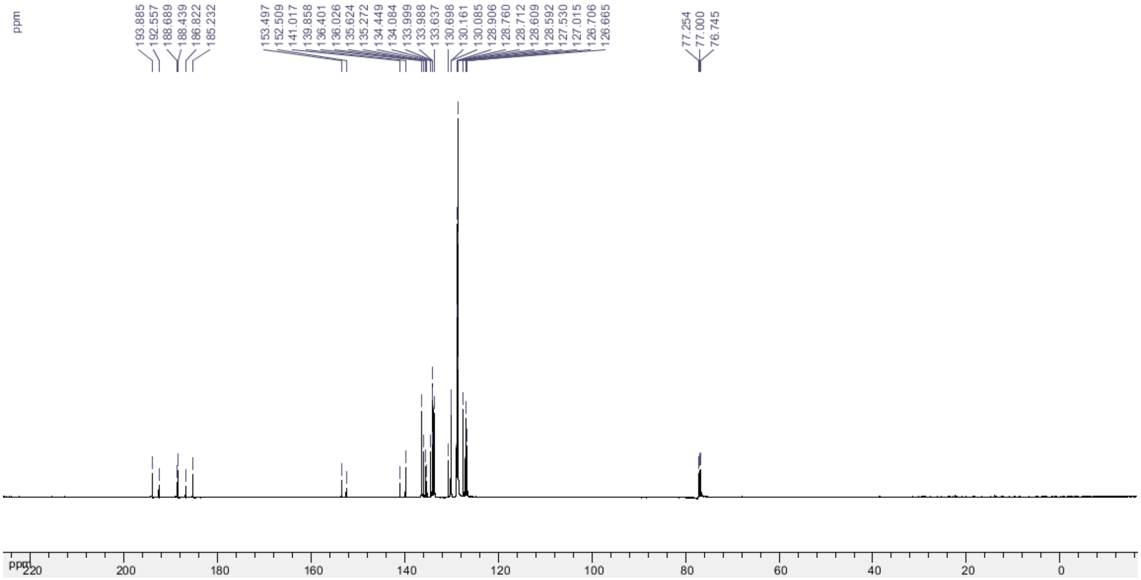

2-Benzoyl-1-(1-naphthyl)-4-phenylbut-2-ene-1,4-dione (4ap)

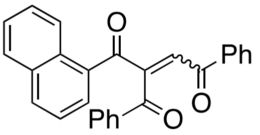

4ap

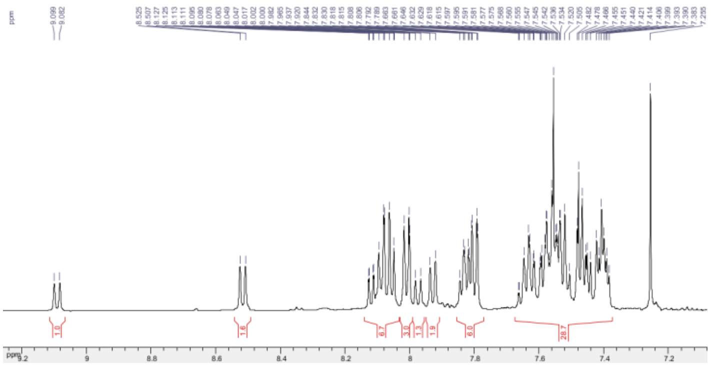

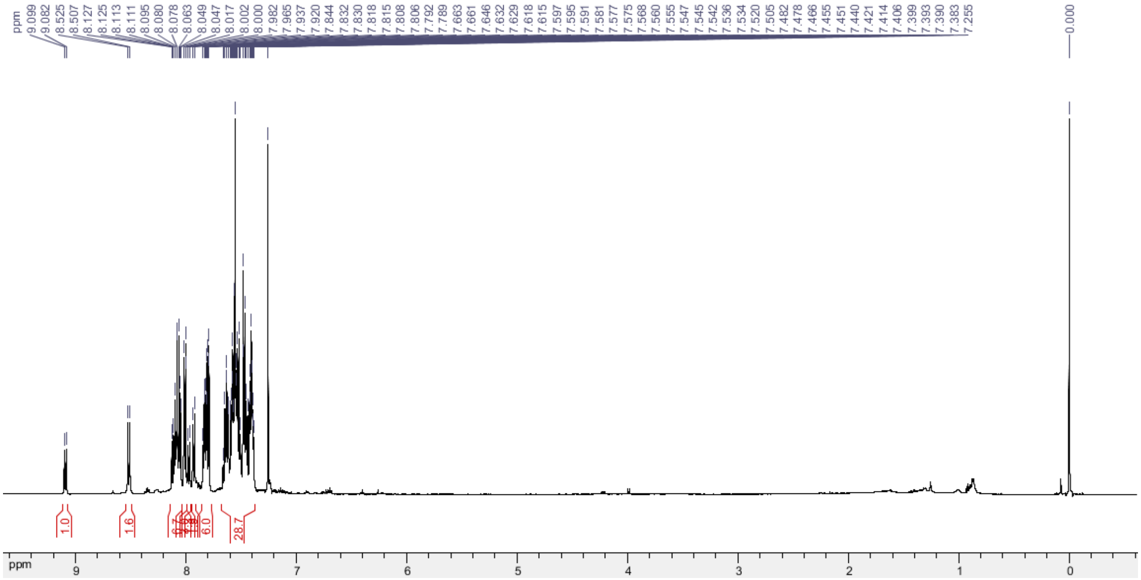

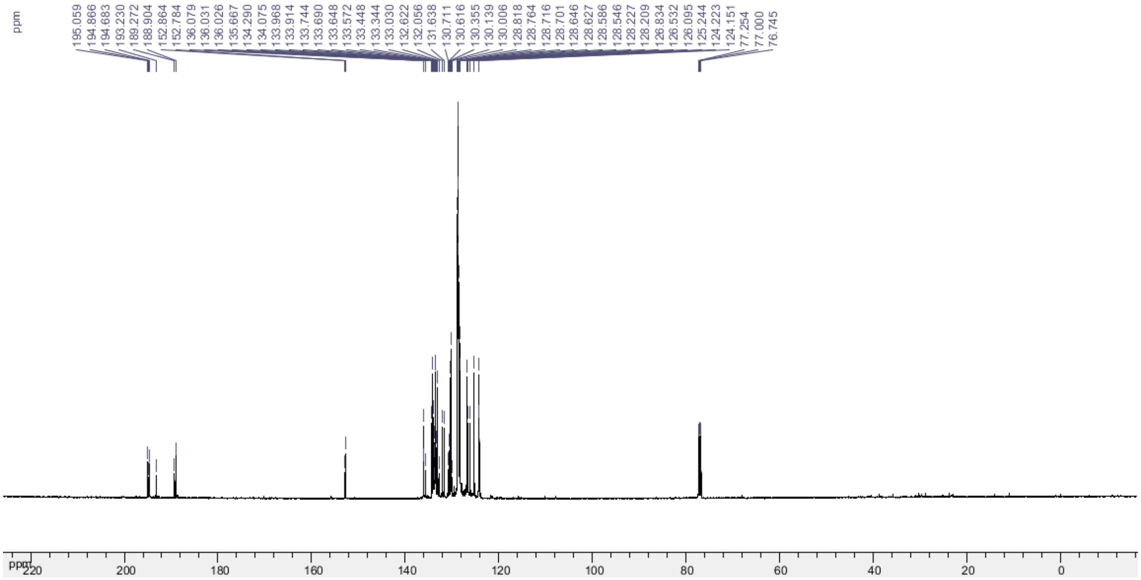

2-Benzoyl-1-(2-naphthyl)-4-phenylbut-2-ene-1,4-dione (4aq)

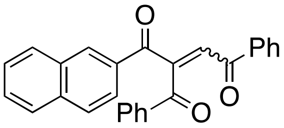

4aq

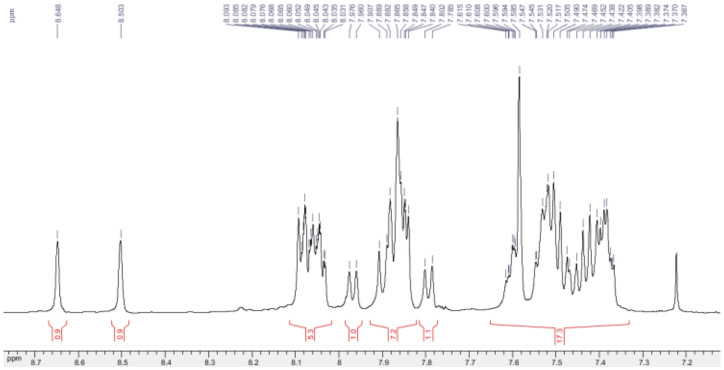

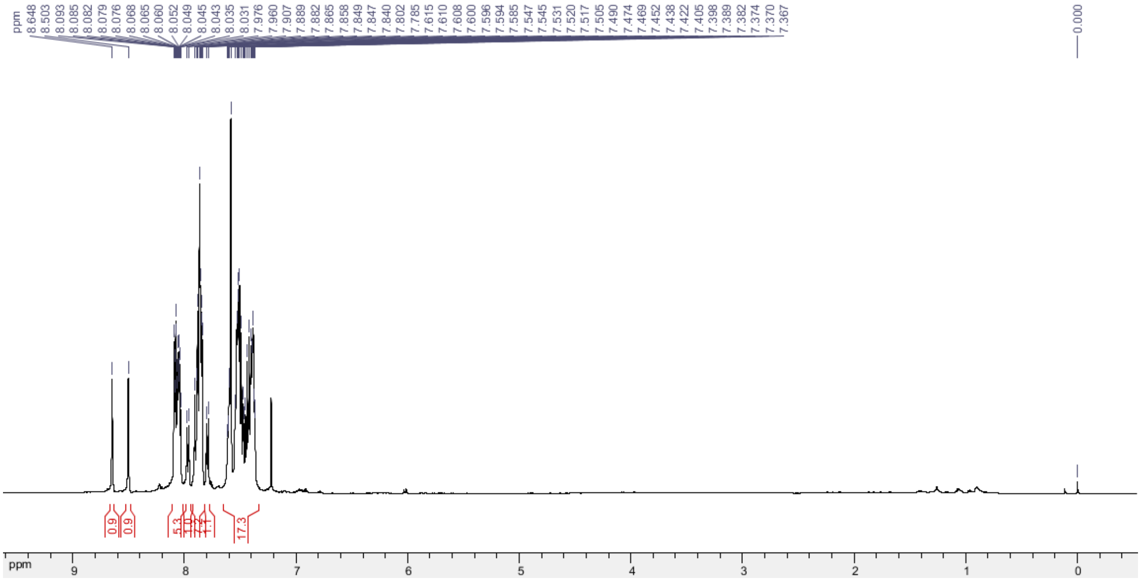

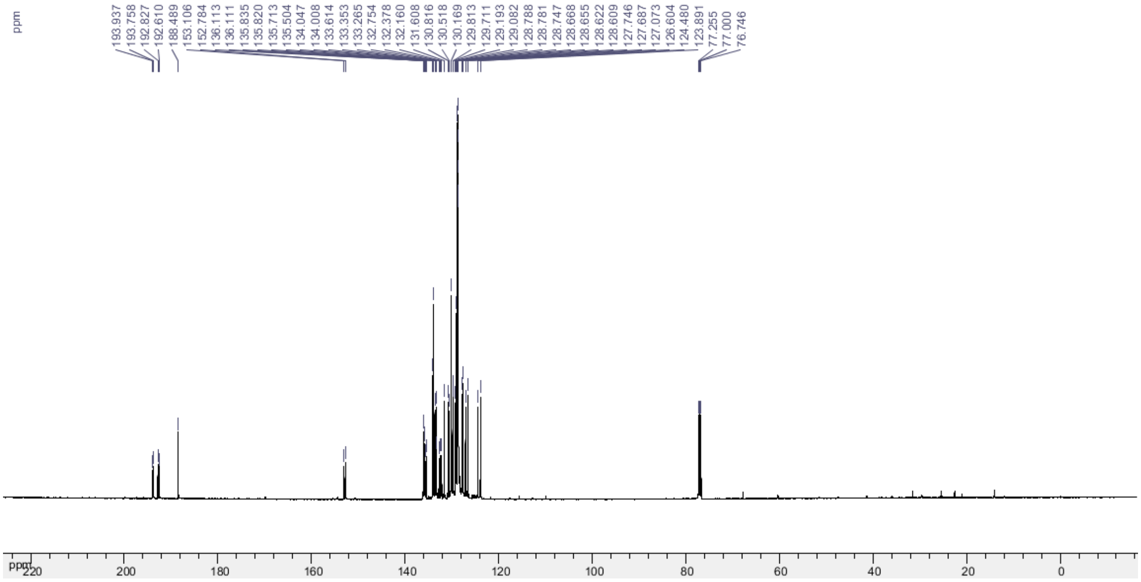

3-Benzoyl-1,6-diphenylhex-2-ene-1,4-dione (4ar)

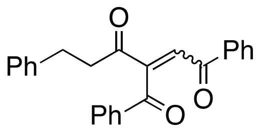

4ar

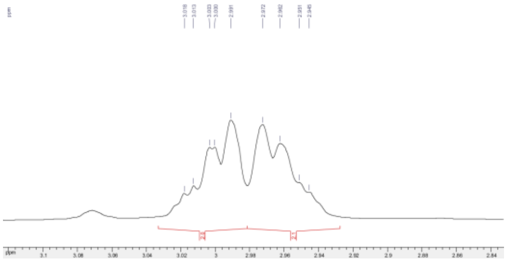

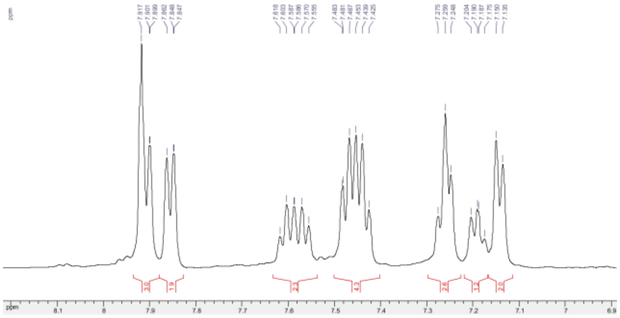

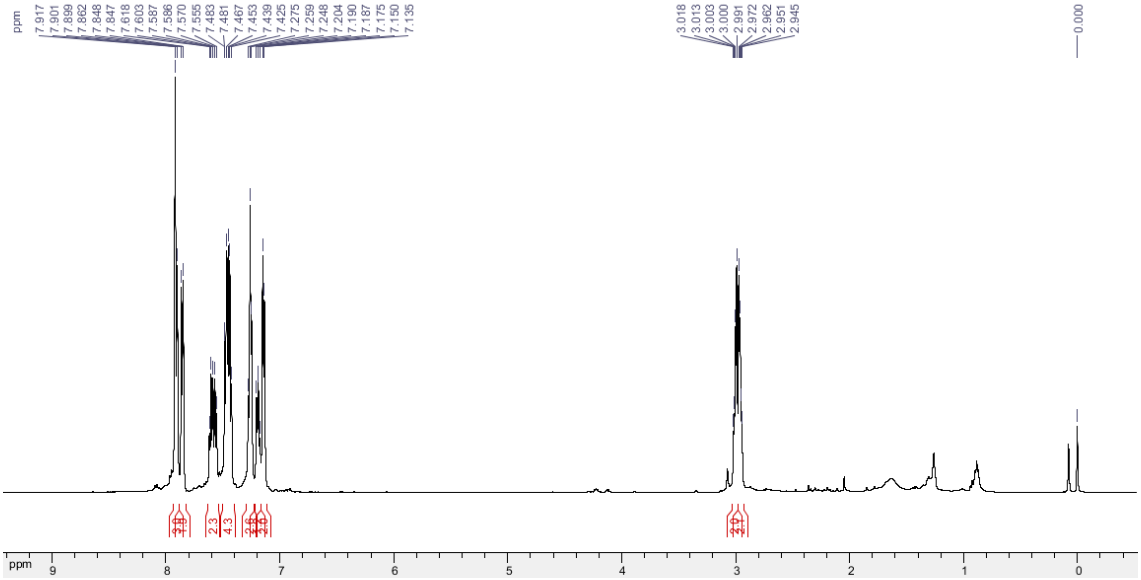

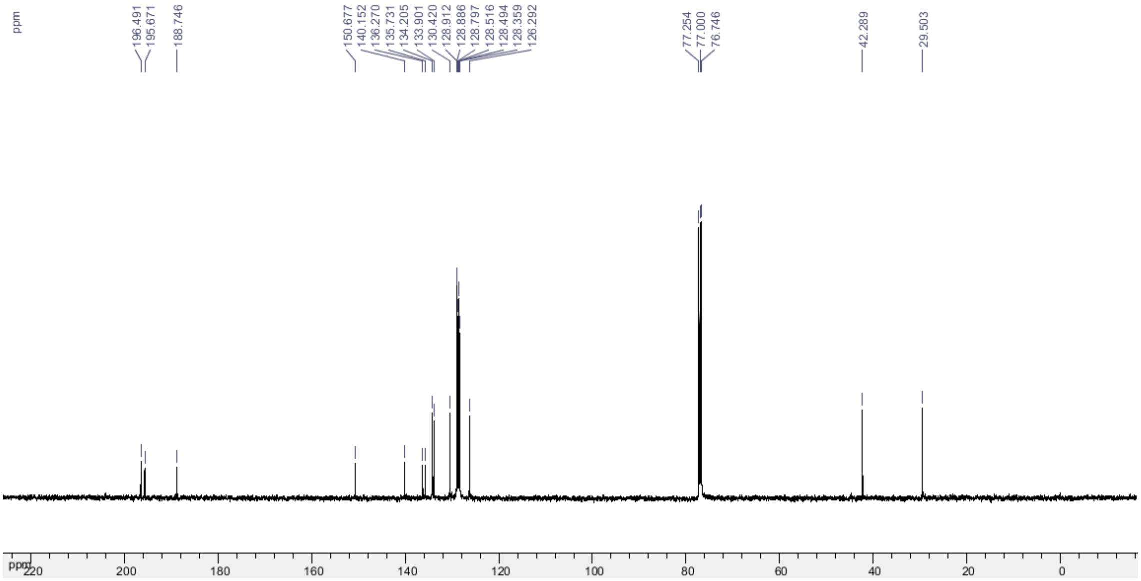

2-Benzoyl-1,4-di-*p*-tolylbut-2-ene-1,4-dione (4ba)

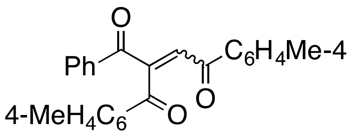

4ba

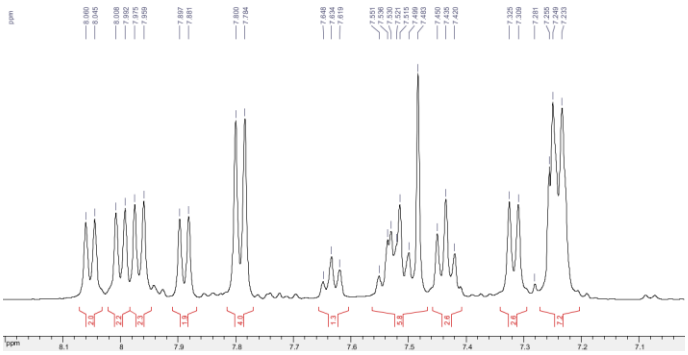

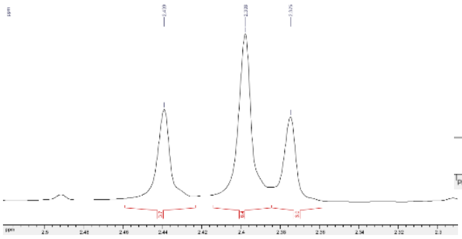

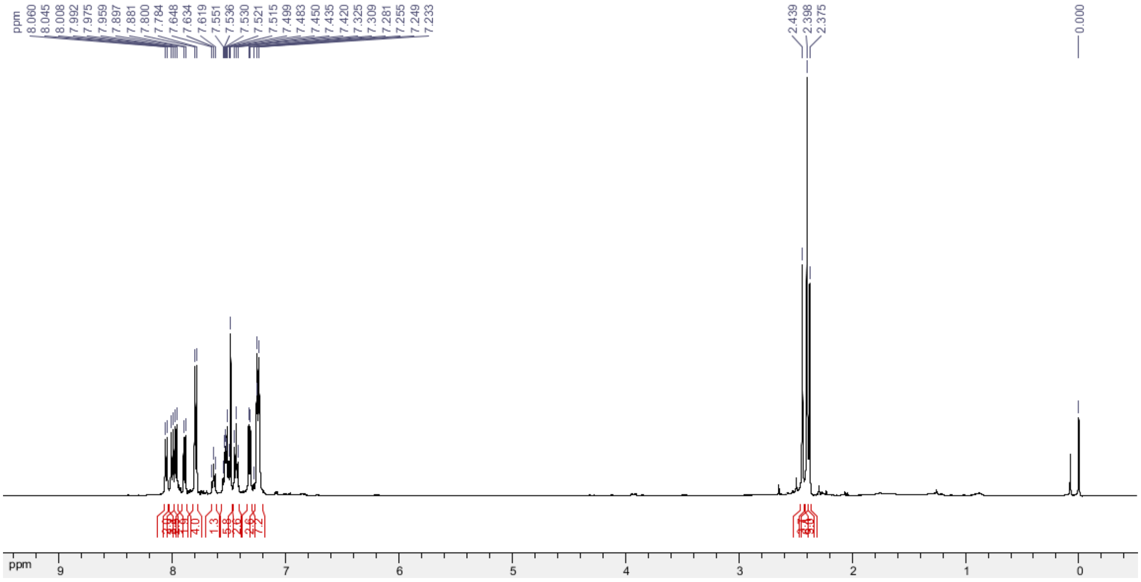

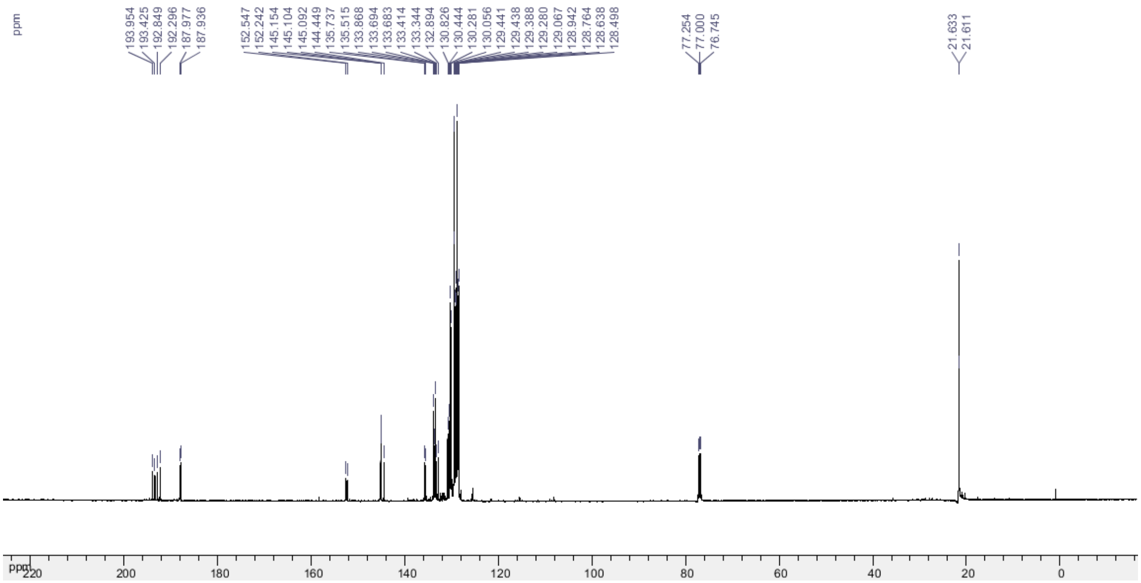

2-Benzoyl-1,4-bis(4-methoxyphenyl)but-2-ene-1,4-dione (4ca)

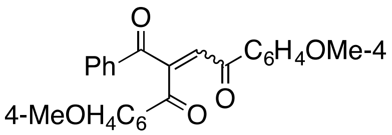

4ca

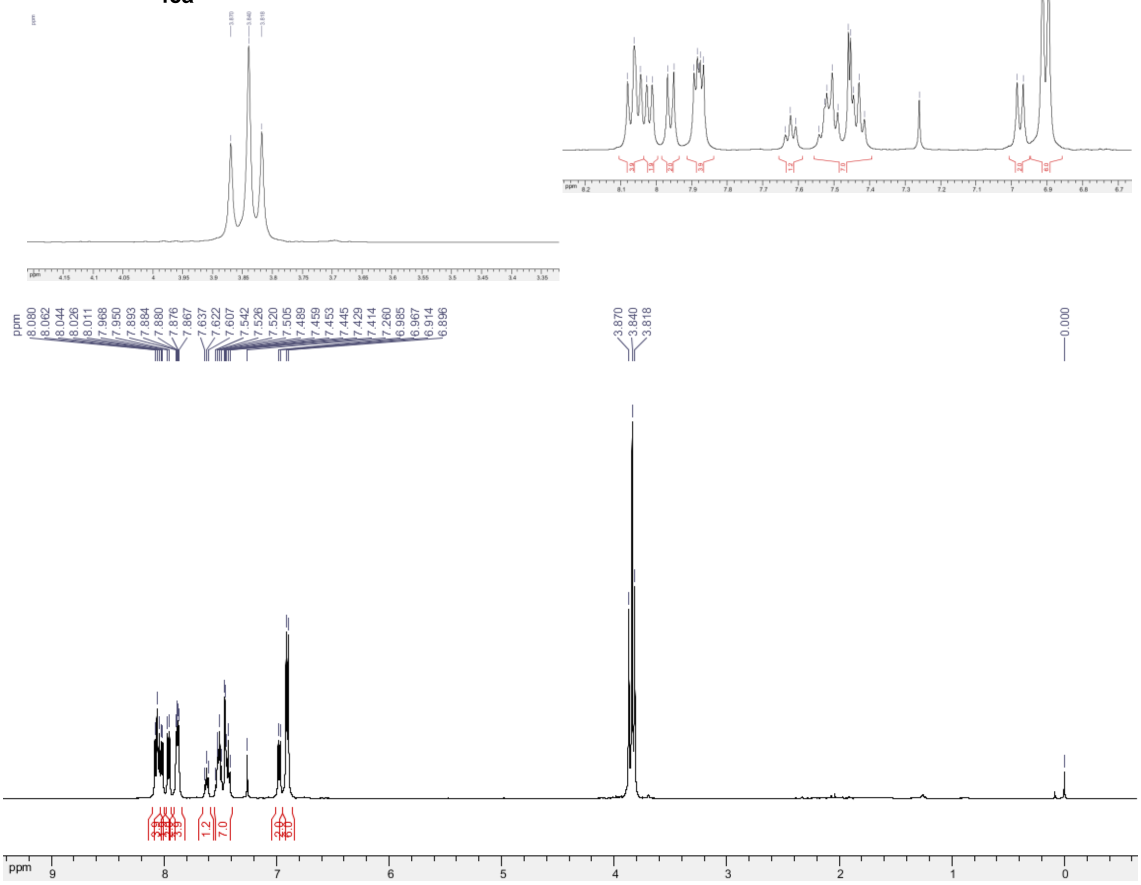

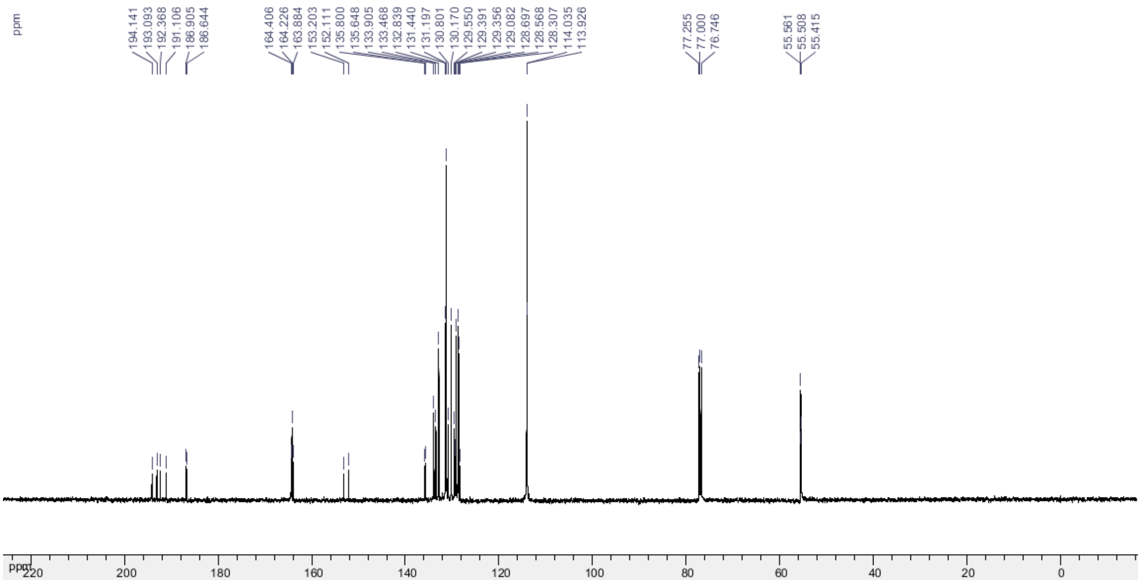

2-Benzoyl-1,4-bis(4-chlorophenyl)but-2-ene-1,4-dione (4da)

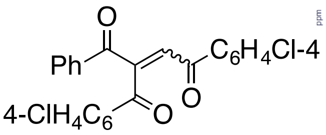

4da

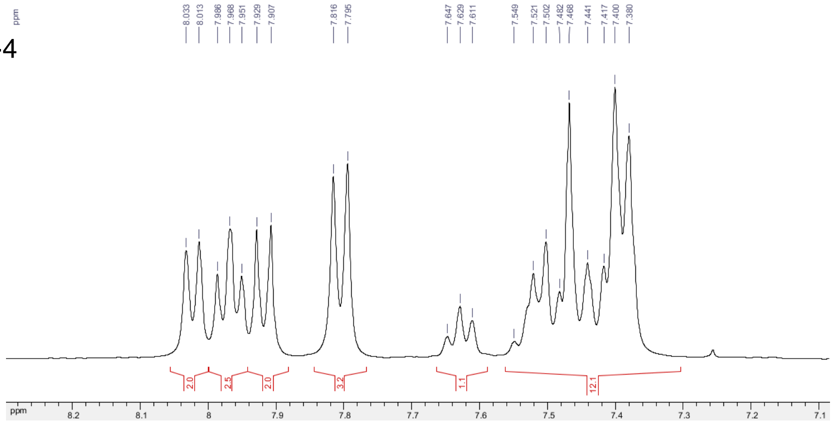

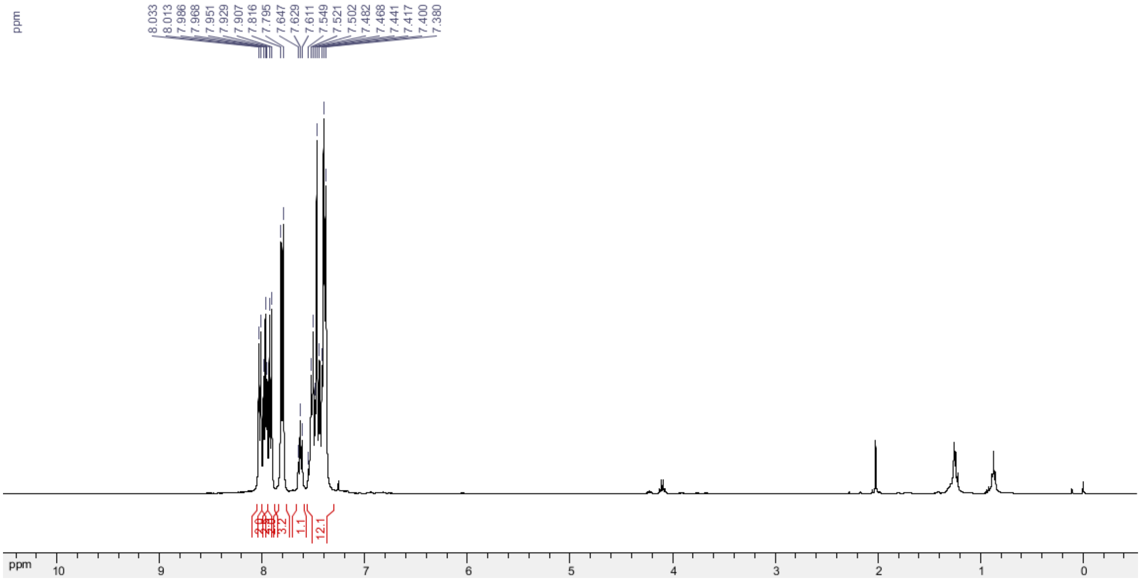

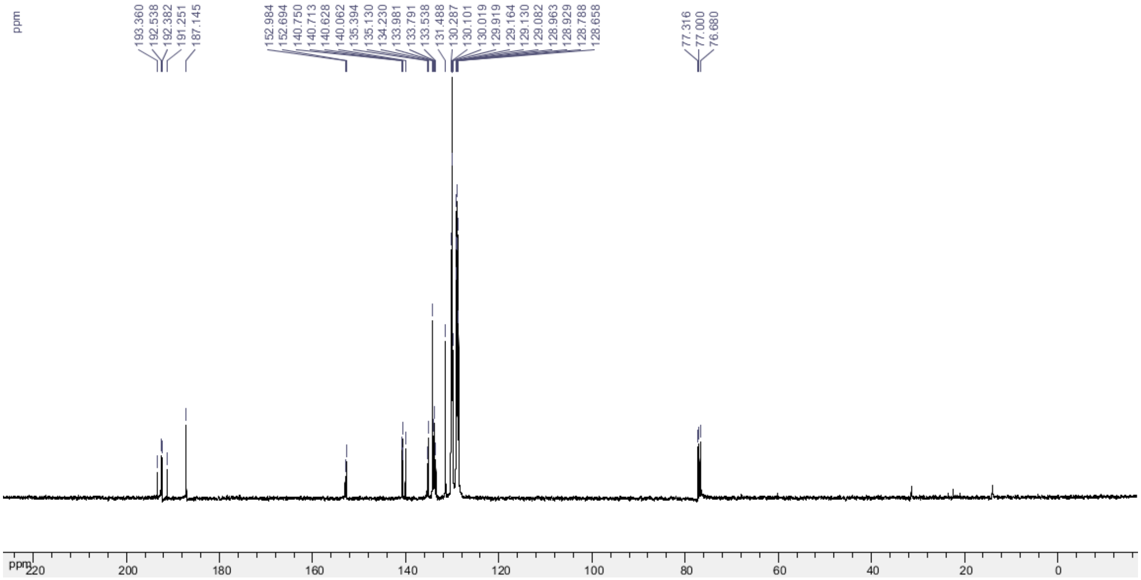

2-Benzoyl-1,4-bis(4-bromophenyl)but-2-ene-1,4-dione (4ea)

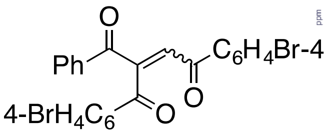

4ea

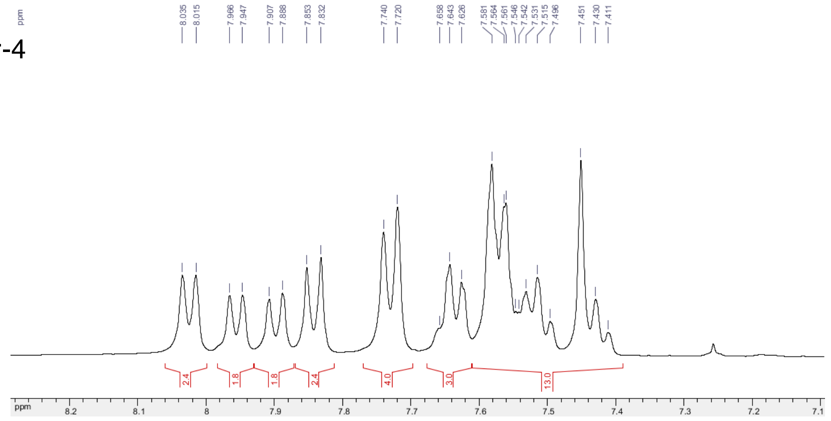

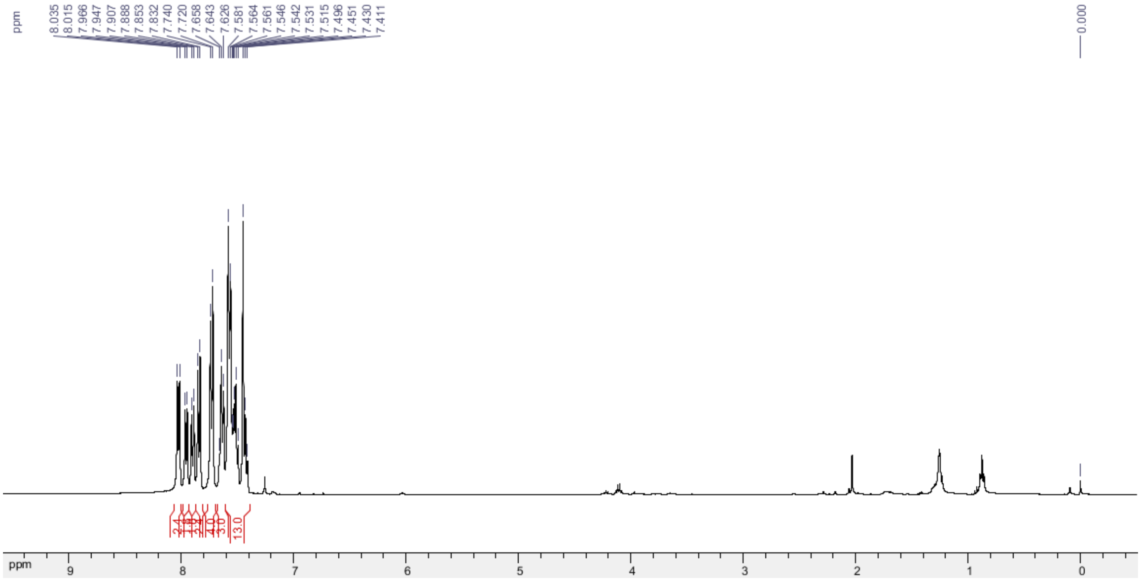

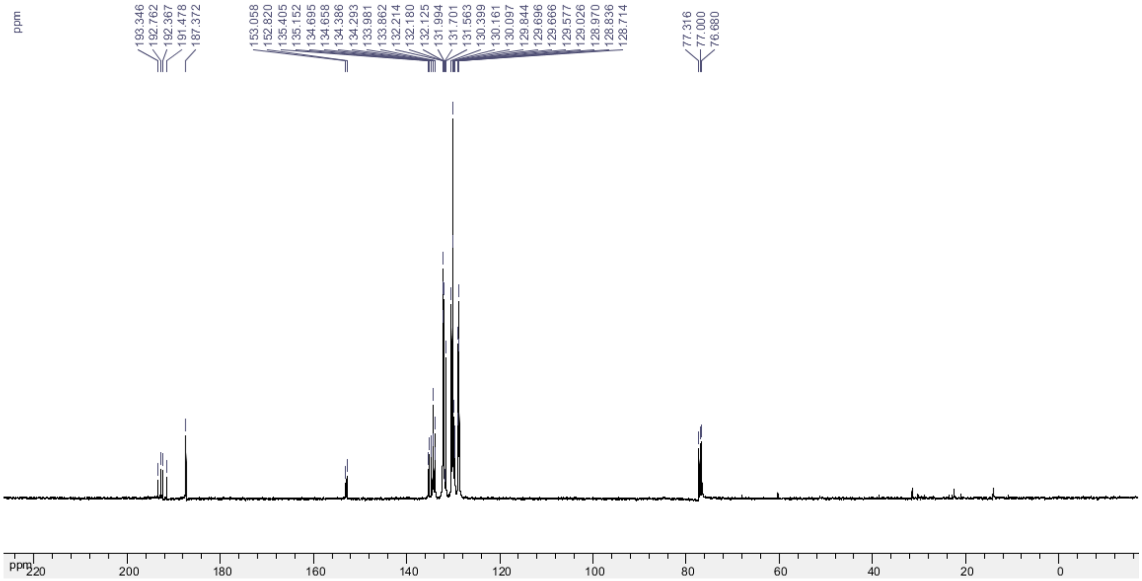

2-Benzoylcyclooctane-1,4-dione (7a)

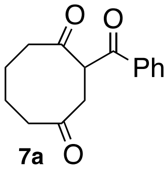

7a

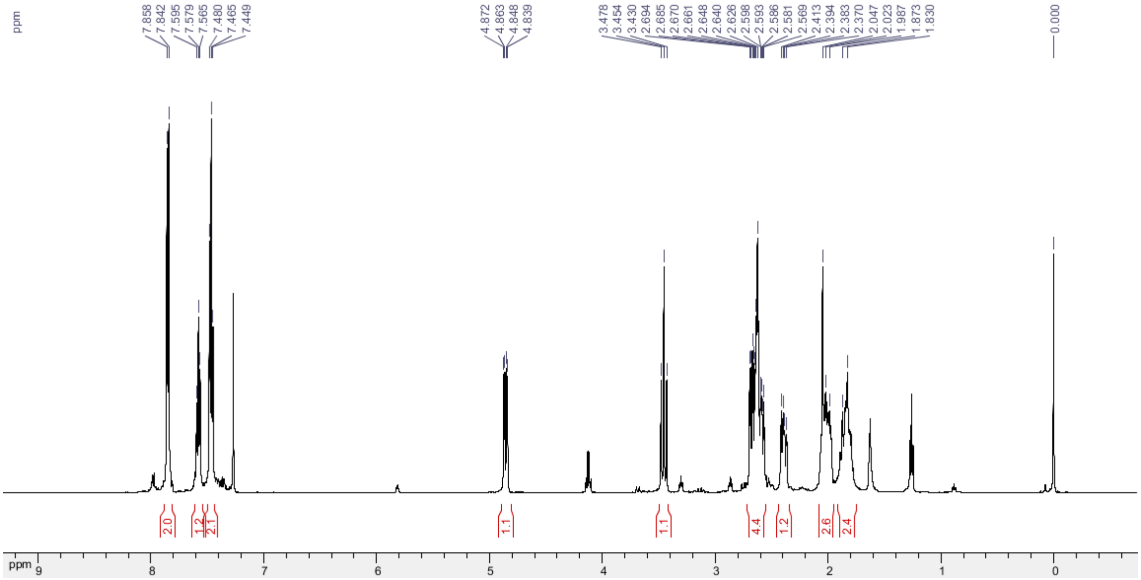

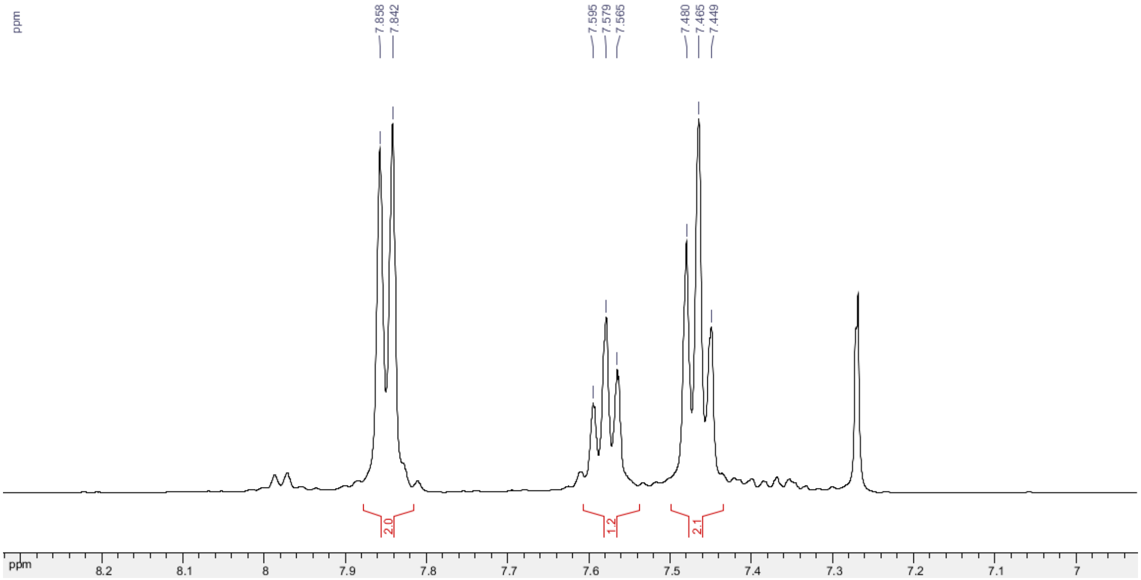

2-Benzoylcyclooctane-1,4-dione (7a)

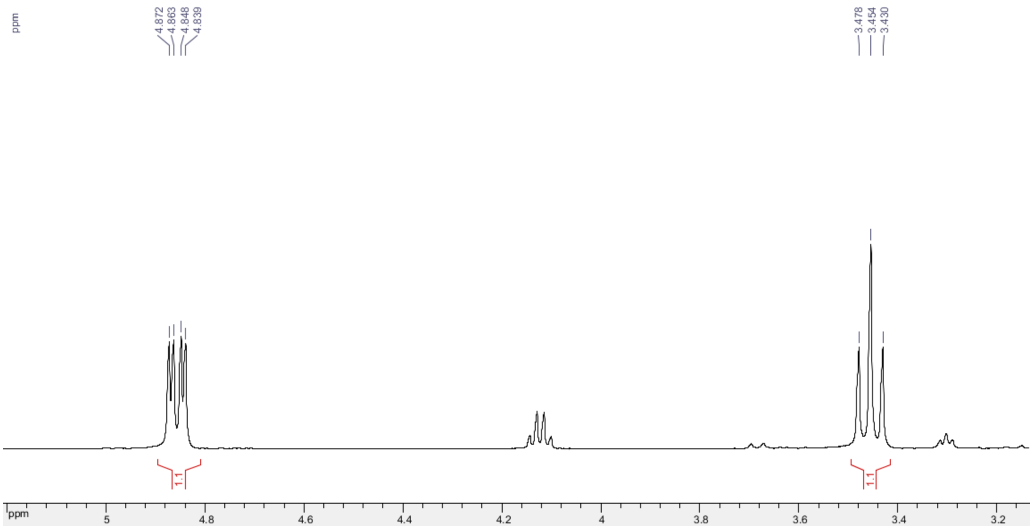

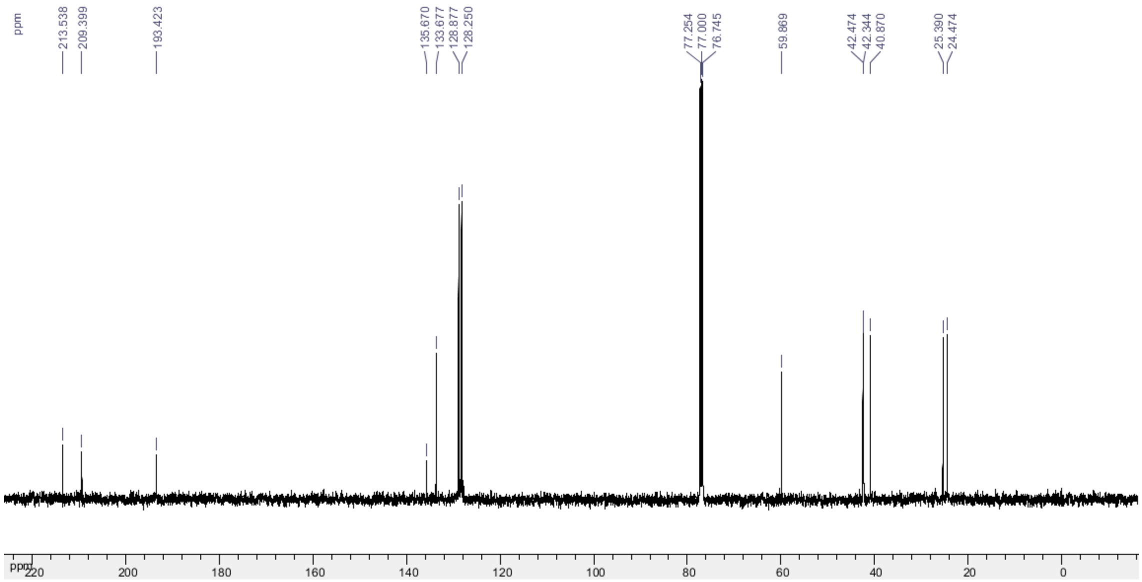

2-Benzoylcyclononane-1,4-dione (7b)

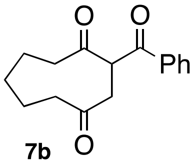

7b

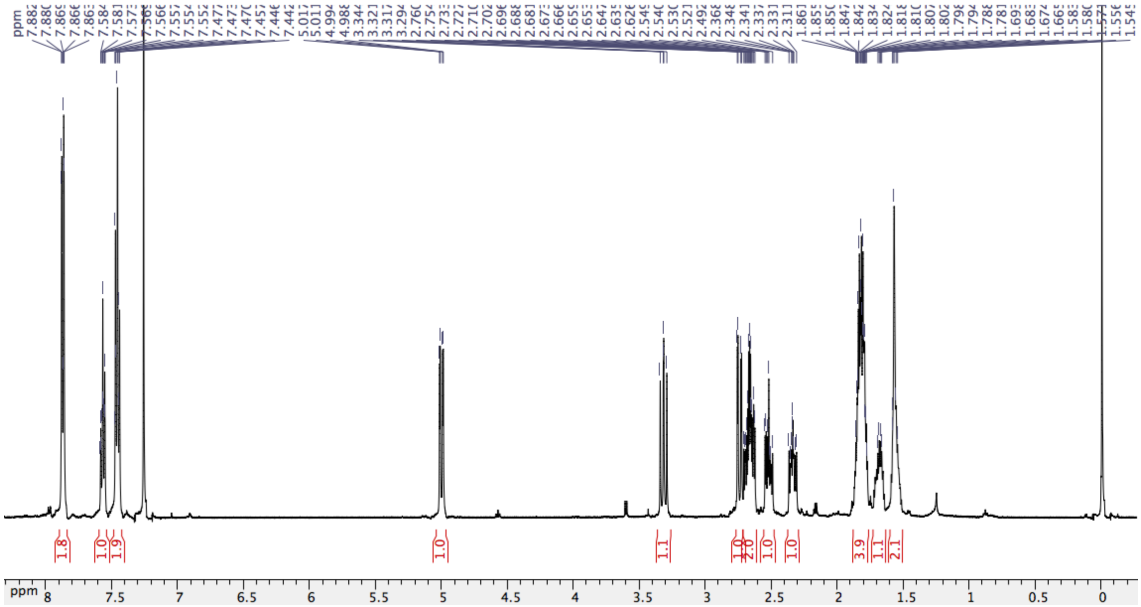

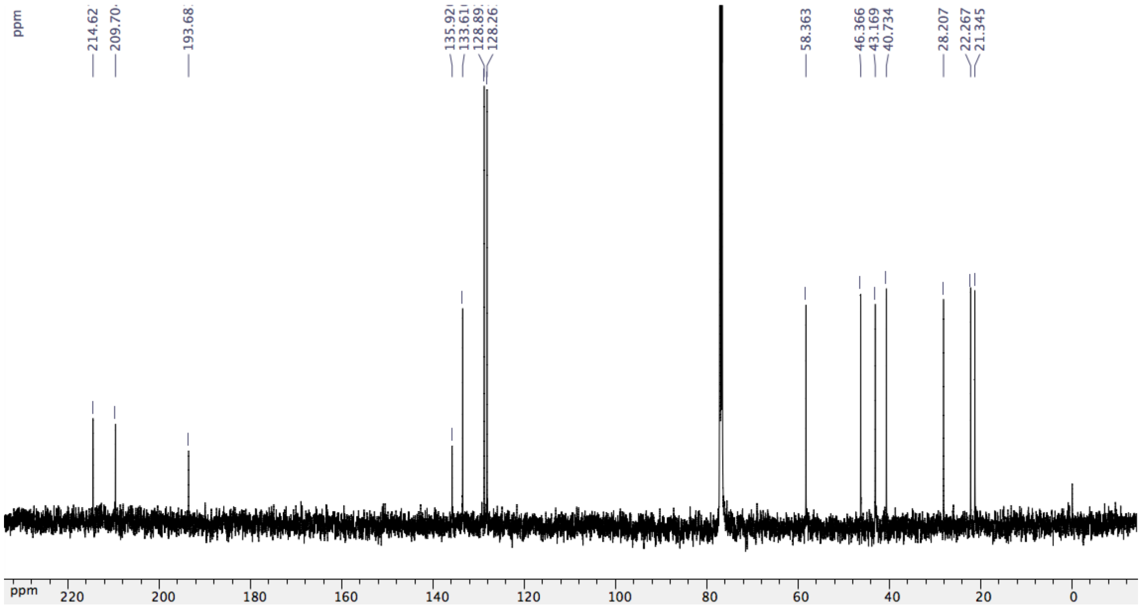

2-Benzoylcyclotetradecane-1,4-dione (7c)

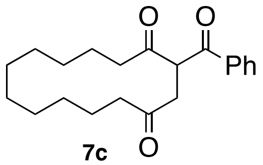

7c

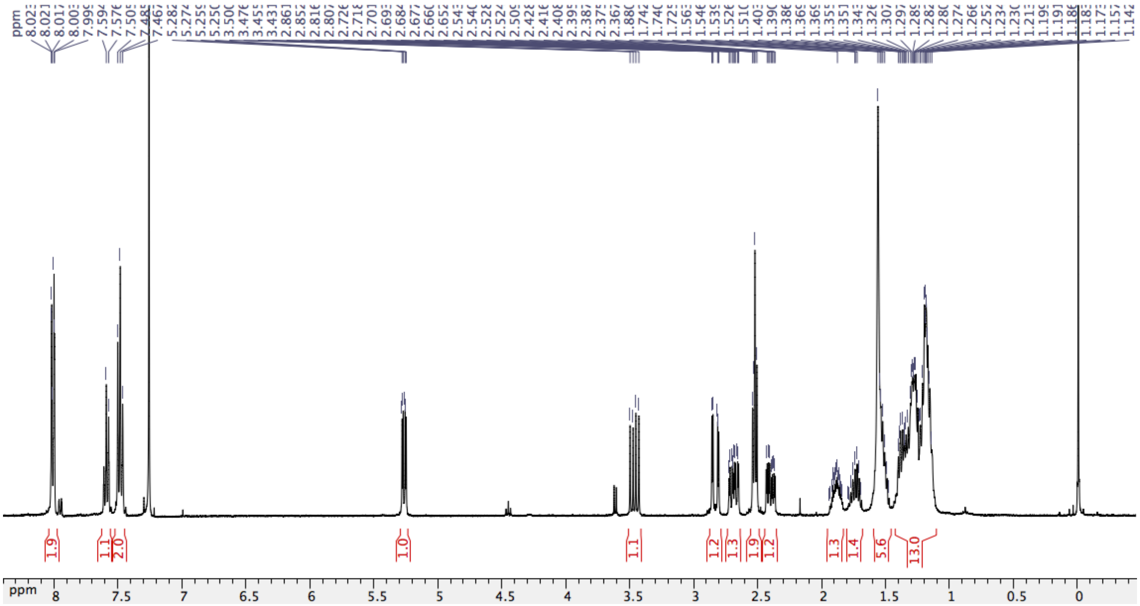

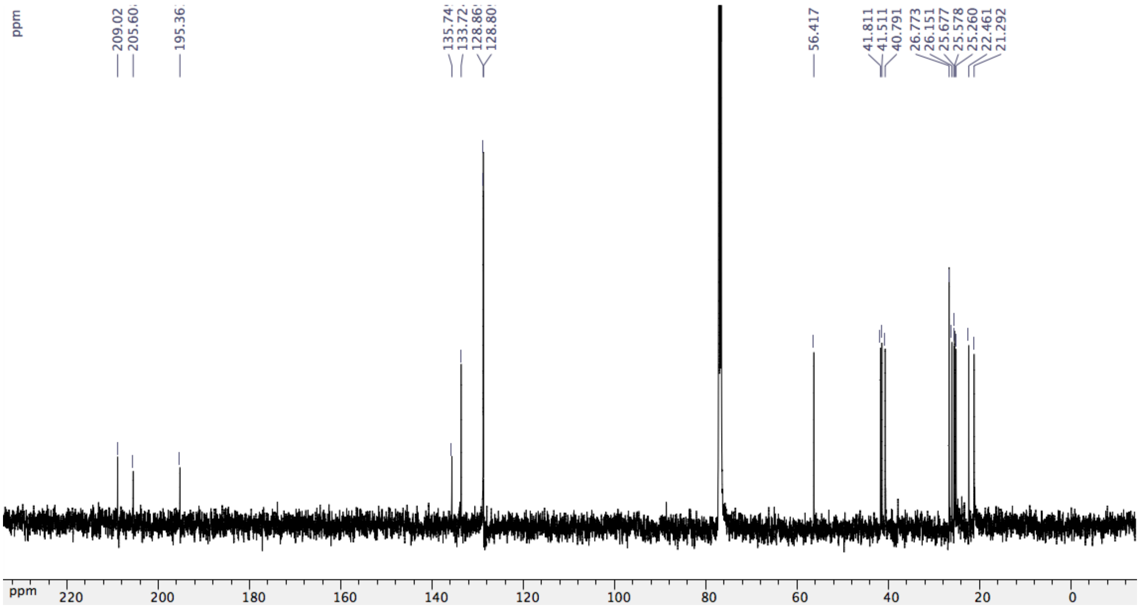

7-Benzoyl-1-hydroxybicyclo[3.2.1]octan-8-one (8)

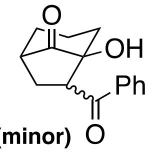

8 (minor)

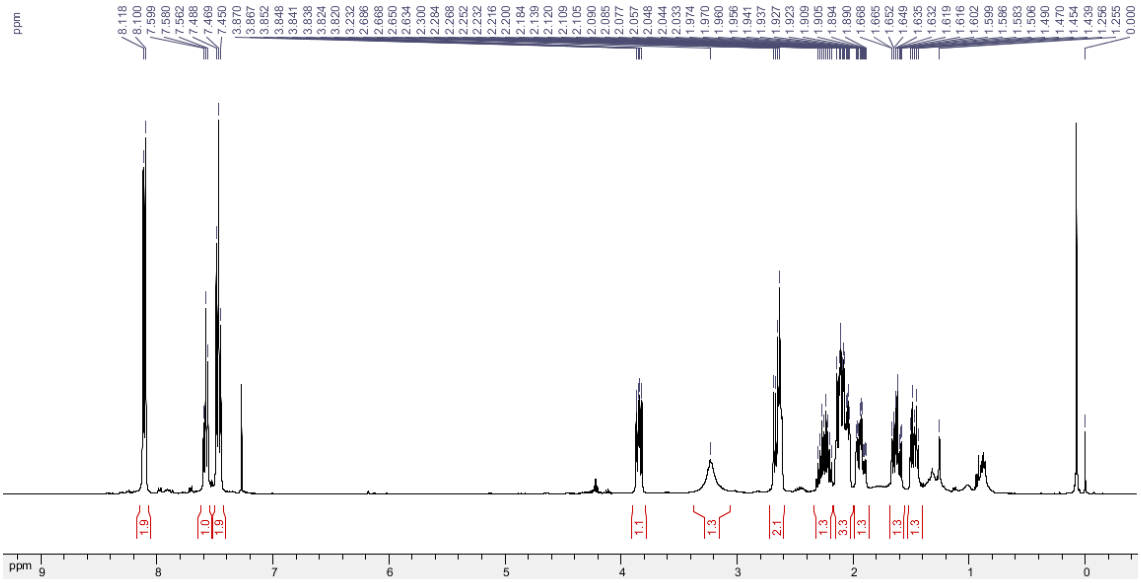

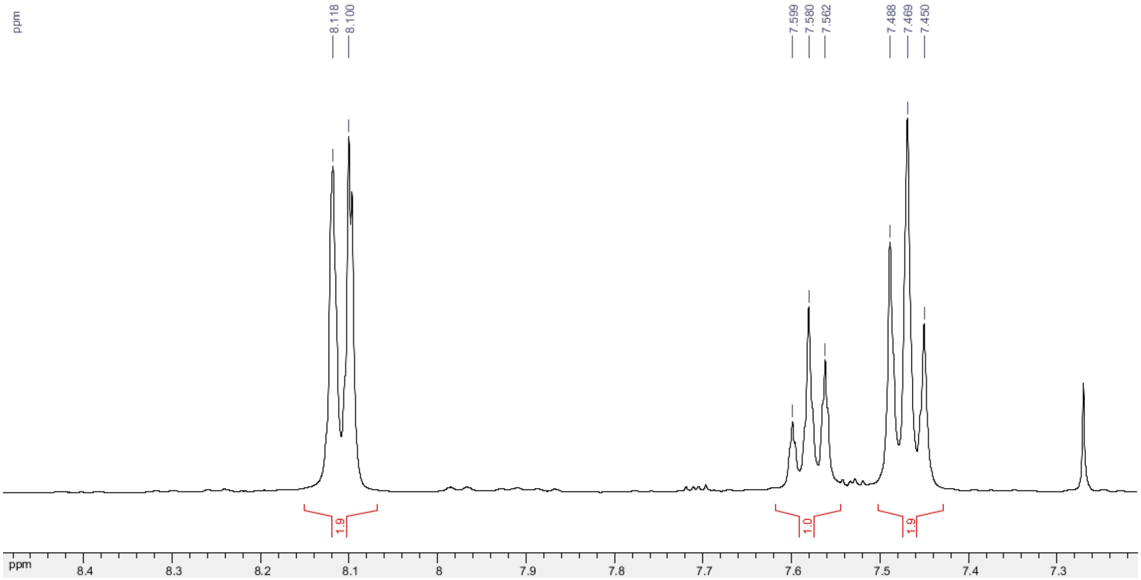

7-Benzoyl-1-hydroxybicyclo[3.2.1]octan-8-one (8) (minor)

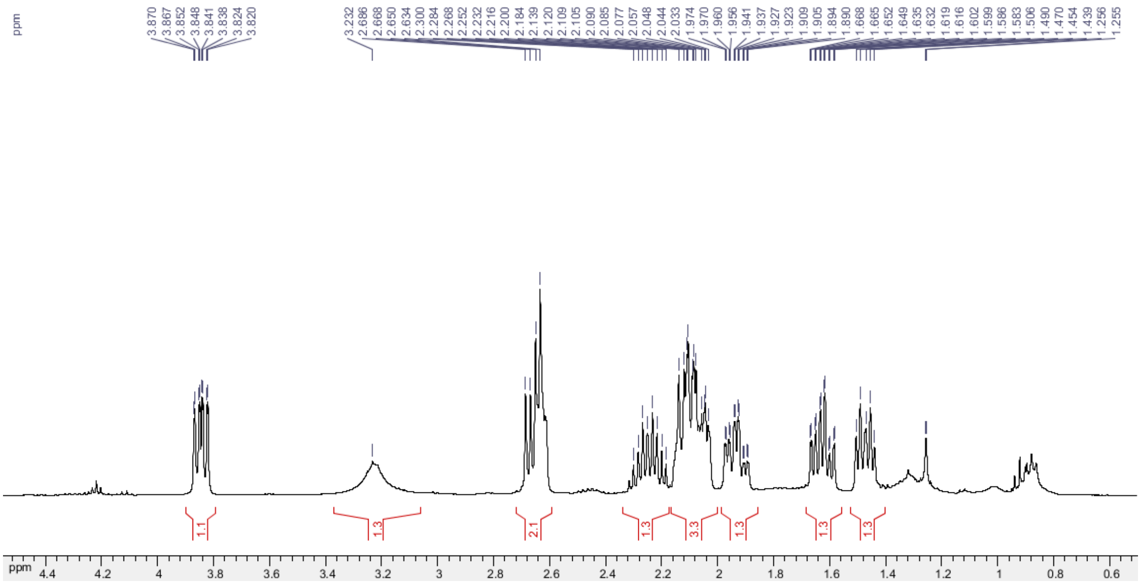

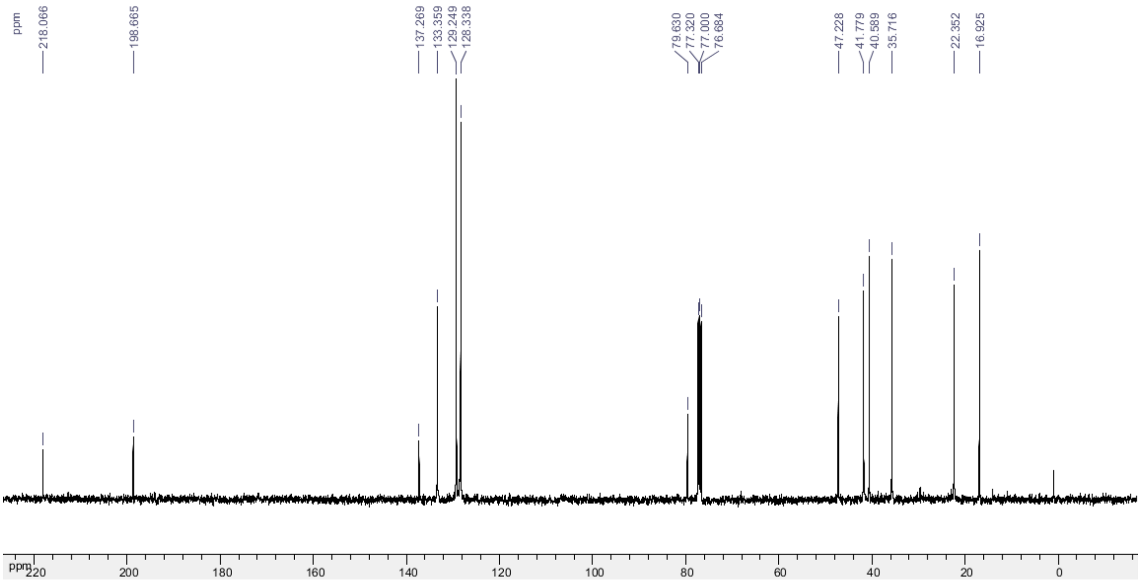

7-Benzoyl-1-hydroxybicyclo[3.2.1]octan-8-one (8)

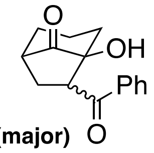

8 (major)

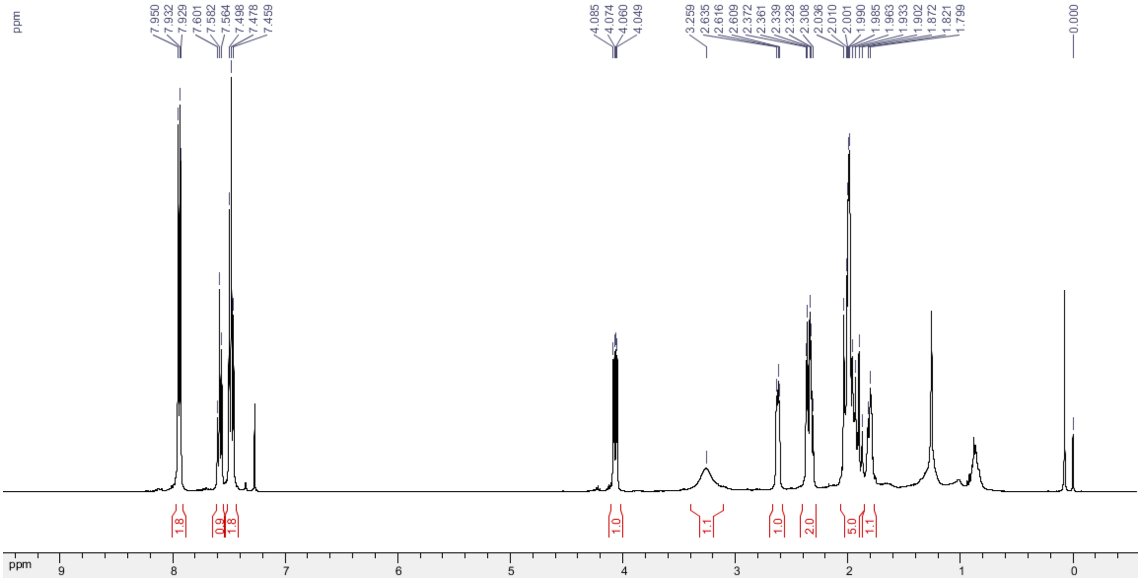

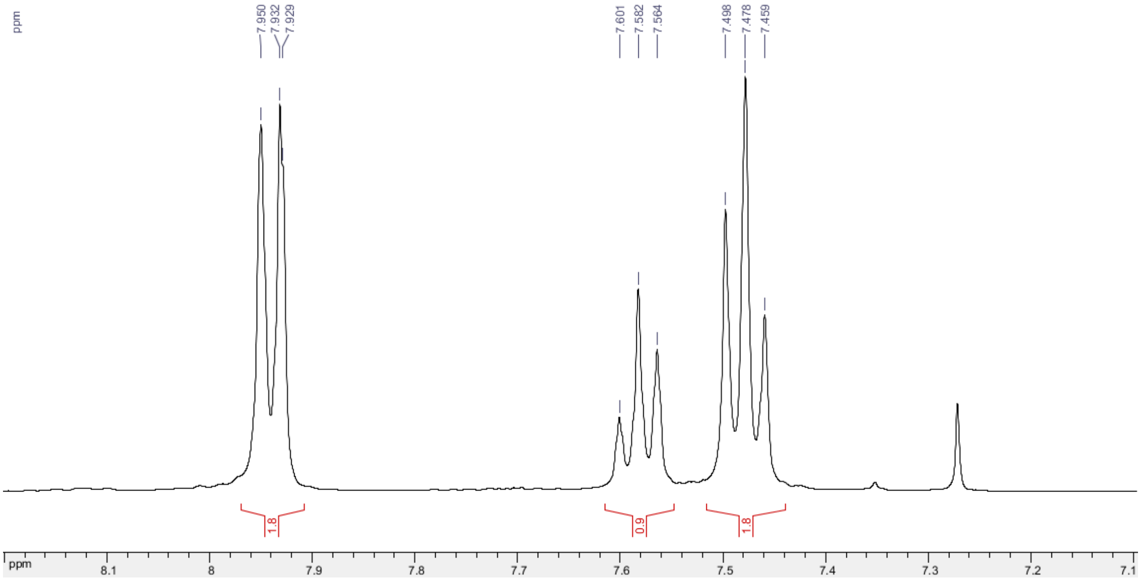

7-Benzoyl-1-hydroxybicyclo[3.2.1]octan-8-one (8) (major)

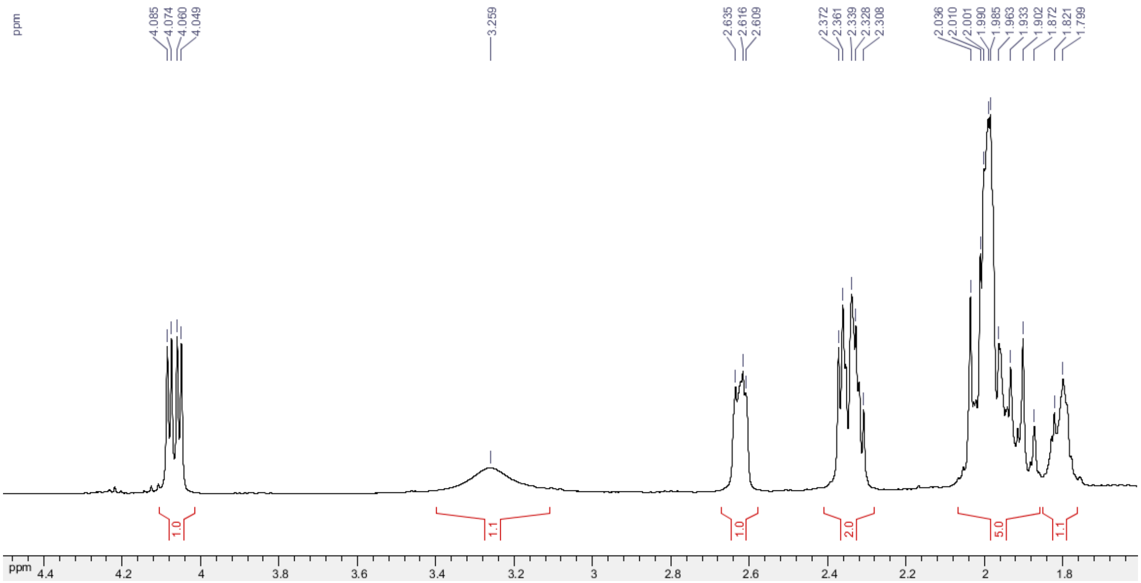

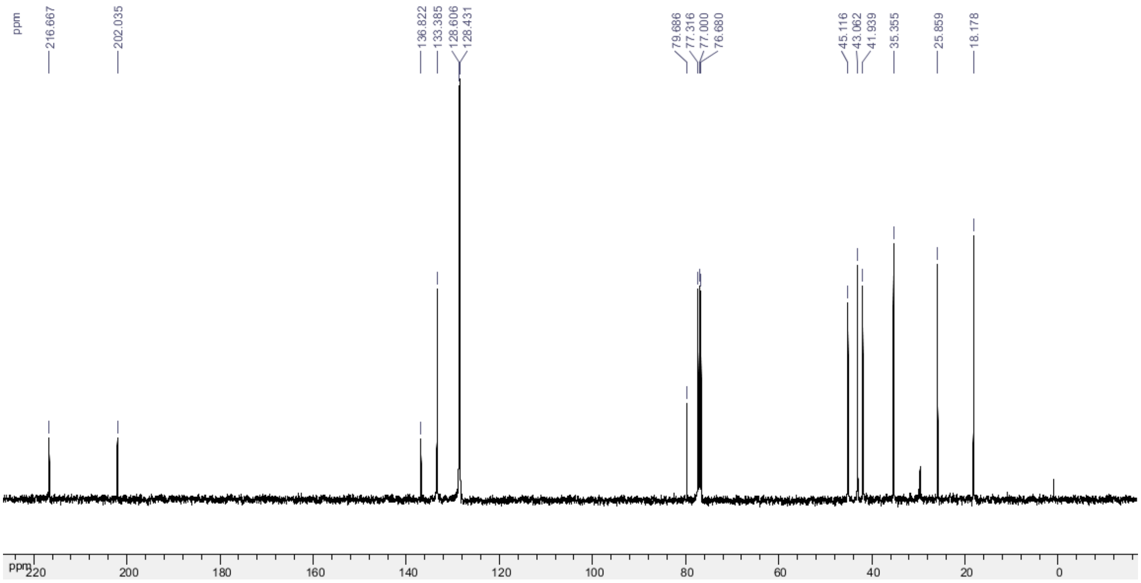

(*E*)-1-(3-Mesityl-3,4,5,6,7,8-hexahydro-2*H*-cyclohepta[*d*]thiazol-2-ylidene)-2-oxo-2-phenylethyl benzoate (10)

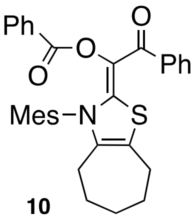

10

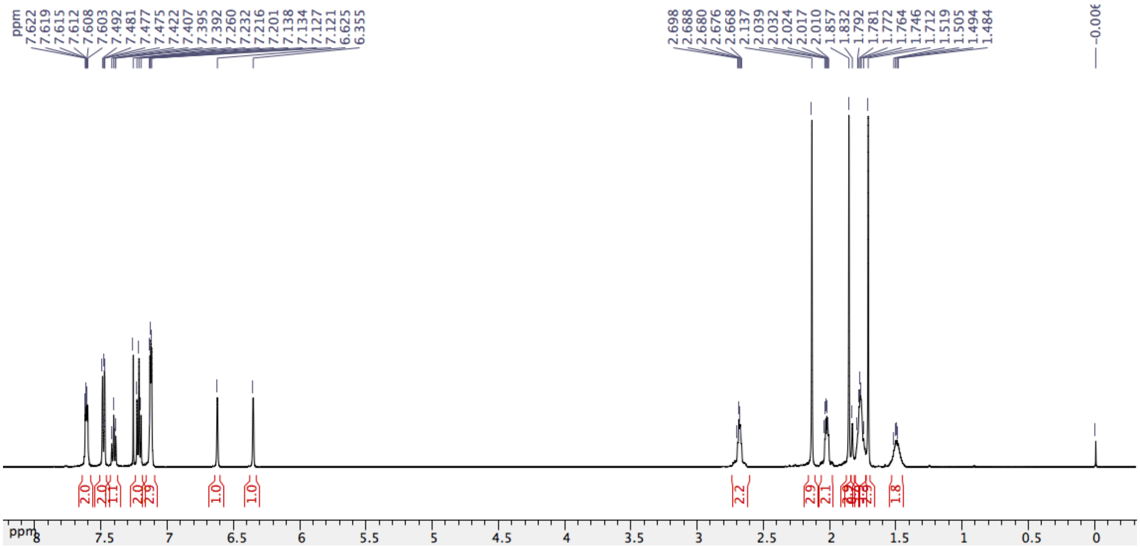

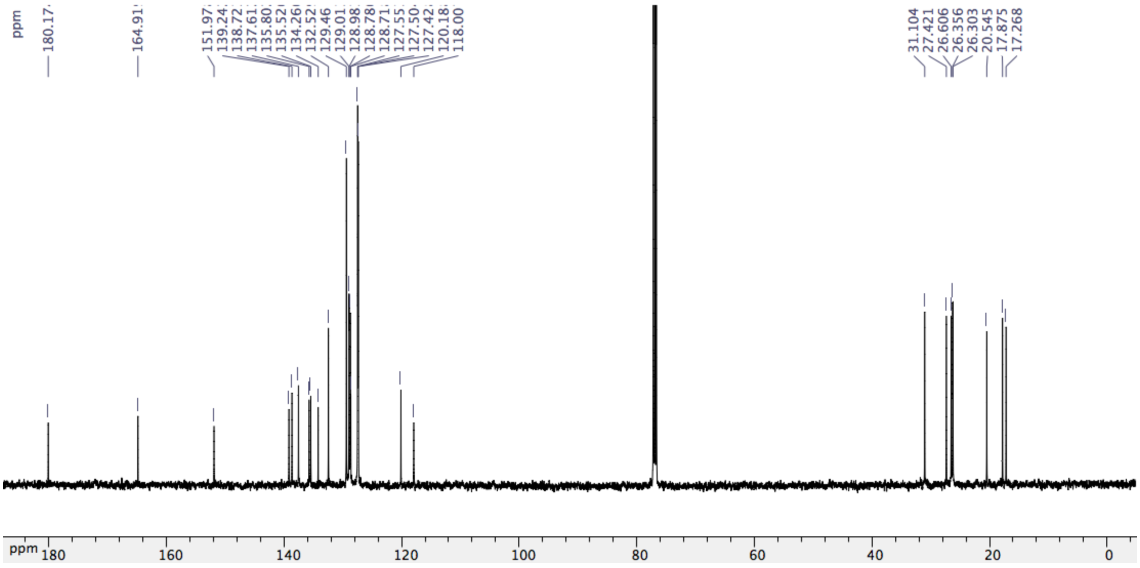

2-(((4-chlorobenzoyl)oxy)(4-chlorophenyl)methyl)-3-mesityl-5,6,7,8-tetrahydro-4*H* -cyclohepta[*d*]thiazol-3-ium perchlorate (12)

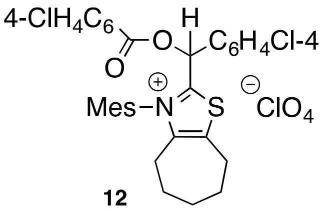

12

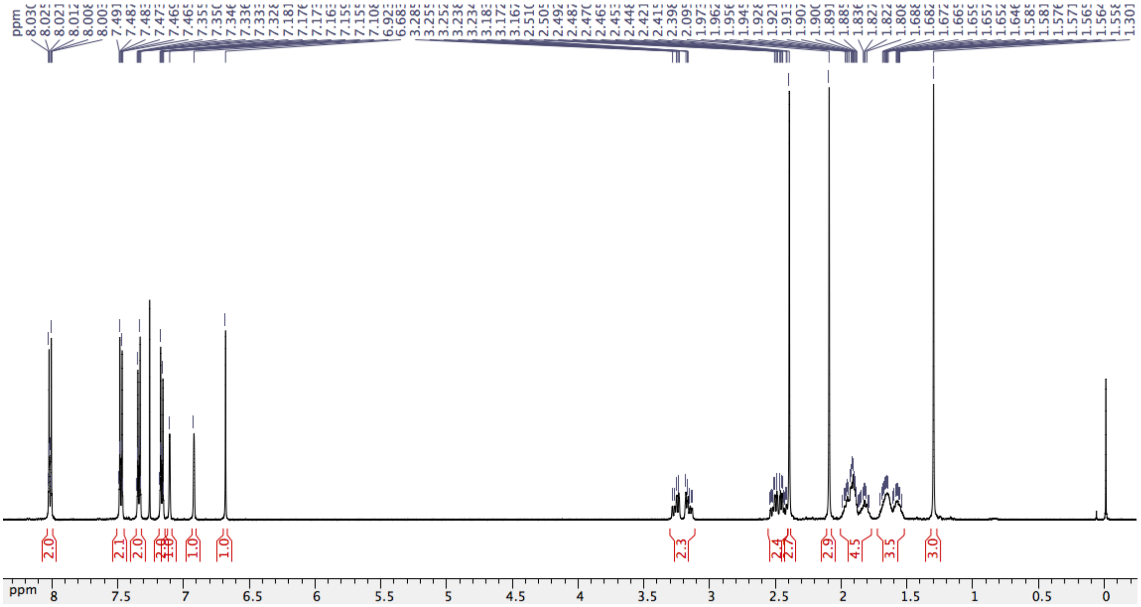

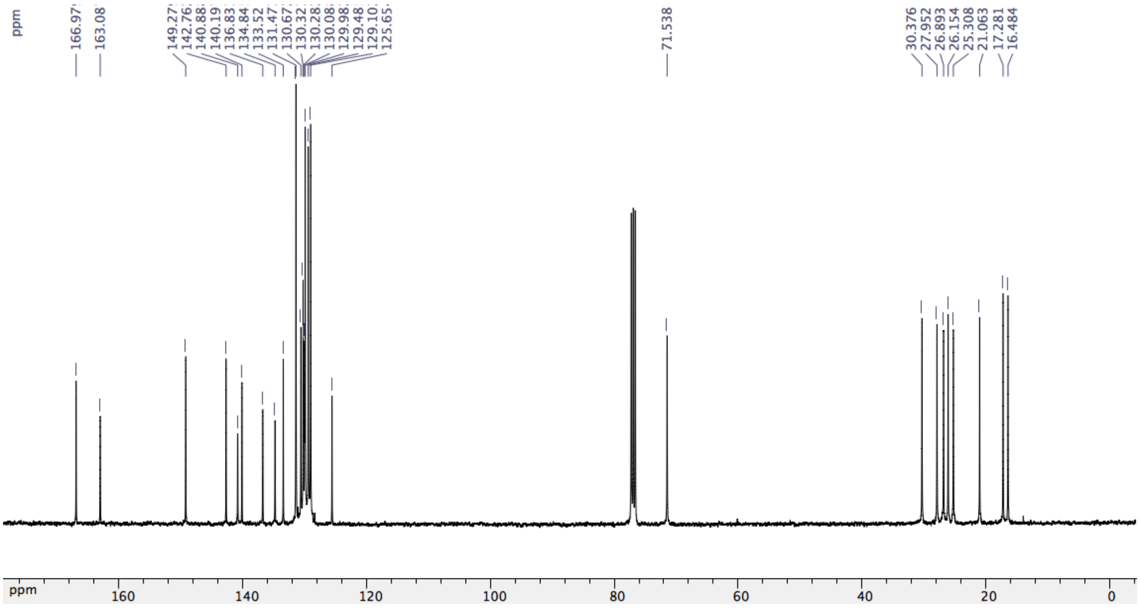

2-Benzoyl-1,4-bis(4-chlorophenyl)butane-1,4-dione (13)

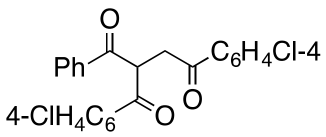

13

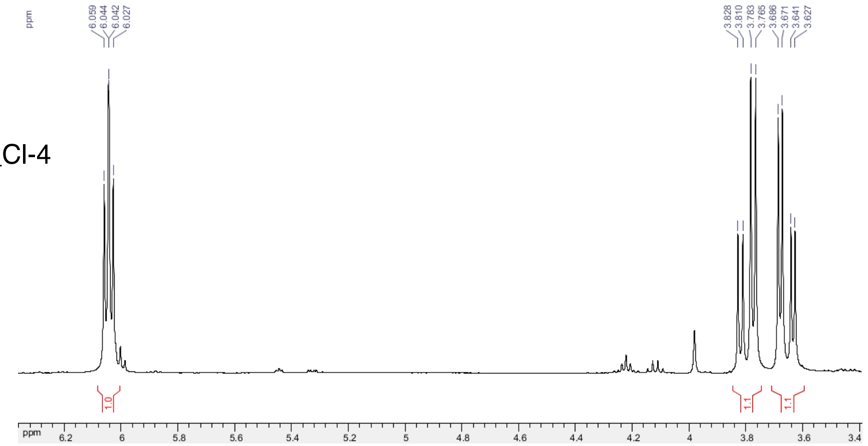

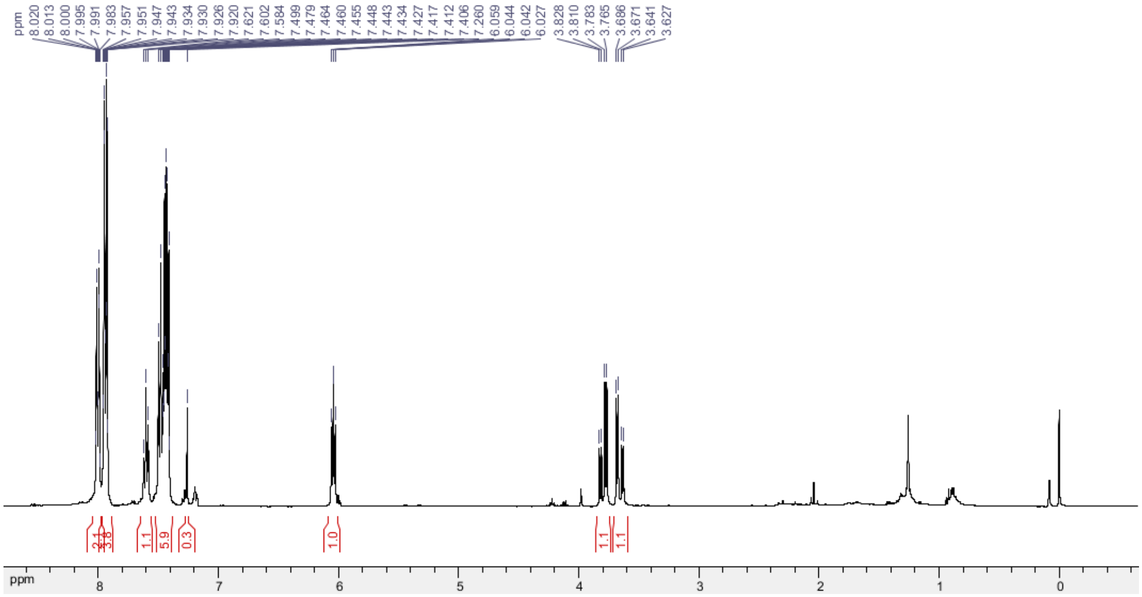

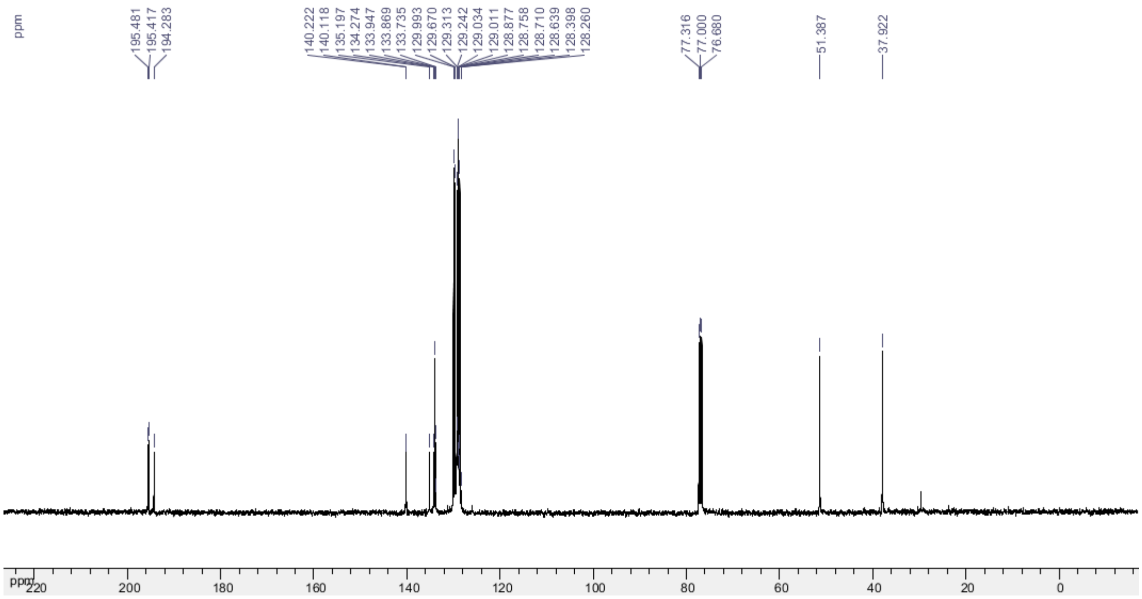

Supplement: File 1 — Experimental procedure, characterization data and copies of 1H and 13C NMR spectra of the products. [file Beilstein_J_Org_Chem-13-1816-s001.pdf]
